# Highly Efficient One-Pot Synthesis of COS-Based Block Copolymers by Using Organic Lewis Pairs

**DOI:** 10.3390/molecules23020298

**Published:** 2018-01-31

**Authors:** Jia-Liang Yang, Xiao-Han Cao, Cheng-Jian Zhang, Hai-Lin Wu, Xing-Hong Zhang

**Affiliations:** MOE Key Laboratory of Macromolecular Synthesis and Functionalization, Department of Polymer Science and Engineering, Zhejiang University, Hangzhou 310027, China; 11529008@zju.edu.cn (J.-L.Y.); 11629027@zju.edu.cn (X.-H.C.); 21629022@zju.edu.cn (C.-J.Z.); 21529003@zju.edu.cn (H.-L.W.)

**Keywords:** metal-free Lewis pairs, poly(monothiocarbonate) block copolymer, carbonyl sulfide, immortal polymerization

## Abstract

A one-pot synthesis of block copolymer with regioregular poly(monothiocarbonate) block is described via metal-free catalysis. Lewis bases such as guanidine, quaternary onium salts, and Lewis acid triethyl borane (TEB) were equivalently combined and used as the catalysts. By using polyethylene glycol (PEG) as the macromolecular chain transfer agent (CTA), narrow polydispersity block copolymers were obtained from the copolymerization of carbonyl sulfide (COS) and propylene oxide (PO). The block copolymers had a poly(monothiocarbonate) block with perfect alternating degree and regioregularity. Unexpectedly, the addition of CTA to COS/PO copolymerization system could dramatically improve the turnover frequency (TOF) of PO (up to 240 h^−1^), higher than that of the copolymerization without CTA. In addition, the conversion of CTA could be up to 100% in most cases, as revealed by ^1^H NMR spectra. Of consequence, the number-average molecular weights (*M*_n_s) of the resultant block copolymers could be regulated by varying the feed ratio of CTA to PO. Oxygen-sulfur exchange reaction (O/S ER), which can generate randomly distributed thiocarbonate and carbonate units, was effectively suppressed in all of the cases in the presence of CTA, even at 80 °C. This work presents a versatile method for synthesizing sulfur-containing block copolymers through a metal-free route, providing an array of new block copolymers.

## 1. Introduction

Driven by the pressure of petroleum depletion, research on the utilization of renewable biomass-derived [1,2,3] and one-carbon (C1) building blocks [4,5,6,7,8] for making polymers has drawn much attention in the past few decades. As a sulfur-containing analog of carbon dioxide (CO_2_), carbonyl sulfide (COS) is widely derived from the burning of fossil fuels, coal gas, and many chemical processes, and finally causes sulfur aerosols, fog, haze, acid rain, and damage of the ozonosphere via a series of photochemical reactions [9,10]. Similar to CO_2_ [11,12,13,14,15], COS can be used as a basic synthon for preparing sulfur-containing compounds and polymers. Unfortunately, the related works are rarely reported [6,16].

The copolymerization of COS with various epoxides (and oxetane) provided a series of functional poly(monothiocarbonate)s [17,18,19,20,21,22]. Introduction of sulfur atoms in the backbone brought polymers with remarkable optical property and heavy-metal recognition ability, which enabled them potentially to be used as photoconductive fibers and heavy-metal scavengers [10]. Since our first work on the alternating copolymerization of COS and propylene oxide (PO) [10], the conversion of COS to polymers has made significant advances [6,16]. However, reports on constructing block copolymers containing poly(monothiocarbonate) block are rare [20,23]. Ren and coworkers [20] reported the synthesis of a tri-block copolymer, poly(ethylene monothiocarbonate)-*b*-poly(propylene monothiocarbonate)-*b*-poly(ethylene monothiocarbonate) (PEMC-*b*-PPMC-*b*-PEMC), catalyzed by a highly active bifunctional (salen)Cr(III) complex with subsequent adding of monomers. Very recently, our group [23] reported a *ABA* block copolymer with precise structure, polystyrene-*b*-poly(propylene monothiocarbonate)-*b*-polystyrene (PS-*b*-PPTMC-*b*-PS) via atom transfer radical polymerization (ATRP) through several operation steps. Unfortunately, metal residues of the catalyst in these copolymers limited their potential applications [10,19,20,23]. Sulfur-containing block copolymers via metal-free route is urgently needed.

In 2017, we reported the copolymerization of COS with epoxides meditated by metal-free Lewis pairs [24]. A Lewis acid (LA), triethyl borane (TEB), was combined with various Lewis bases (LBs) such as amidine, guanidine, or quaternary onium salts to form Lewis pairs. They could catalyze the COS/epoxide copolymerization to produce poly(monothiocarbonate)s with perfect alternating degree and regioselectivity. Colorless and transparent copolymers were obtained, which overcome the drawbacks brought about by metal catalysts. However, it seemed that the time for LA and LB to form active Lewis pairs was uncertain, while the chain growth was rapid. Therefore, it is difficult to control the molecular weights and polydispersity (PDI) of the resulted copolymers on purpose [24], as well as build the block copolymers.

**Scheme 1:**
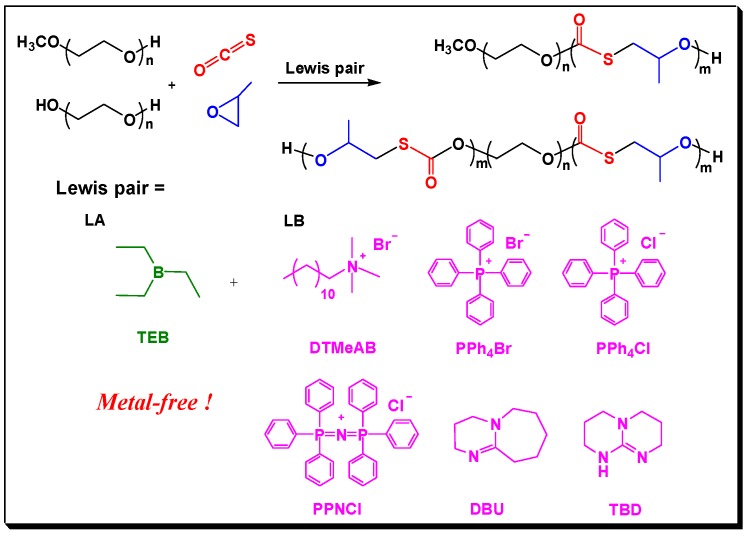
Lewis pair catalyzed immortal copolymerization process for synthesizing block copolymers containing PEG and poly(propylene monothiocarbonate) (PPMTC) block.

Presented by Inoue in 2000 [25], immortal polymerization was proven to be an effective way to build block copolymers with various topological structures and specialized function [26,27,28,29]. In 2010, Lee and coworkers [30] used (salen)cobalt(III) complex pending onium salts as the catalyst, polymers with –OH end groups as the CTAs for immortal copolymerization of CO_2_ with PO, afforded CO_2_-based block copolymers with precisely controlled molecular weights. As is disclosed by previous works, the (salen)cobalt(III) complex and organic Lewis pairs are similar Lewis acid–base systems [24,31,32,33,34]. Hence, we envisioned that Lewis pair polymerization would go through “immortal process” when CTAs were introduced (as shown in Scheme 1). We also expected that the immortal polymerization manner could mediate the molecule weights of the resultant block copolymers because fast chain transfer reaction often leads to predominant chain growth along the CTA molecules.

Herein, we report the copolymerization of COS with PO in the presence of CTAs by using organic Lewis pairs. Taking polyethylene glycol (PEG) and methoxy polyethylene glycol (MPEG) as the CTAs, *AB* and *ABA* block copolymers with poly(propylene monothiocarbonate) and PEG blocks were obtained with mediated molecule weights and narrow PDIs.

## 2. Results and Discussion

**Figure 1:**
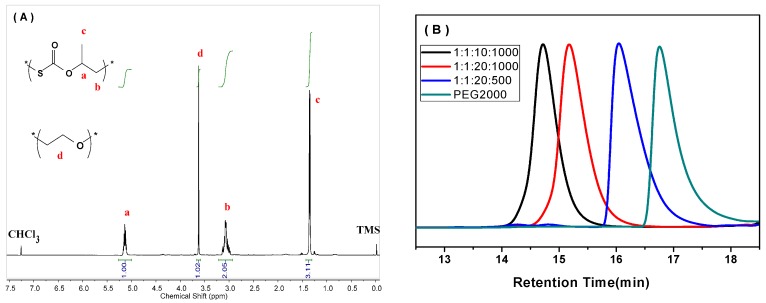
(**A**) Typical ^1^H NMR spectrum of the PPMTC-*b*-PEG-*b*-PPMTC (Entry 9 in Table 1); and (**B**) GPC curves of PPMTC-*b*-PEG-*b*-PPMTC (Entries 8, 9, 11 and 12, Table 1).

**Figure A1:**
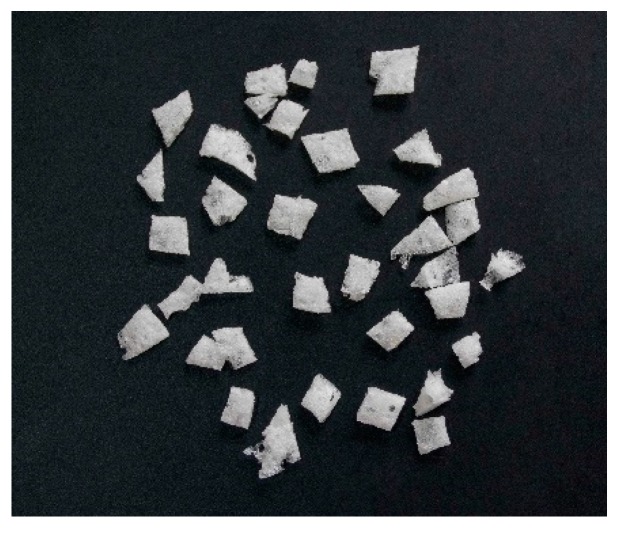
Purified PPMTC-*b*-PEG copolymer from immortal polymerization based on Lewis pair catalyst system.

The COS/PO copolymerization was successfully catalyzed by the TEB/DTMeAB pair in the presence of MPEG750 (M750) and PEG2000 (P2000) as CTAs in a variety of LA/LB/CTA/PO ratios, providing colorless di-block (*AB*) and tri-block (*ABA*) copolymers with well-defined structure (Figure A1 in Appendix A), and all the results are summarized in Table 1. No signal of polyether was observed in all the ^1^H NMR spectra (Figure 1A and Figures S1–S12), revealing a perfectly alternating structure of the PPMTC block. This is consistent with the superior activity of COS benefited from its strong electrophilicity [6], which eradicated successive insertion of PO. Much to our delight, in most cases, PPMTC was the major product over the cyclic product. Moreover, the TEB/DTMeAB pair exhibited excellent selectivity in the nucleophilic attack process, where the propagating species selectively attacked the less sterically hindered methylene carbon of PO. As a result, greater than 99% of PPMTC linkages were tail-to-head (T-H) structure (Figures S1–S11), and higher than that of (salen)CrX complexes used in the COS/PO copolymerization [10]. In addition, oxygen/sulfur exchange reaction (O/S ER), which can generate randomly distributed thiocarbonate and carbonate units, was thoroughly depressed in all the copolymerization processes at 40 °C, based on the ^13^C NMR spectra of the purified products (Figures S1–S12).

**Scheme 2:**
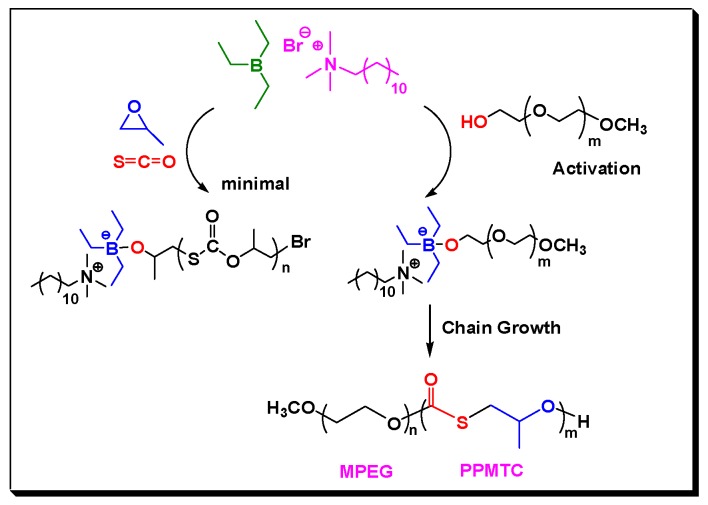
Proposed mechanism of the formation of block copolymers catalyzed by Lewis pair in the presence of MPEG.

Remarkably, the catalytic activity of Lewis pairs for COS/PO copolymerization in the presence of CTA is clearly higher than those in the absence of CTA [24]. PO was nearly completely consumed at 40 °C within 8 h, even when the feeding ratio of [PO]:[Lewis pair] was up to 2000:1; therefore, a turnover frequency (TOF) as high as 240 h^−1^ was observed (Entry 12, Table 1). We suspected that TEB is highly electron-deficient Lewis acid that is similar to typical salen metal complexes [30] and can activate the end –OH group of PEG rapidly via a similar chain transfer reaction process, to form a (Ethyl)_3_B-*O*-PEG growing species with improved nucleophilicity, which was also stabilized by the cations from DTMeAB (Scheme 2). Since the feed ratio of CTA/LB were 5/1–50/1, such activation process for PEG was predominant, which favored the rapid formation of more amounts of anionic tetrahedral boron species, and thus improved catalytic activity.

CTAs with one or two hydroxy end groups functioned well in regulating the molecule weights and distributions of the copolymers. More than 90% of CTA could participate in the initiation in all cases in Table 1, and CTA conversion was up to 99% in some conditions (Entries 1, 2, 5–7, 9, 11 and 12 in Table 1). As for M750, when the feeding ratio of [LA]:[LB]:[CTA]:[PO] was 1:1:5:1000, PEG-*b*-PPMTC with a *M*_n_ of 27.6 kg/mol was achieved with a polydispersity index (PDI) of 1.2. Kept the feed ratio of LA, LB and PO unvaried, decreasing the feed ratios of [Lewis pair]:[CTA] from 1:10 to 1:50, *M*_n_s of resultant block copolymers were observed as 14.7, 7.1 and 3.8 kg/mol, respectively, which displays a linear decline in *M*_n_s with narrow PDIs of 1.1 (Entries 1–4 in Table 1). Furthermore, we could also mediate the *M*_n_s of the resultant di-block copolymers by simply increasing the amounts of PO in the fixed feed ratio of Lewis pair to CTA (1:20) (Entries 5, 3, and 6 in Table 1). These results indicate that the molecular weights of the resultant block copolymers could be mediated in the presence of CTAs.

We studied the synthesis of *ABA* tri-block copolymer by using P2000 with two hydroxy end groups as the CTA. A linear decline of *M*_n_s from 51.0 to 6.6 kg/mol was obtained by extending the ratio of CTA by ten times (Entries 7–10 in Table 1). When the feeding ratio of [Lewis pair]:[P2000] was 1:10 or 1:20, the resultant copolymers displayed unimodal GPC curves (Figure 1B). By way of contrast, bimodal GPC distribution was often seen in synthesizing CO_2_-based polycarbonate block copolymers from the copolymerization of PO and CO_2_ in the presence of CTAs, even with the [CTA]/[catalyst] ratio of less than 1/50 [30], meant that the chain initiation from the catalyst clearly coexisted. Therefore, the TEB/DTMeAB pair was more favorable to the chain transfer reaction with PEG with end –OH groups. In this scenario, the direct activation of PO and COS by Lewis pair could be dramatically inhibited. The generated (Ethyl)_3_B-O-PEG was mainly initiated the copolymerization, as shown in Scheme 2.

We further investigated the impact of the reaction temperature on the synthesis of the block copolymers, since previous reports [10,35] disclosed that the O/S ER was very sensitive to the reaction temperatures, whatever organic or metal catalysts were used. As summarized in Table 2, for all copolymerizations, either MPEG or PEG was used as CTAs, no O/S ER products were observed in a temperature range of 40–80 °C. In contrast, our previous works revealed that the dithiocarbonate [–SC(=O)S–] and carbonate [–OC(=O)O–] linkages generated considerably in the copolymer by using (salen)Cr(III)Cl complex at 60 °C [10]. While for the COS/PO copolymerization by using similar organic Lewis pairs at temperatures higher than 70 °C [24], cyclic products, 5-methyl-1,3-oxathiolan-2-one and 4-methyl-1,3-dithiolan-2-one were predominantly produced due to severe O/S ER. Clearly, introducing PEG with –OH groups into the TEB/DTMeAB pair could effectively inhibit the occurrence of O/S ER, even at a high reaction temperature of 80 °C. Being different from the formation of metal-OH bond that was the decisive factor for O/S ER [10,35] in metal-catalyzed copolymerization system, herein, the (Ethyl)_3_B-OR structure resulted from the fast chain transfer to PEG impeded the generation of possible (Ethyl)_3_B-OH bond. Note that elevating the reaction temperature led to relative low copolymer selectivity, for example, the copolymer selectivity of COS/PO copolymerization at 60 and 80 °C were 87% and 84%, respectively, in the presence of M750 (Entries 1 and 2 in Table 2).

The impact of *M*_n_s of CTA on synthesizing the block copolymers was also studied. CTAs with high *M*_n_s, such as MPEG2000 (M2000) and MPEG5000 (M5000) performed well for the copolymerization even in a low feeding ratio (Entries 5–8 in Table 2). This demonstrated that more volume percentage of PEG block could be introduced into the block copolymer and thus enhanced their hydrophilicity. Unfortunately, when M5000 was added 10 (or 20) times of Lewis pair, CTA conversion of only 32% (or 16%) was observed due to its poor solubility in the reaction system (Entries 9 and 10 in Table 2).

Next, we conducted COS/PO copolymerization by using various Lewis pairs in the presence of M750, as shown in Table 3. All the resultant copolymers had a PPMTC block with perfectly alternating degree (Figures S25–S29). Onium salts, such as bis(triphenylphosphine) iminium chloride (PPNCl), tetraphenylphosphonium chloride (PPh_4_Cl) and tetraphenylphosphonium bromide (PPh_4_Br) exhibited similar catalytic activity (229–237 h^−1^) when coupled with equimolar TEB, and M750 was converted completely in these examples (Entries 1–3 in Table 3). Size of the cations (PPNCl vs. PPh_4_Cl, Entries 1 and 2 in Table 3) and the nucleophilicity of the anion (Br^−^ vs. Cl^−^, Entries 2 and 3 in Table 3) showed minimal differences on the copolymerization activity. While PPNCl was used as LB, relative low copolymer selectivity (84%) was observed. The organic bases 1,8-diazabicyclo[5.4.0]undec-7-ene (DBU) and 1,5,7-triazabicyclododecene (TBD) could also couple with TEB for the copolymerization, and well-defined block copolymers were obtained successfully, with a little harm in conversion of M750. However, 4-dimethylaminopyridine (DMAP) with strong basicity accompanied with TEB failed to catalyze the copolymerization, whether CTA was added or not. Totally, in the presence of CTA, intramolecular Lewis pair (e.g., DBU/TEB and TBD/TEB) resulted in poorer CTA conversion (Entries 4 and 5 in Table 3) than the intermolecular Lewis pairs (DTMeAB/TEB and PPNCl/TEB) [24]. We suspected that onium salts with less basicity favored the activation process of CTA by TEB (Scheme 2).

**Figure 2:**
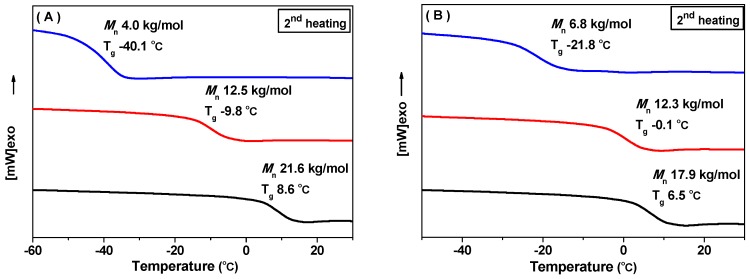
(**A**) DSC curves (second heating) of PPMTC-*b*-PEG-*b*-PPMTC with various *M*_n_s (Entries 8, 9, and 11 in Table 1); and (**B**) DSC curves (second heating) of PPMTC-*b*-MPEG with various *M*_n_s (Entries 5–7 in Table 2).

Finally, thermal properties of the block copolymers are presented. The block copolymers presented good thermal stability (Entry 3 in Table 2, and Figure S30) with an initial decomposition temperature (*T*_d_) of 230 °C, which is clearly higher than pure PPTMC with a *T*_d_ of ca. 137 °C [10]. Of interest, both di-block and tri-block copolymers displayed only one glass transition temperature (*T*_g_), revealing the good compatibility of the two blocks of PPMTC and PEG. Correspondingly, no crystallization point (*T*_m_) of PEG block was observed in all of the DSC curves. Presumably, the crystallization of PEG in the block copolymer was confined by PPMTC block. *T*_g_s of the block copolymer increased with the length of the PPMTC block, as the chain stiffness of PPMTC (*T*_g_: 30.0 °C) [16] is stronger than that of PEG (*T*_g_: −61.3 °C). *T*_g_s of MPEG2000-*b*-PPMTC increased from −40.1 to 8.6 °C as the overall *M*_n_s increased from 4.0 to 21.6 kg/mol (Figure 2A). As for PPMTC-*b*-MPEG2000-*b*-PPMTC, the increase of *T*_g_ from −21.8 to 6.5 °C was observed with increasing the length of the PPMTC blocks (Figure 2A).

## 3. Materials and Methods

### 3.1. Materials

Unless otherwise specified, all syntheses and manipulations were carried out on a double-manifold Schlenk vacuum line under nitrogen atmosphere or in a nitrogen-filled glove box. Following purification, materials were stored in a nitrogen-filled glovebox prior to use unless otherwise specified. Triethyl borane (TEB) in tetrahydrofuran solution (1.0 mol/L) was purchased from Sigma-Aldrich and used without further purifications. Dodecyltrimethylammonium bromide (DTMeAB), 1,5,7-Triazabicyclo [4.4.0] dec-5-ene (TBD), Tetraphenylphosphonium chloride (PPh_4_Cl), Bis(triphenylphosphine)iminium chloride (PPNCl), and Tetraphenylphosphonium bromide (PPh_4_Br) were dried in a vacuum oven at 100 °C for overnight. 1,8-diazabicyclo [5.4.0] undec-7-ene (DBU) was purified by distillation after stirring with calcium hydride for 3 days. Polyethylene glycol 2000 (P2000), methoxy polyethylene glycol 750 (M750), methoxy polyethylene glycol 2000 (M2000) and methoxy polyethylene glycol 5000 (M5000) were azeotropic dried with toluene, and preserved in glove box. Propylene oxide (PO) was purified by distillation after stirring with calcium hydride for 3 days. Tetrahydrofuran (THF) were distilled from sodium/benzophenone mixture before used. Carbonyl sulfide (COS) (99.9%, ACS Grade) was purchased from the APK (Shanghai, China) Gas Company LTD and used directly.

### 3.2. Copolymerization of COS with Epoxides in the Presence of CTAs, Experimental Details

A 10-mL autoclave with magnetic stirrer was first dried in an oven at 120 °C overnight, then immediately placed into the glove box chamber. After keeping under vacuum for 2–3 h, the reaction vessel was moved into the glovebox with nitrogen atmosphere. The copolymerization of COS with PO described below is taken from Entry 1 in Table 1 as an example. *N,N,N*-trimethyl-1-dodecanaminium bromide (DTMeAB, 6.5 mg, 0.021 mmol) and PEG750 (78.8 mg, 0.105 mmol) was firstly added into the reactor and dissolved in 3.0 mL of THF. PO (1.5 mL, 21 mmol) was carefully added into the vessel after the introduction of an appropriate amount of TEB (21 μL, 0.021 mmol). The reactor was sealed and taken out from the glovebox and charged with appropriate pressure of COS. The copolymerization was carried out at 40 °C for 8 h. The reactor was then cooled in ice-water bath, the unreacted COS was slowly vented, and an aliquot was then taken from the resulting crude product for the determination of the ratio of copolymer/cyclic products by ^1^H NMR spectrum. The crude product was quenched with acetic acid in water (1 mol/L). The collected product was dissolved with CH_2_Cl_2_ and then precipitated in n-hexane. The product was collected by centrifugation and dried in vacuum at 40 °C overnight.

### 3.3. Characterization

#### 3.3.1. Nuclear Magnetic Resonance (NMR)

All ^1^H and ^13^C NMR spectra were recorded on a Bruker AVANCE DMX 400 MHz instrument in CDCl_3_. Chemical shift values were referenced to CHCl_3_ at 7.26 ppm for ^1^H NMR and 77.16 ppm for ^13^C NMR. Molecular weights and molecular weight distributions of the resultant copolymers were determined with a PL-GPC220 chromatograph (Polymer Laboratories) equipped with an HP 1100 pump from Agilent Technologies. The GPC columns were eluted with THF with 1.0 mL/min at 40 °C. The sample concentration was 0.4 wt %, and the injection volume was 50 μL. Calibration was performed using monodisperse polystyrene standards covering the molecular-weight range from 500 to 5,000,000 Da.

#### 3.3.2. Differential Scanning Calorimetry (DSC)

The glass transition temperatures (*T*_g_) of the resultant copolymers were determined by using a TA Q200 instrument. The samples were heated from room temperature to 150 °C at a rate of 10 °C/min under nitrogen atmosphere, kept for 2 min, and then cooled to −80 °C in a cooling rate of −10 °C/min, finally heated from −80 to 150 °C at a heating rate of 10 °C/min. As the quantity of heat for motion of repeating unit is different for polymers before and after glass transition, there will be a step in the DSC curve, and the temperature is determined as *T*_g_. In this work, *T*_g_ was determined from the second heating curves.

#### 3.3.3. Thermogravimetric Analysis (TGA)

The initial decomposition temperature (*T*_d_) of the block copolymer was determined by using TA Q50 instrument. The sample was heated from 40 to 500 °C at a rate of 10 °C/min under nitrogen atmosphere. Temperature when the mass loss is five percent was taken as *T*_d_.

### 3.4. Calculation of TOF, Conversion of CTAs and Copolymer Selectivity

Copolymer selectivity was calculated based on the ^1^H NMR spectrum of the crude product. Taking Entry 1 in Table 2 as an example, spectrum of the crude product is shown in Figure S13. Protons with chemical shift of 4.80, 3.51, 3.23 and 1.53 ppm belong to the methenyl, methene and methyl of cyclic product, respectively, and the corresponding peaks at 5.13, 3.07 and 1.36 ppm belong to these groups in the backbone of the copolymer. The area ratio of these two parts was taken as the copolymer selectivity. Together with the mass of pure product, we can precisely get the TOF values. As for conversion of CTA, we used ^1^H NMR spectra before and after precipitation to calculate. PPMTC block from COS and PO is hydrophobic, while PEG block has good solubility, as a result, after precipitation in water, CTA without poly(monothiocarbonate) linkage was removed, so we can get conversion of CTA by the area variation in ^1^H NMR spectra. Taking Entry 1 in Table 2 as an example, set the peak area of methyl in PPMTC as 1, the peak area of methane of M750 was 1.67 and 1.60. respectively, and the relative ratio of these two areas was taken as conversion of CTA.

## 4. Conclusions

In conclusion, we described the copolymerization of COS and PO in the presence of polyethylene glycol catalyzed using metal-free Lewis pairs. PPMTC-*b*-PEG and PPMTC-*b*-PEG-*b*-PPMTC were achieved with perfectly alternating degree and regioregularity. Various Lewis pairs were proven to be active when CTA was added, and this immortal polymerization could be carried out up to 80 °C without observing O/S ER. A remarkably high TOF of 240 h^−1^ for COS/PO copolymerization was achieved in the presence of CTAs. The molecule weight of resultant block copolymers could be mediated by varying the feed ratio of CTA and monomers with narrow PDI (1.1). Therefore, combination of metal-free Lewis pair catalyst and immortal polymerization not only enables the resultant copolymers to be obtained free of metal residues, widening the application, but also makes conveniently various sulfur-containing block copolymers via one-step one-pot manner. Our ongoing work is to prepare various micelles with different morphologies using these amphipathic copolymers.

## Figures and Tables

**Table 1:** Fully alternating copolymerization of COS with PO in the presence of CTAs ^1^.

Entry	CTA	LA/LB/CTA/PO	TOF (h^−1^) ^2^	Conv. of CTA (%) ^3^	Copolym. Select. (%) ^4^	*M*_n_ (kg/mol) ^5^	PDI (*M*_w_/*M*_n_) ^5^
1	M750	1:1:5:1000	117	>99	100	27.6	1.2
2	M750	1:1:10:1000	115	>99	97	14.7	1.2
3	M750	1:1:20:1000	120	92	100	7.1	1.1
4	M750	1:1:50:1000	123	95	100	3.8	1.1
5	M750	1:1:20:500	60	>99	100	5.9	1.1
6	M750	1:1:20:2000	232	>99	100	23.8	1.2
7	P2000	1:1:5:1000	115	>99	100	51.0	1.2
8	P2000	1:1:10:1000	117	97	100	21.6	1.1
9	P2000	1:1:20:1000	122	>99	100	12.5	1.1
10	P2000	1:1:50:1000	124	94	100	6.6	1.3
11	P2000	1:1:20:500	59	>99	100	4.0	1.1
12	P2000	1:1:20:2000	240	>99	100	21.9	1.1

^1^ The reactions were performed in THF (3.0 mL) by using Lewis pair composed of TEB and dodecyltrimethylammonium bromide (DTMeAB) as the catalyst, and different PEG (COS:PO = 2:1, molar ratio) in a 10-mL autoclave at 40 °C for 8 h. All the resulting copolymers were fully alternating, revealed by ^1^H NMR spectroscopy (Figures S1–S12). No O/S ER products were observed in all samples, confirmed by ^13^C NMR spectroscopy (Figures S1–S12). ^2^ (Mol epoxide consumed)/(mol TEB h). ^3^ Determined by using ^1^H NMR spectroscopies of the crude product and the purified one. ^4^ Determined using ^1^H NMR spectroscopy. The copolymer selectivity is the molar ratio of the monothiocarbonate unit to the cyclic product. ^5^ Determined by gel permeation chromatography in THF (40 °C), calibrated with polystyrene standards.

**Table 2:** Effect of different influence factors in the copolymerization ^1^.

Entry	CTA	LA/LB/CTA/PO	TOF (h^−1^) ^2^	Conv of CTA (%) ^3^	Copolymer Selectivity (%) ^4^	*M*_n_ (kg/mol) ^5^	PDI (*M*_w_/*M*_n_) ^5^
1 ^6^	M750	1:1:20:1000	122	96	87	6.5	1.2
2 ^7^	M750	1:1:20:1000	123	98	84	7.1	1.1
3 ^6^	P2000	1:1:20:1000	119	>99	100	11.5	1.1
4 ^7^	P2000	1:1:20:1000	124	99	86	10.9	1.2
5	M2000	1:1:5:1000	121	>99	95	17.9	1.3
6	M2000	1:1:10:1000	117	99	100	12.3	1.2
7	M2000	1:1:20:1000	122	>99	100	6.8	1.1
8	M5000	1:1:5:1000	98	>99	100	21.6	1.1
9	M5000	1:1:10:1000	68	32	100	23.3	1.7
10	M5000	1:1:20:1000	56	16	100	33.5	1.3

^1^ The reactions were performed in THF (3.0 mL) by using Lewis pair of TEB/DTMeAB as the catalyst, and different PEG as CTA (COS:PO = 2:1, molar ratio) in a 10-mL autoclave at 40 °C for 8 h. All the resulting copolymers were fully alternating revealed by ^1^H NMR spectroscopy (Figures S13–S24). No O/S ER products were observed in all of the results, as confirmed by ^13^C NMR spectroscopy (Figures S13–S24). ^2^ (Mol epoxide consumed)/ (mol TEB h). ^3^ Determined by using ^1^H NMR spectroscopies of the crude product and purified one. ^4^ Determined by using ^1^H NMR spectroscopy. The polymer selectivity is the molar ratio of the monothiocarbonate unit to the cyclic product. ^5^ Determined by gel permeation chromatography in THF (40 °C), calibrated with polystyrene standards. ^6^ Reaction temperature was 60 °C. ^7^ Reaction temperature was 80 °C.

**Table 3:** Copolymerization of COS with PO by using different LBs in the presence of M750 ^1^.

Entry	LB	TOF (h^−1^) ^2^	Conv of CTA (%) ^3^	Copolymer Selectivity (%) ^4^	*M*_n_ (kg/mol) ^5^	PDI (*M*_w_/*M*_n_) ^5^
1	PPNCl	237	99	84	17.9	1.3
2	PPh_4_Cl	234	>99	95	16.1	1.2
3	PPh_4_Br	229	98	94	14.8	1.2
4	DBU	202	94	84	11.4	1.2
5	TBD	214	91	90	14.7	1.3
6	DMAP	0	-	-	-	-

^1^ The reactions were performed in THF (3.0 mL) by using Lewis pair composed of TEB and various LBs as the catalysts, M750 was used as a CTA ([LA]:[LB]:[CTA]:[PO]:[COS] =1:1:20:2000:4000, molar ratio) in a 10-mL autoclave at 40 °C for 8 h, all the resulting copolymers were fully alternating, as revealed by ^1^H NMR spectroscopy (Figures S25–S29). No O/S ER products were observed in any of the results, confirmed by ^13^C NMR spectroscopy (Figures S25–S29). ^2^ (Mol epoxide consumed)/(mol TEB h). ^3^ Determined by using ^1^H NMR spectroscopies of the crude product and purified one. ^4^ Determined by using ^1^H NMR spectroscopy. The polymer selectivity is the molar ratio of the monothiocarbonate unit to the cyclic product. ^5^ Determined by gel permeation chromatography in THF (40 °C), calibrated with polystyrene standards.

## Supplementary Materials

Figures S1–S30 are available online.
