# Supplementary material for: Highly Efficient One-Pot Synthesis of COS-Based Block Copolymers by Using Organic Lewis Pairs
Source: Molecules. 2018 Jan 31;23(2):298. doi: 10.3390/molecules23020298 (PMC6017417; doi:10.3390/molecules23020298)
Supplement: Supplementary file 1 [file molecules-23-00298-s001.pdf]

Electronic Supporting Information

Highly efficient one-pot synthesis of COS-based block copolymers by using organic Lewis pairs

Jia-Liang Yang, Xiao-Han Cao, Cheng-Jian Zhang, Hai-Lin Wu and Xing-Hong Zhang*

[1] Department of Polymer Science and Engineering, MOE Key Laboratory of Macromolecular Synthesis and Functionalization Zhejiang University, Hangzhou 310027 (China); 11529008@zju.edu.cn (J. Y.) 11629027@zju.edu.cn (X. C.); 21629022@zju.edu.cn (C. Z.); 21529003@zju.edu.cn (H. W.); xhzhang@zju.edu.cn (X. Z.);

* Correspondence: xhzhang@zju.edu.cn; Tel.: +86-571-8795-5732

Catalogue

Typical NMR Spectra 2

Spectra of results listed in Table 1. 2

Spectra of results listed in Table 2. 20

Spectra of results listed in Table 3. 38

TGA curve of tri-block copolymer........ 46

Typical NMR Spectra

Typical NMR spectra are presented here to give a comprehensive information. Figures S1-S12 are the NMR spectra corresponded to entries 1-12 in Table 1 respectively. TEB and DTMeAB were used as Lewis pair in the presence of chain transfer agents (MPEG750 and PEG2000) with various feeding ratio. Figures S13-S24 reported the effects of different factors (entries 1-11 in Table 2). Figure S25-S30 are the results of block copolymers from several kinds of Lewis pairs.

Spectra of results listed in Table 1.

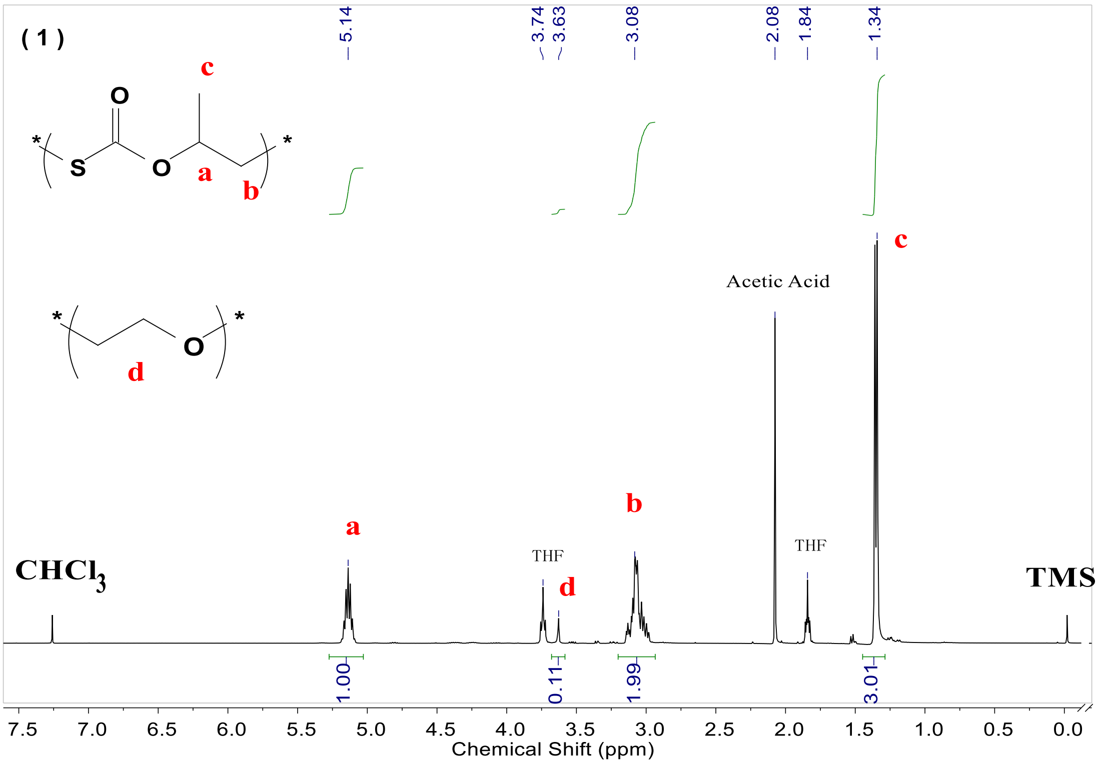

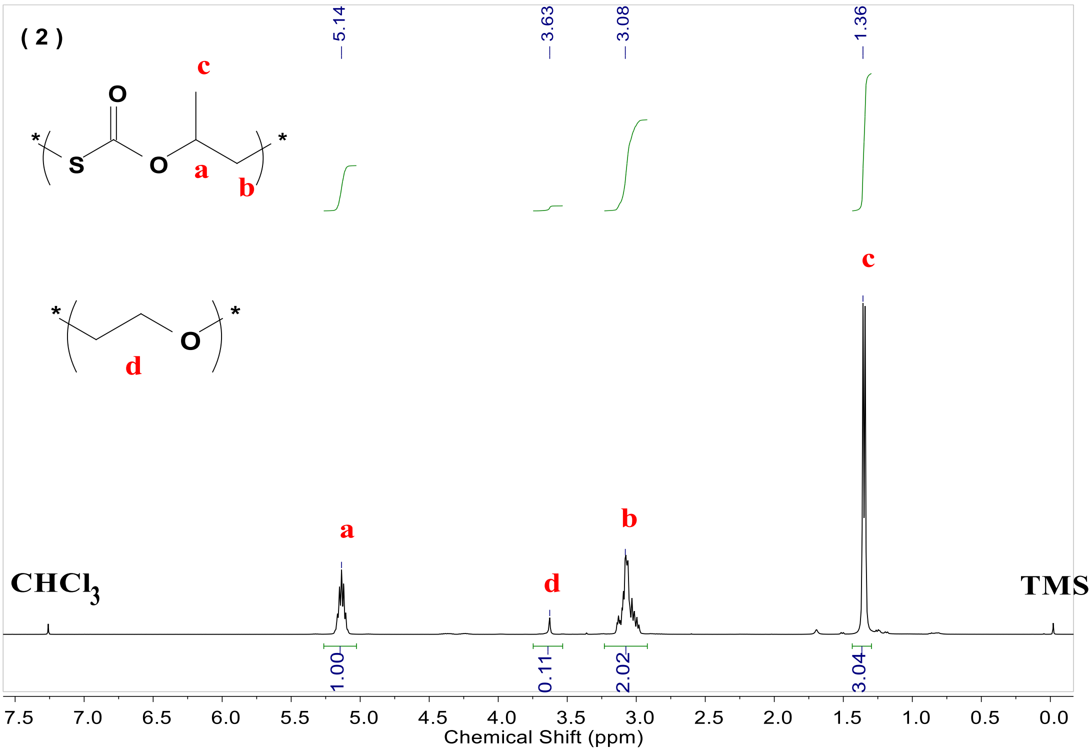

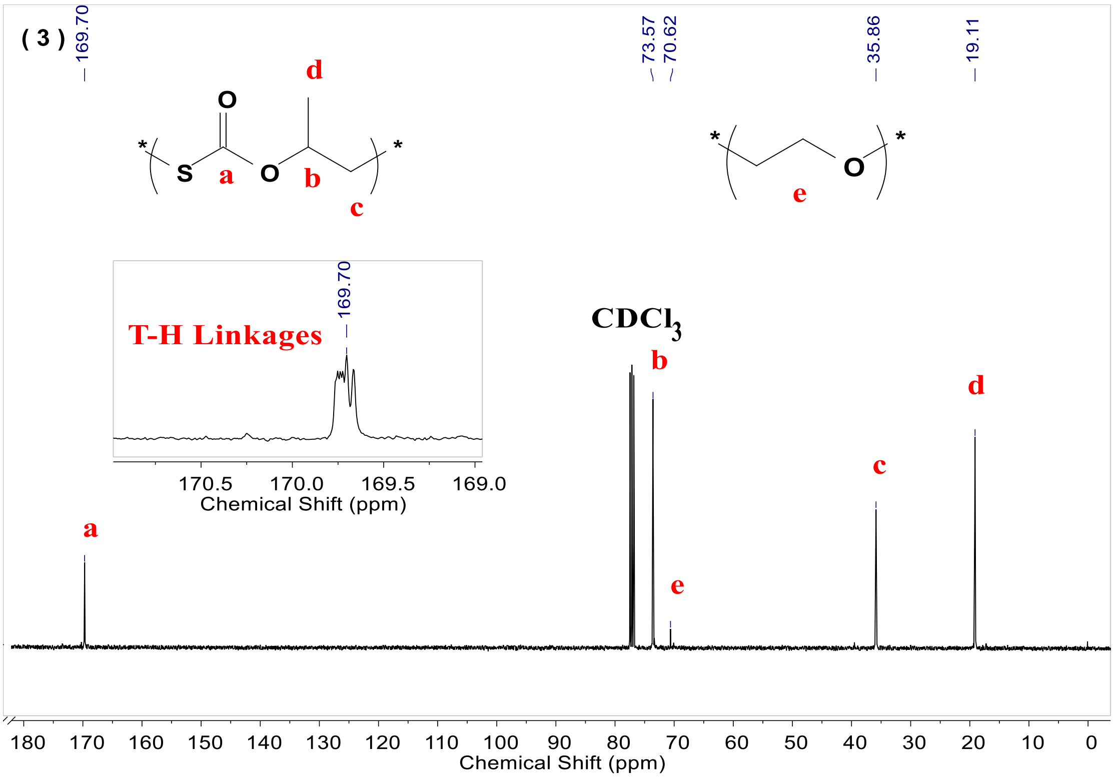

Figure S1. (1)^{1}H NMR spectrum of the crude product of entry 1, Table 1; (2) ^{1}H NMR spectrum of the purified product of entry 1, Table 1; (3) ^{13}C NMR spectrum of the purified product of entry 1, Table 1.

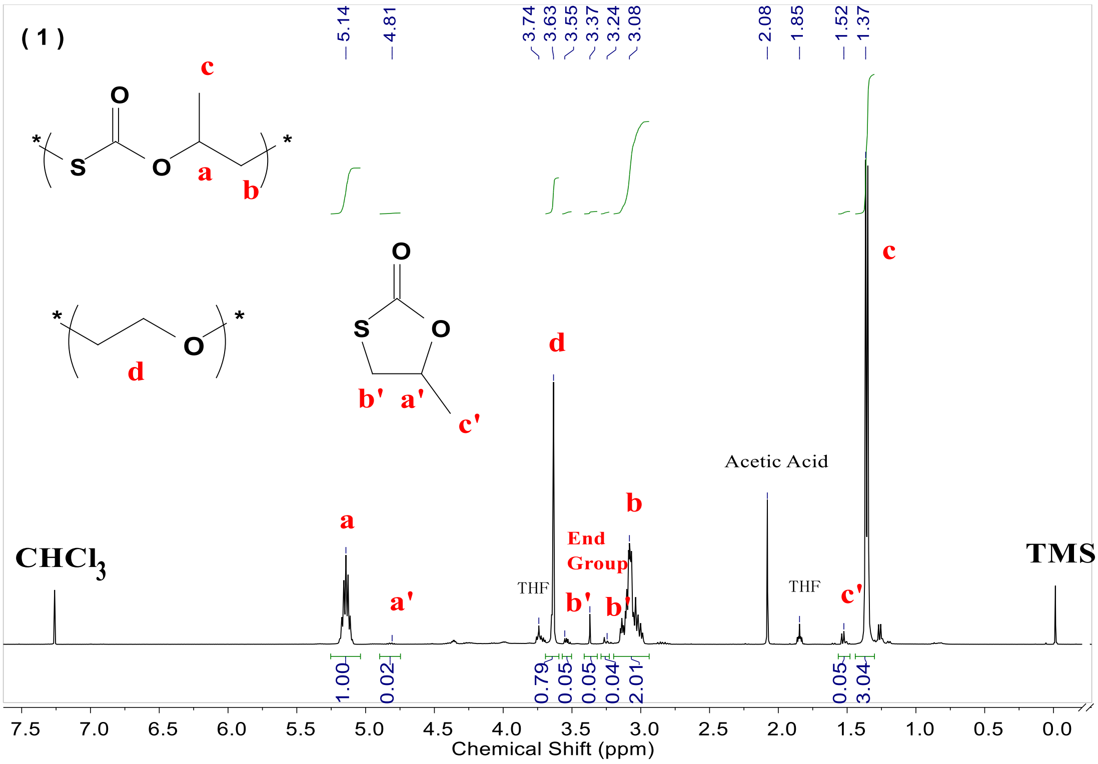
(1)
5.14
4.81
3.74
3.63
3.55
3.37
3.24
3.08
2.08
1.85
1.52
1.37
c
a
b
d
b' a'
c'
Acetic Acid
CHCl3
TMS
THF
End Group
a'
b'
b'
c'
1.00
0.02
0.79
0.05
0.05
0.04
2.01
0.05
3.04
Chemical Shift (ppm)

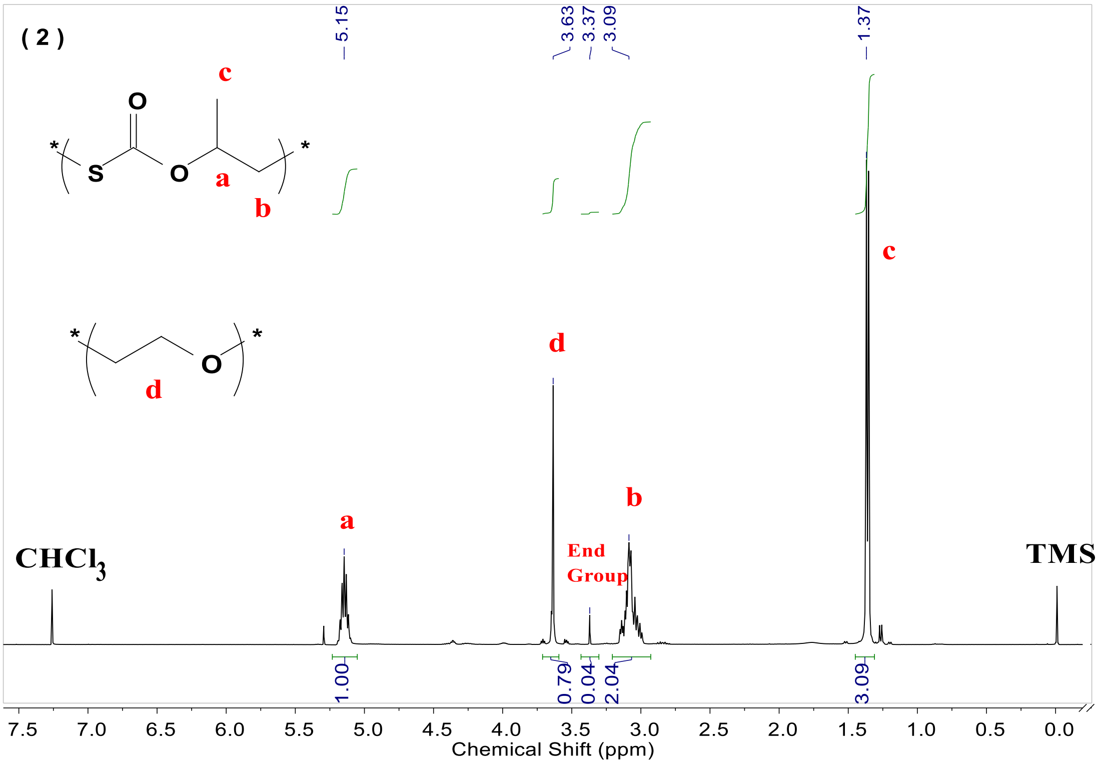
(2)
5.15
3.63
3.37
3.09
1.37
c
a
b
d
CHCl3
TMS
End Group
1.00
0.79
0.04
2.04
3.09
Chemical Shift (ppm)

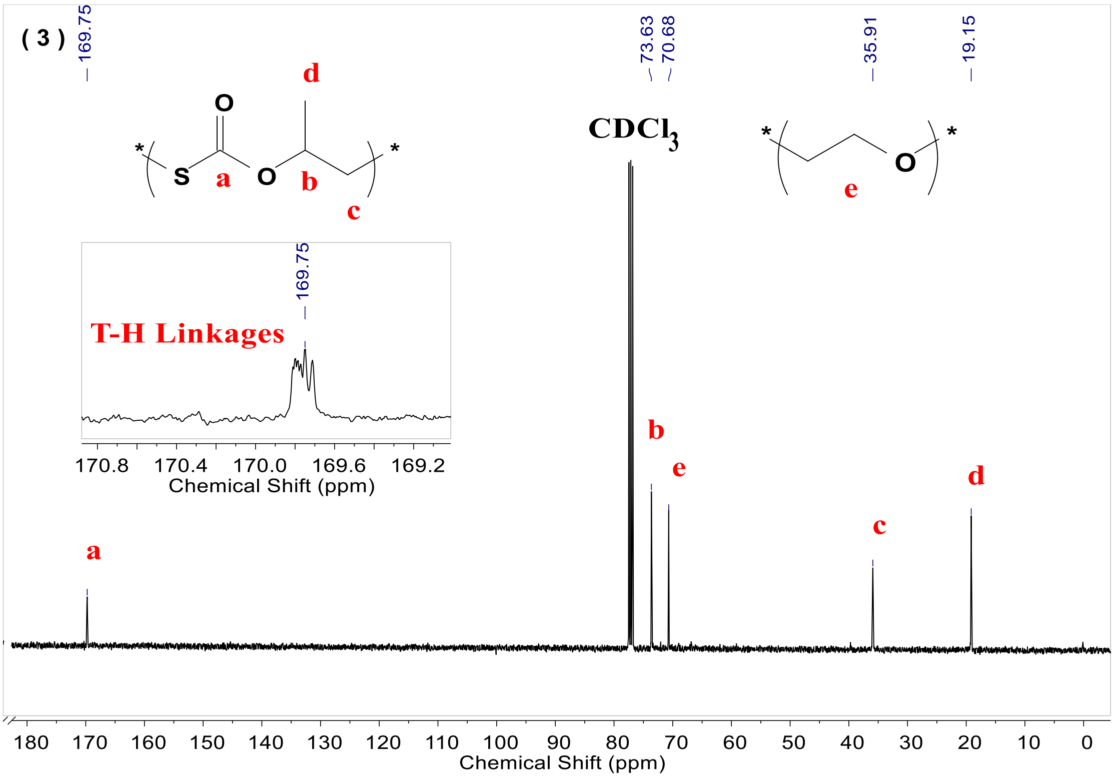

Figure S2. (1)^{1}H NMR spectrum of the crude product of entry 2, Table 1; (2) ^{1}H NMR spectrum of the purified product of entry 2, Table 1; (3) ^{13}C NMR spectrum of the purified product of entry 2, Table 1.

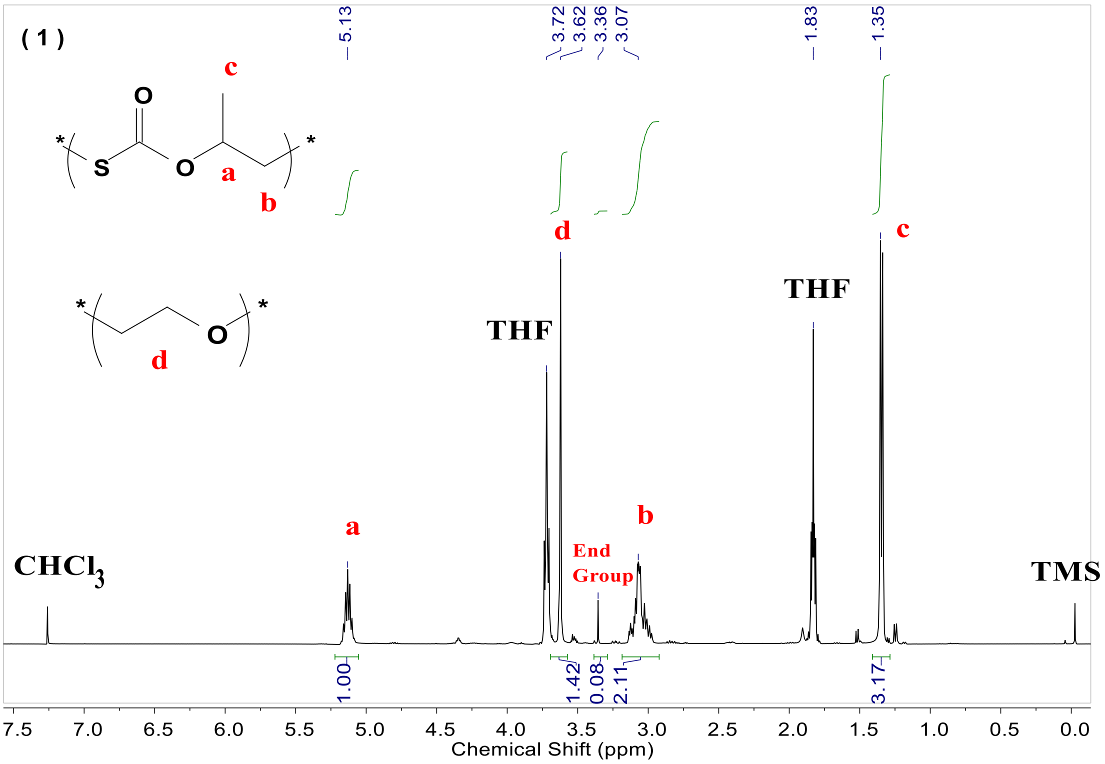

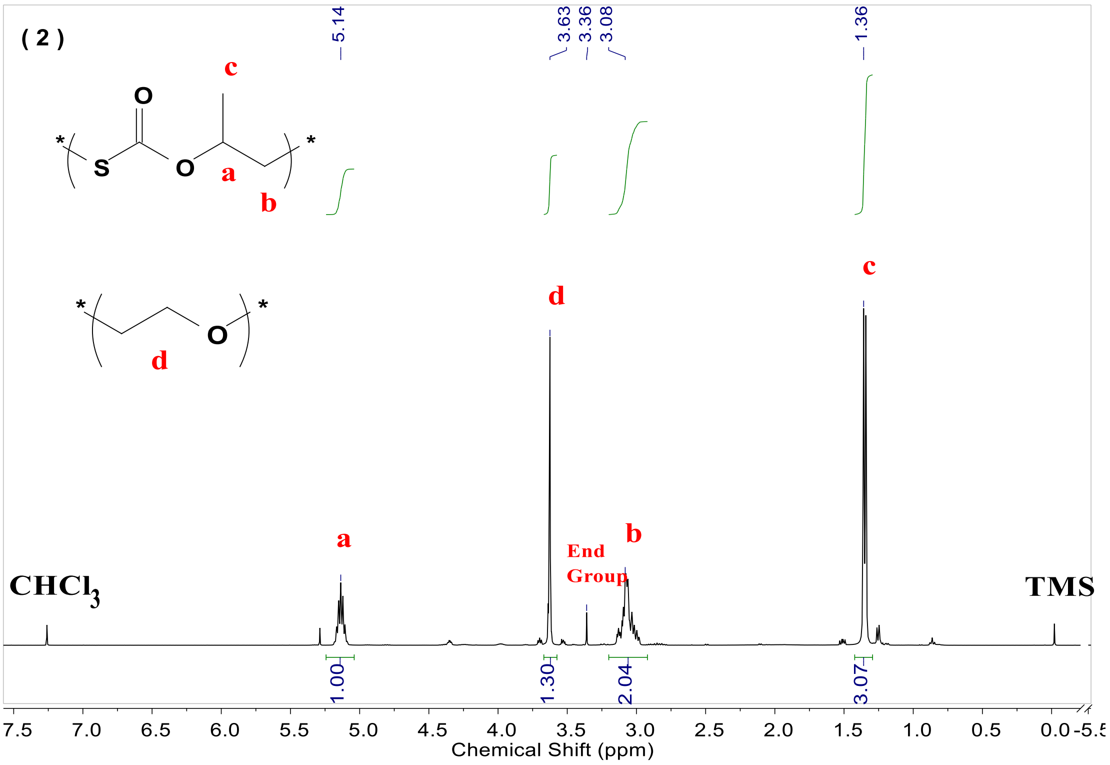

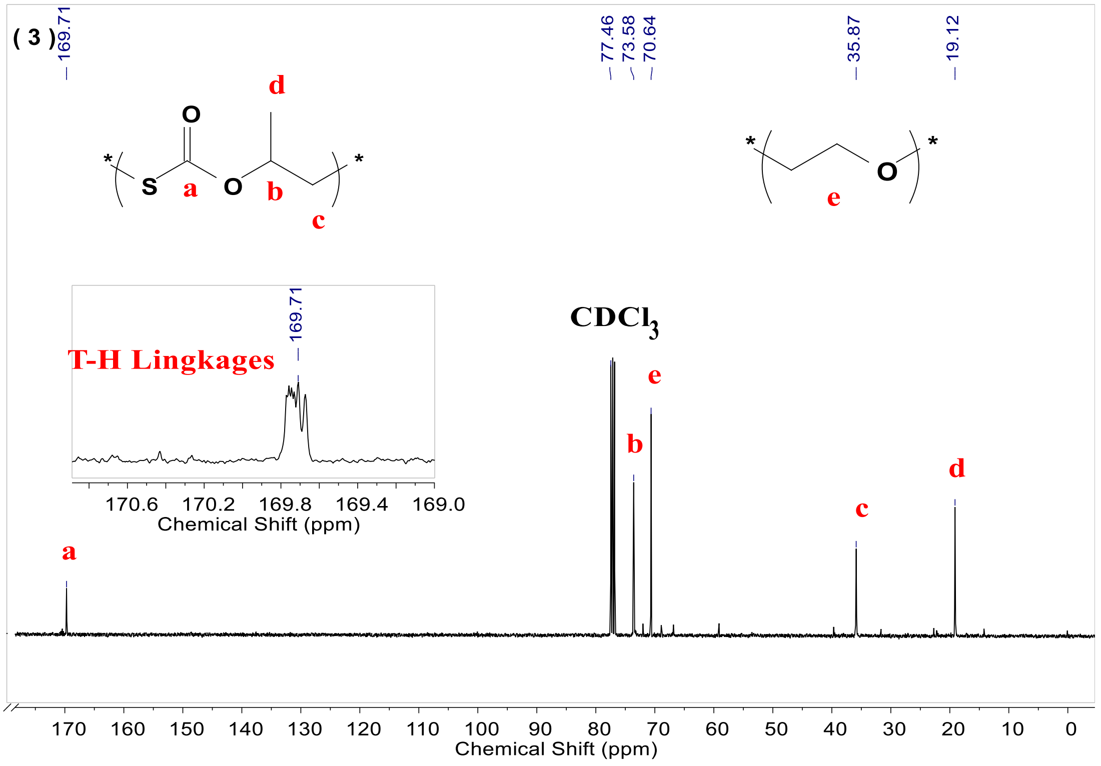

Figure S3. (1)^{1}H NMR spectrum of the crude product of entry 3, Table 1; (2) ^{1}H NMR spectrum of the purified product of entry 3, Table 1; (3) ^{13}C NMR spectrum of the purified product of entry 3, Table 1.

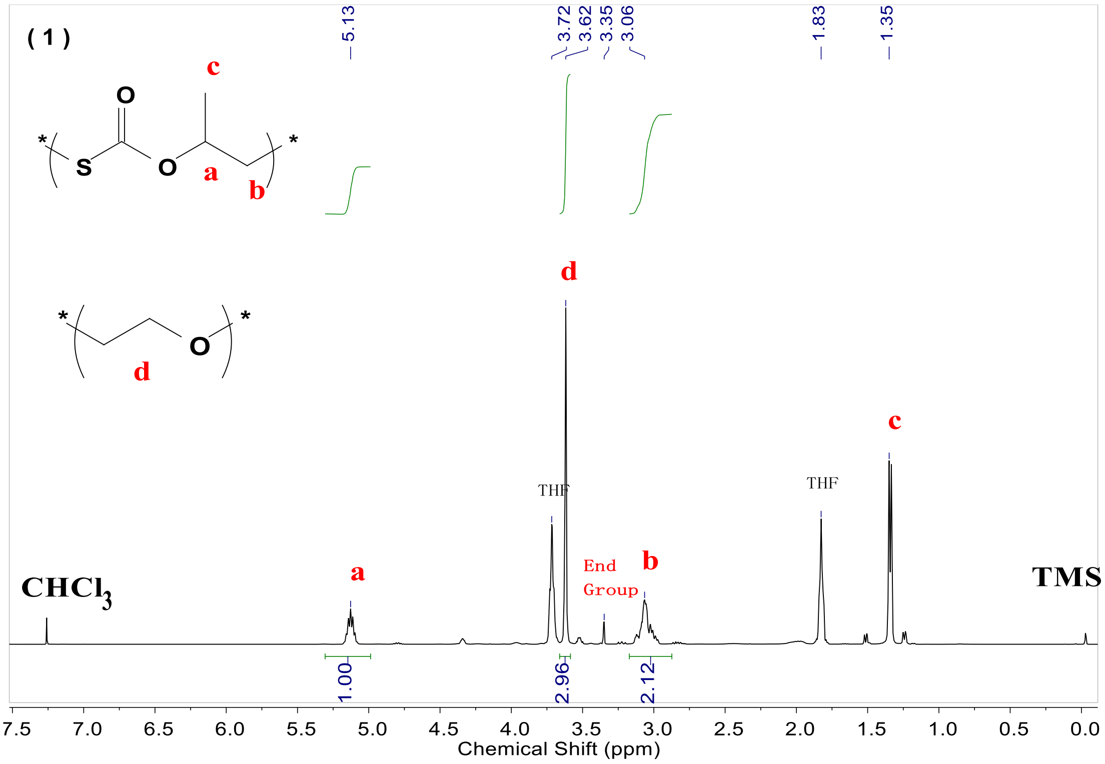

(1)
5.13
3.72
3.62
3.35
3.06
1.83
1.35
O
S
O
a
b
c
d
O
d
THF
THF
c
CHCl3
a
End
Group
b
TMS
1.00
2.96
2.12
7.5
7.0
6.5
6.0
5.5
5.0
4.5
4.0
3.5
3.0
2.5
2.0
1.5
1.0
0.5
0.0
Chemical Shift (ppm)

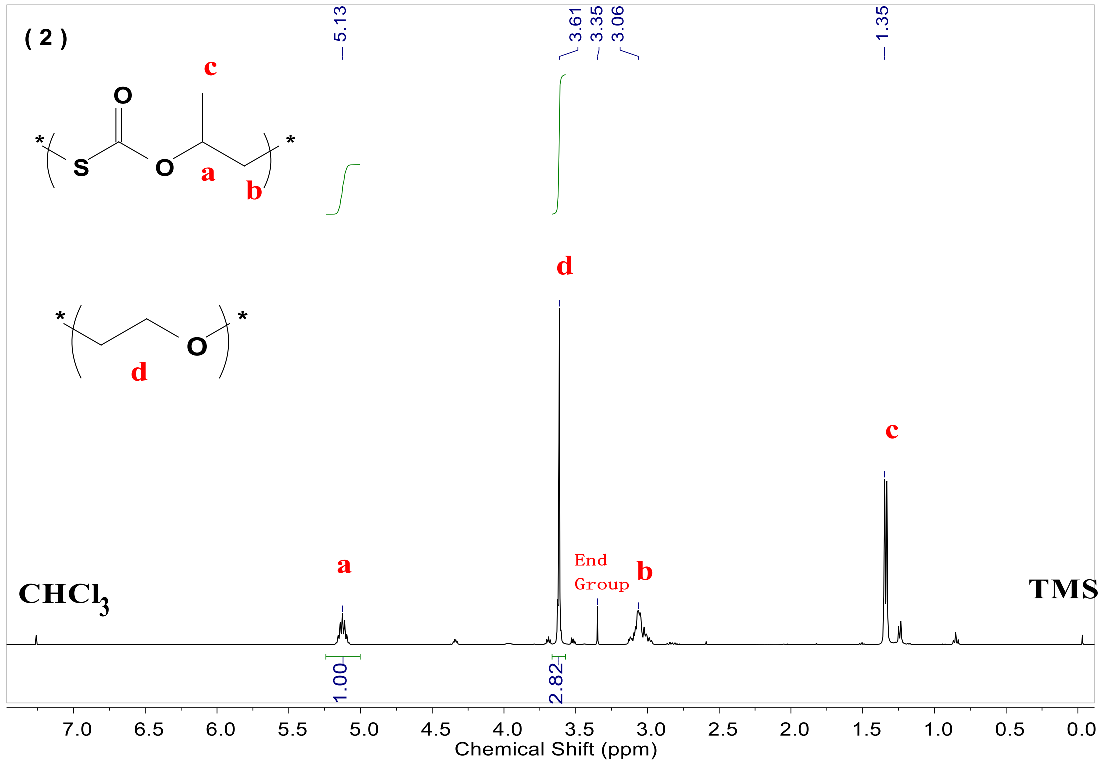

(2)
5.13
3.61
3.35
3.06
1.35
O
S
O
a
b
c
d
O
d
c
CHCl3
a
End
Group
b
TMS
1.00
2.82
7.0
6.5
6.0
5.5
5.0
4.5
4.0
3.5
3.0
2.5
2.0
1.5
1.0
0.5
0.0
Chemical Shift (ppm)

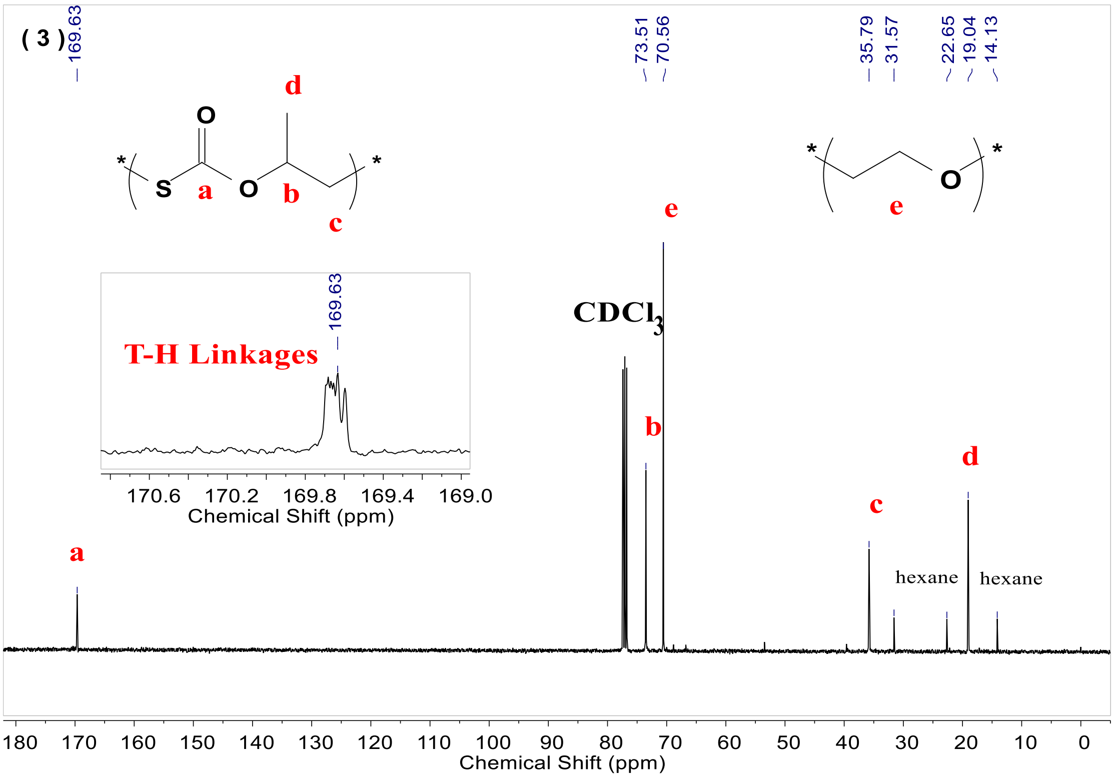

Figure S4. (1)^{1}H NMR spectrum of the crude product of entry 4, Table 1; (2) ^{1}H NMR spectrum of the purified product of entry 4, Table 1; (3) ^{13}C NMR spectrum of the purified product of entry 4, Table 1.

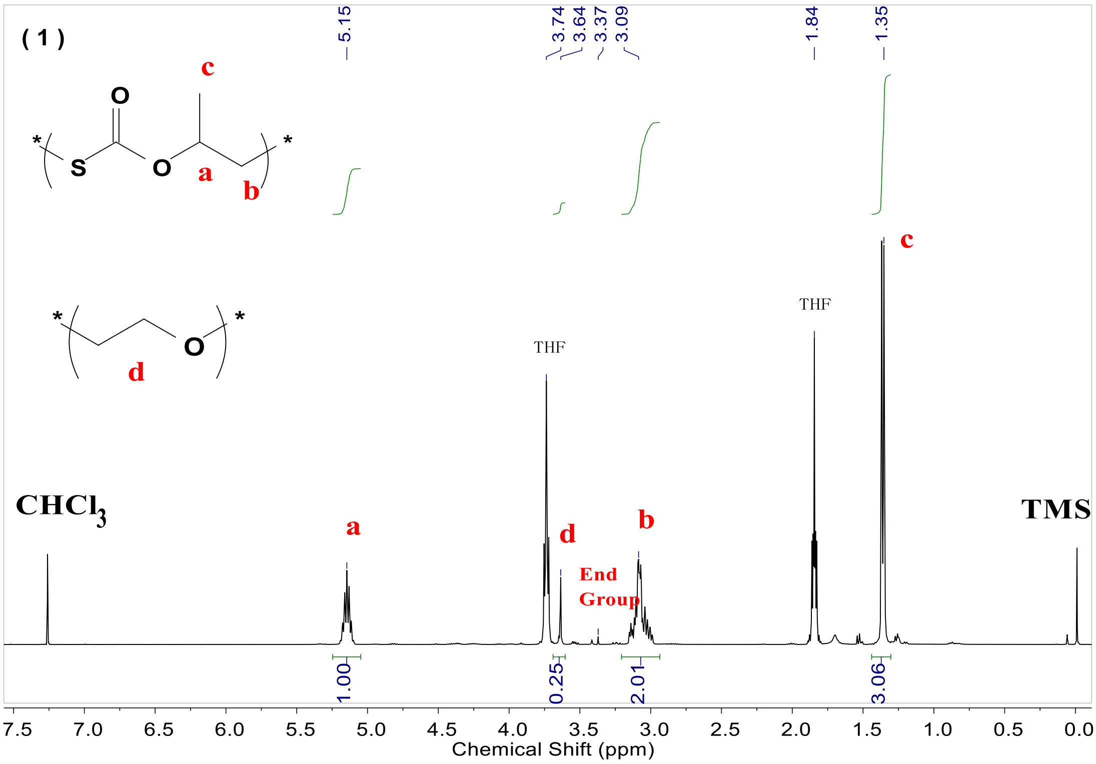

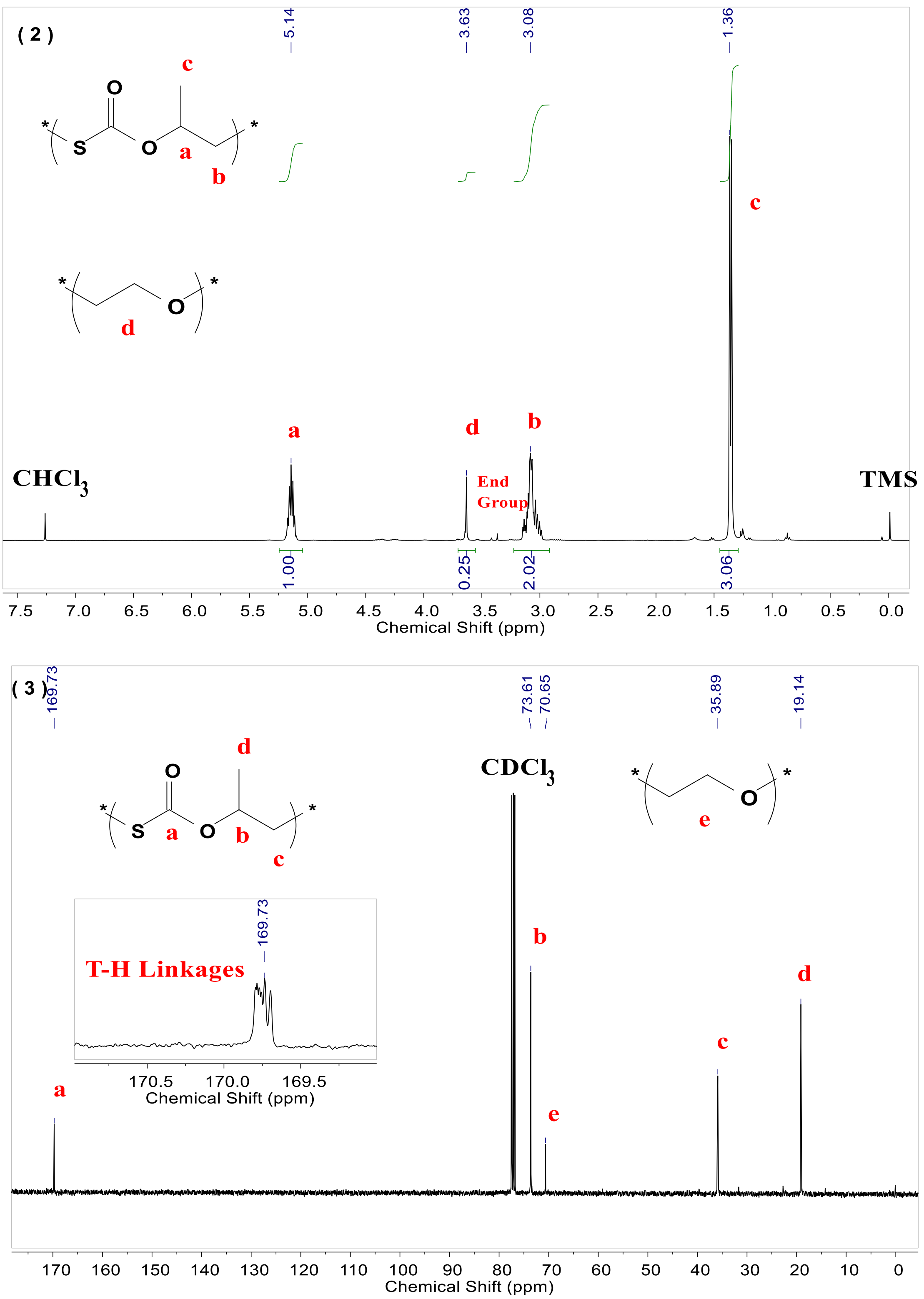

Figure S5. (1)^{1}H NMR spectrum of the crude product of entry 5, Table 1; (2) ^{1}H NMR spectrum of the purified product of entry 5, Table 1; (3) ^{13}C NMR spectrum of the purified product of entry 5, Table 1.

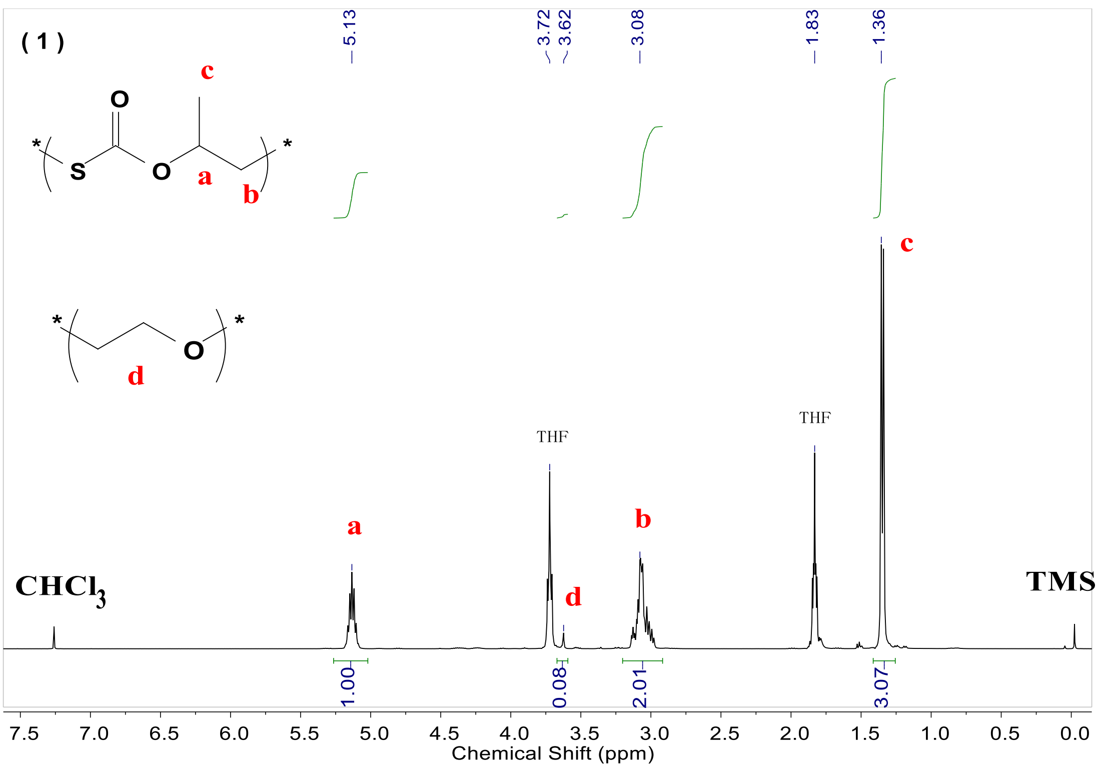

(1)
5.13
3.72
3.62
3.08
1.83
1.36
c
O
S
O
a
b
d
O
THF
THF
a
b
c
d
CHCl3
TMS
1.00
0.08
2.01
3.07
7.5 7.0 6.5 6.0 5.5 5.0 4.5 4.0 3.5 3.0 2.5 2.0 1.5 1.0 0.5 0.0
Chemical Shift (ppm)

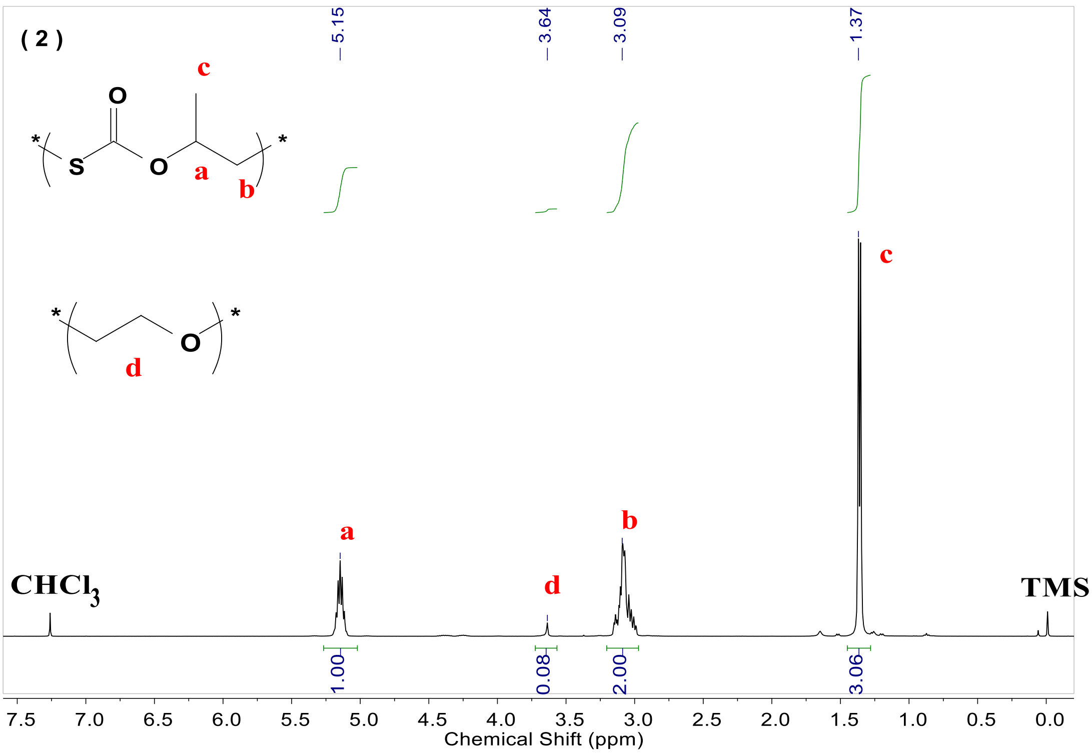

(2)
5.15
3.64
3.09
1.37
c
O
S
O
a
b
d
O
a
b
c
d
CHCl3
TMS
1.00
0.08
2.00
3.06
7.5 7.0 6.5 6.0 5.5 5.0 4.5 4.0 3.5 3.0 2.5 2.0 1.5 1.0 0.5 0.0
Chemical Shift (ppm)

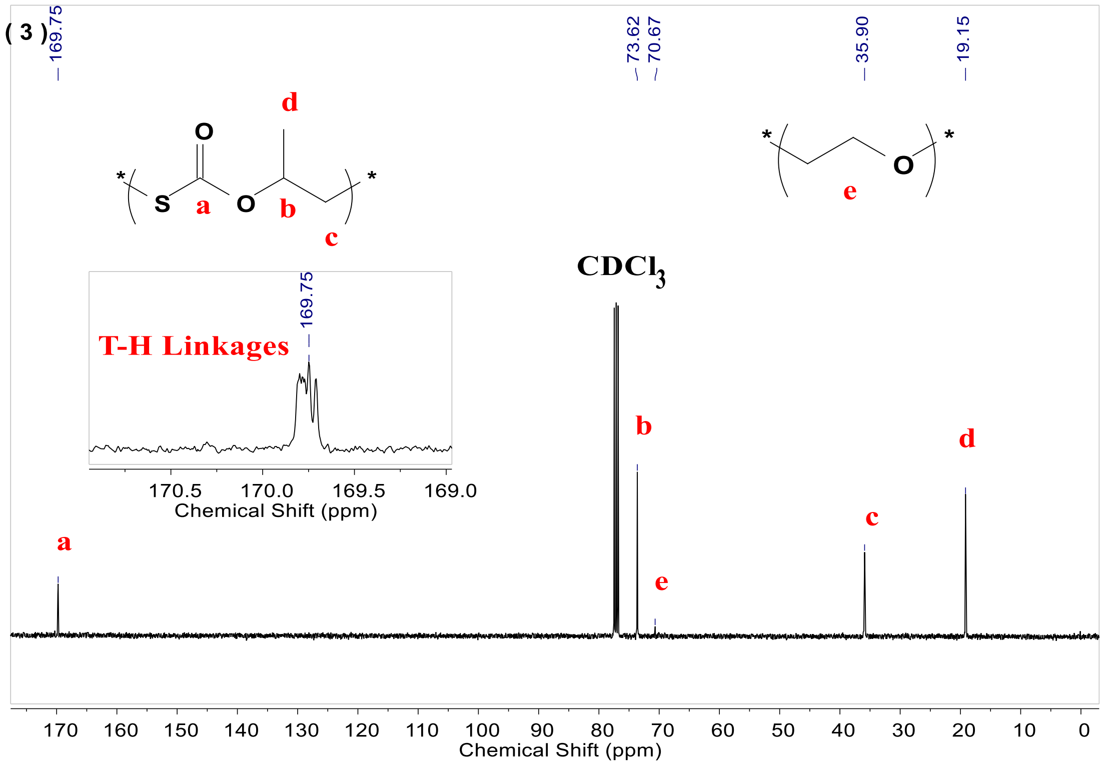

Figure S6. (1)^{1}H NMR spectrum of the crude product of entry 6, Table 1; (2) ^{1}H NMR spectrum of the purified product of entry 6, Table 1; (3) ^{13}C NMR spectrum of the purified product of entry 6, Table 1.

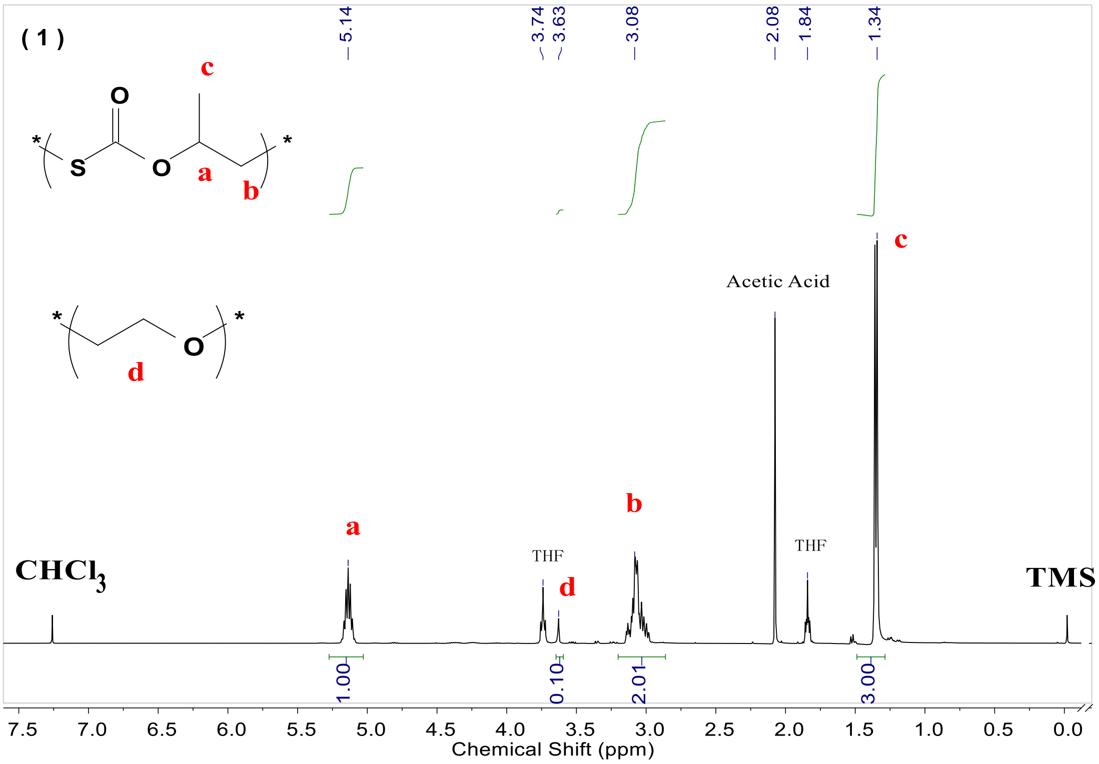

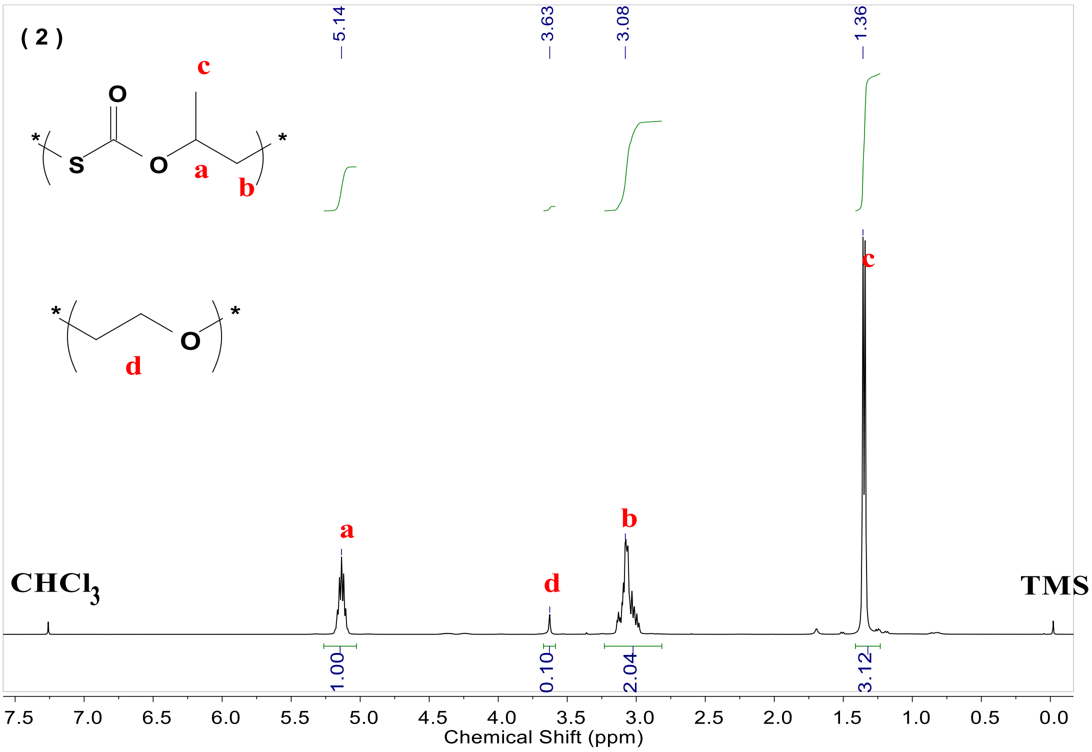

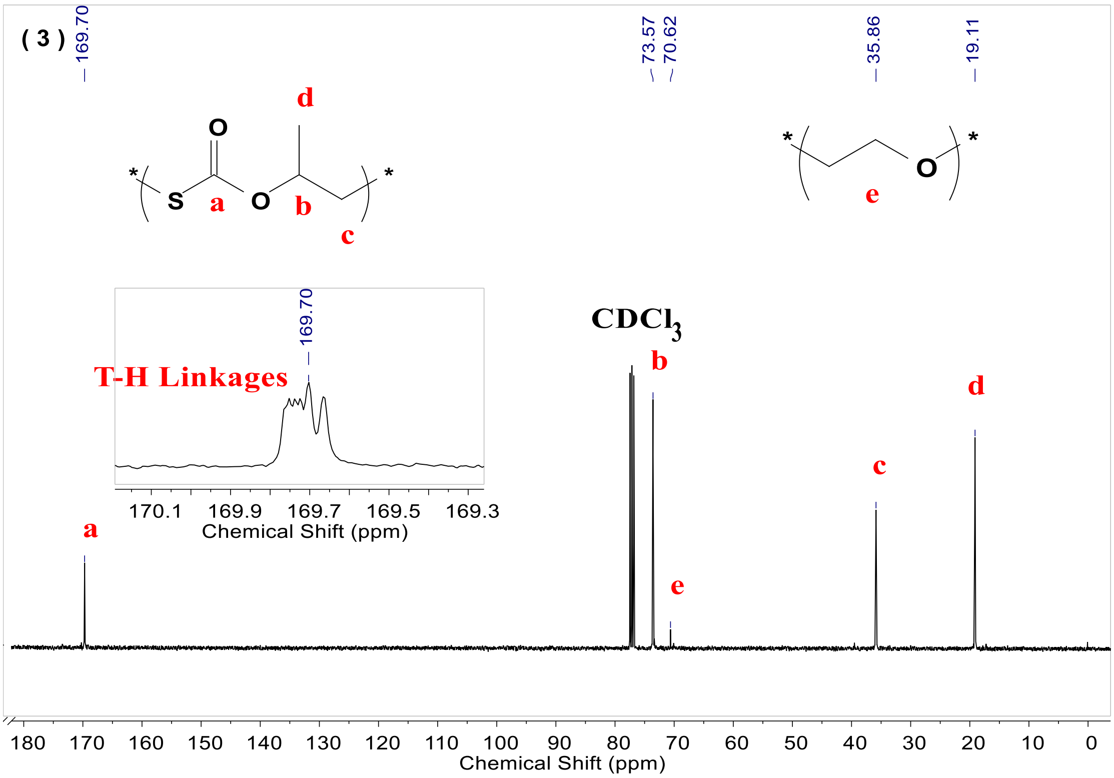

Figure S7. (1)^{1}H NMR spectrum of the crude product of entry 7, Table 1; (2) ^{1}H NMR spectrum of the purified product of entry 7, Table 1; (3) ^{13}C NMR spectrum of the purified product of entry 7, Table 1.

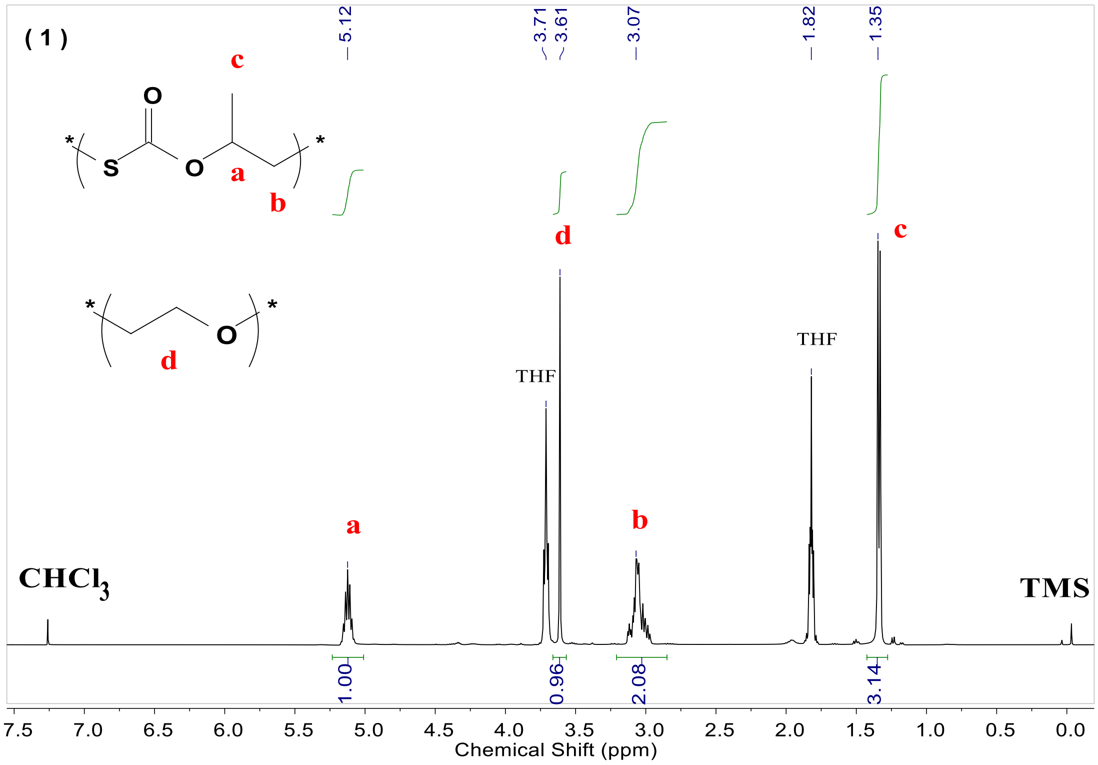

(1)
5.12
3.71
3.61
3.07
1.82
1.35
c
a
b
d
THF
THF
CHCl3
TMS
1.00
0.96
2.08
3.14
7.5 7.0 6.5 6.0 5.5 5.0 4.5 4.0 3.5 3.0 2.5 2.0 1.5 1.0 0.5 0.0
Chemical Shift (ppm)

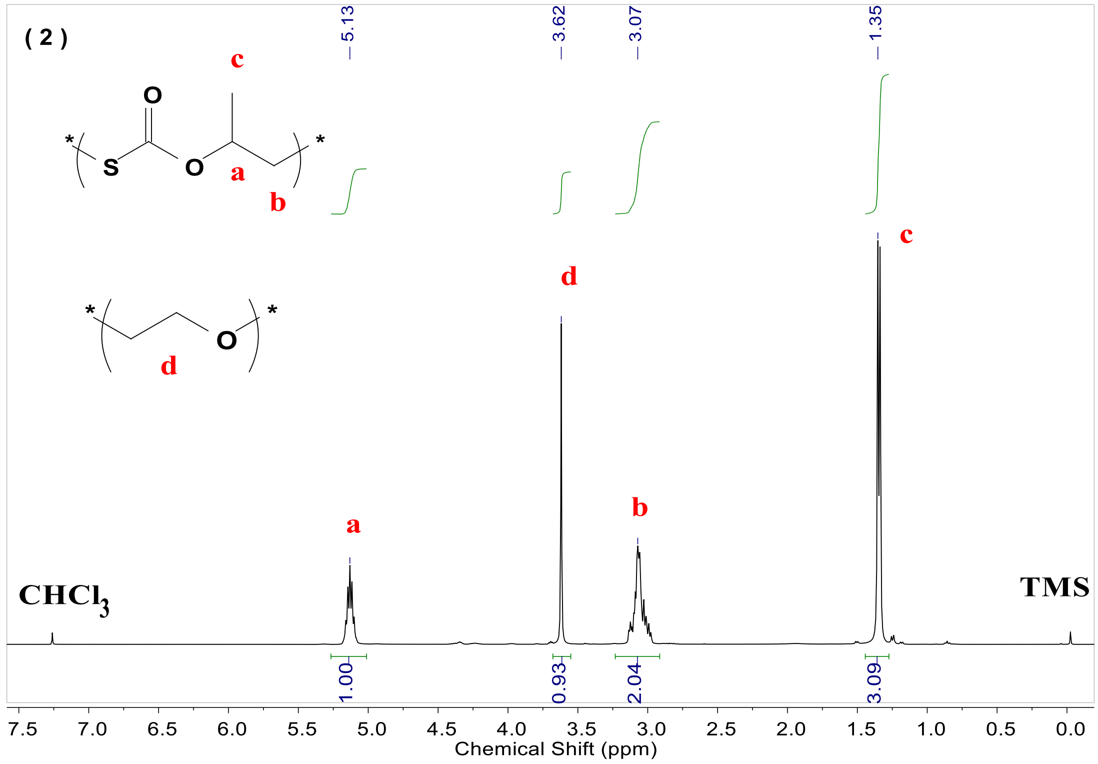

(2)
5.13
3.62
3.07
1.35
c
a
b
d
CHCl3
TMS
1.00
0.93
2.04
3.09
7.5 7.0 6.5 6.0 5.5 5.0 4.5 4.0 3.5 3.0 2.5 2.0 1.5 1.0 0.5 0.0
Chemical Shift (ppm)

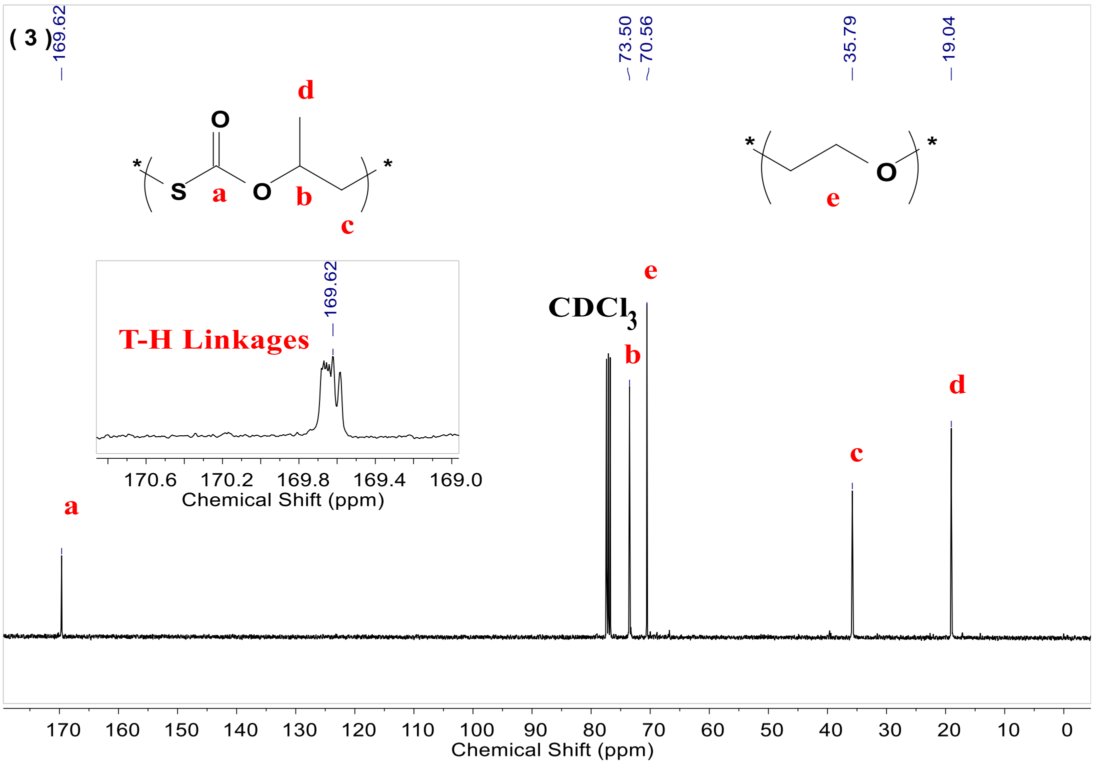

Figure S8. (1)^{1}H NMR spectrum of the crude product of entry 8, Table 1; (2) ^{1}H NMR spectrum of the purified product of entry 8, Table 1; (3) ^{13}C NMR spectrum of the purified product of entry 8, Table 1.

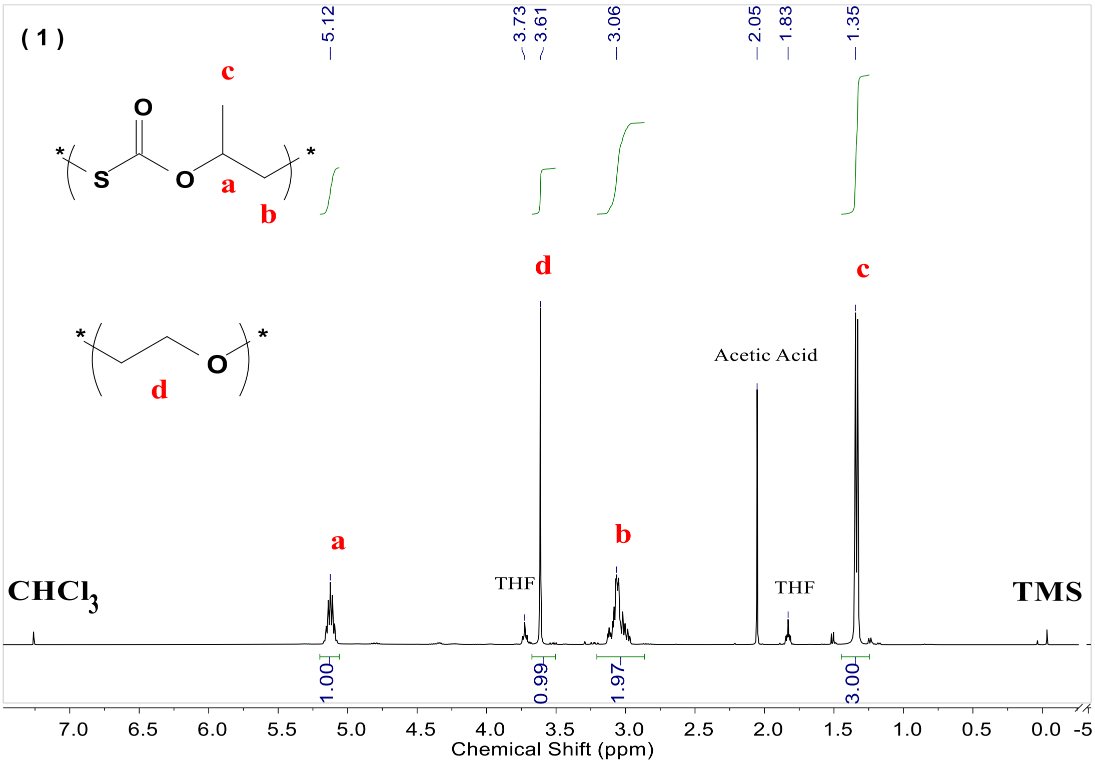

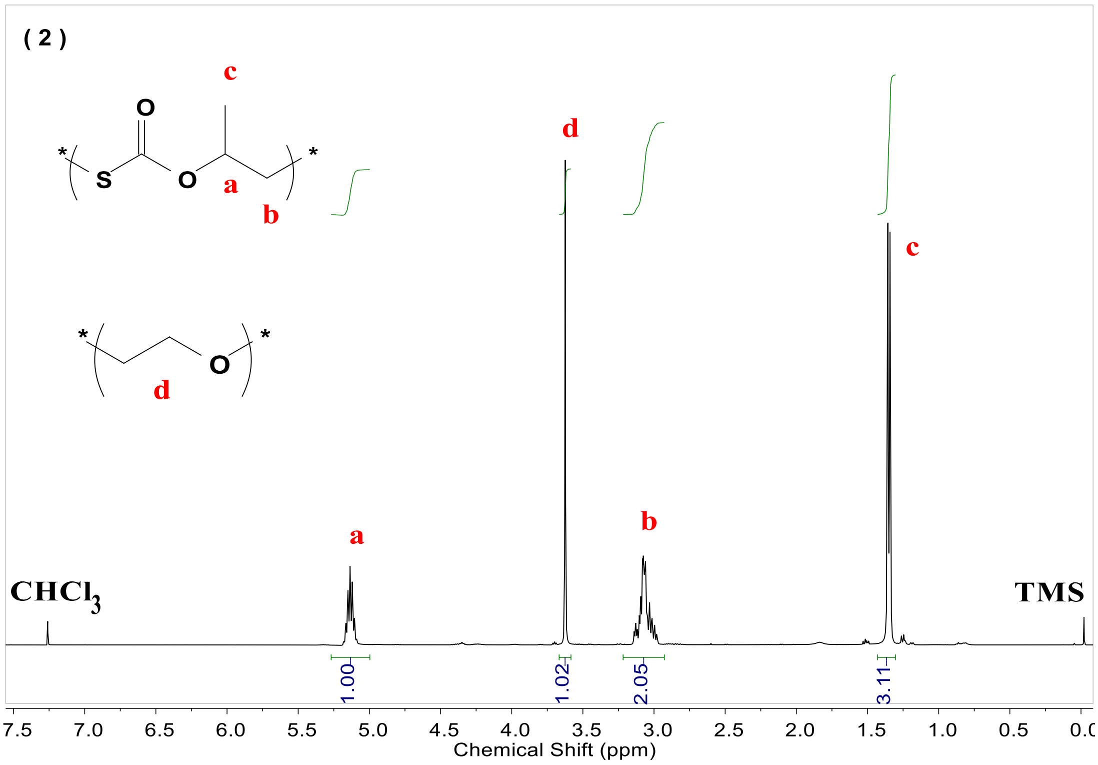

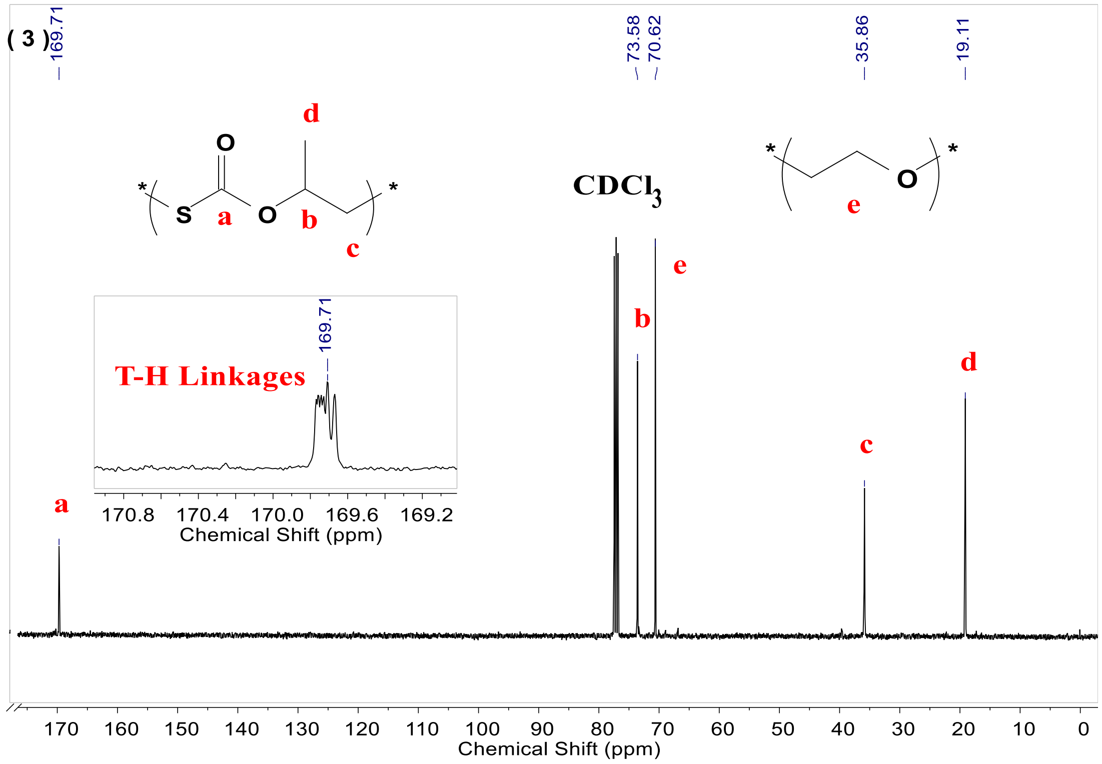

Figure S9. (1)^{1}H NMR spectrum of the crude product of entry 9, Table 1; (2) ^{1}H NMR spectrum of the purified product of entry 9, Table 1; (3) ^{13}C NMR spectrum of the purified product of entry 9, Table 1.

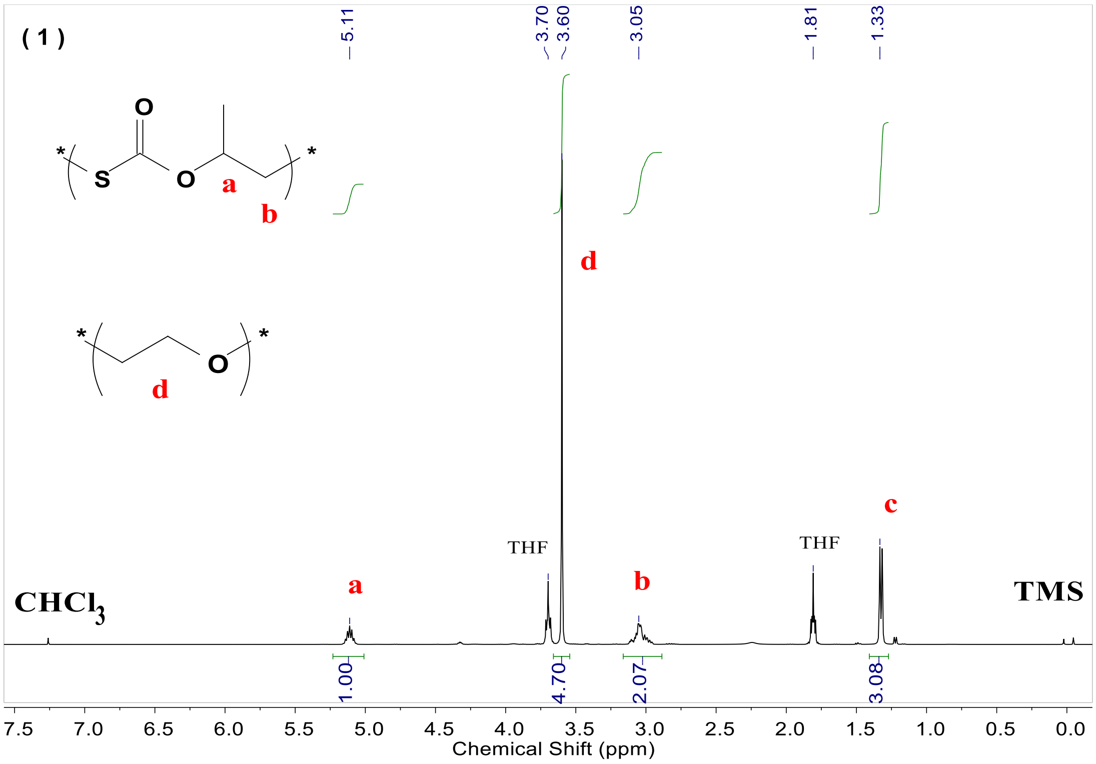

(1)
5.11
3.70
3.60
3.05
1.81
1.33
O
S
O
a
b
d
O
d
THF
THF
c
a
b
CHCl3
TMS
1.00
4.70
2.07
3.08
7.5 7.0 6.5 6.0 5.5 5.0 4.5 4.0 3.5 3.0 2.5 2.0 1.5 1.0 0.5 0.0
Chemical Shift (ppm)

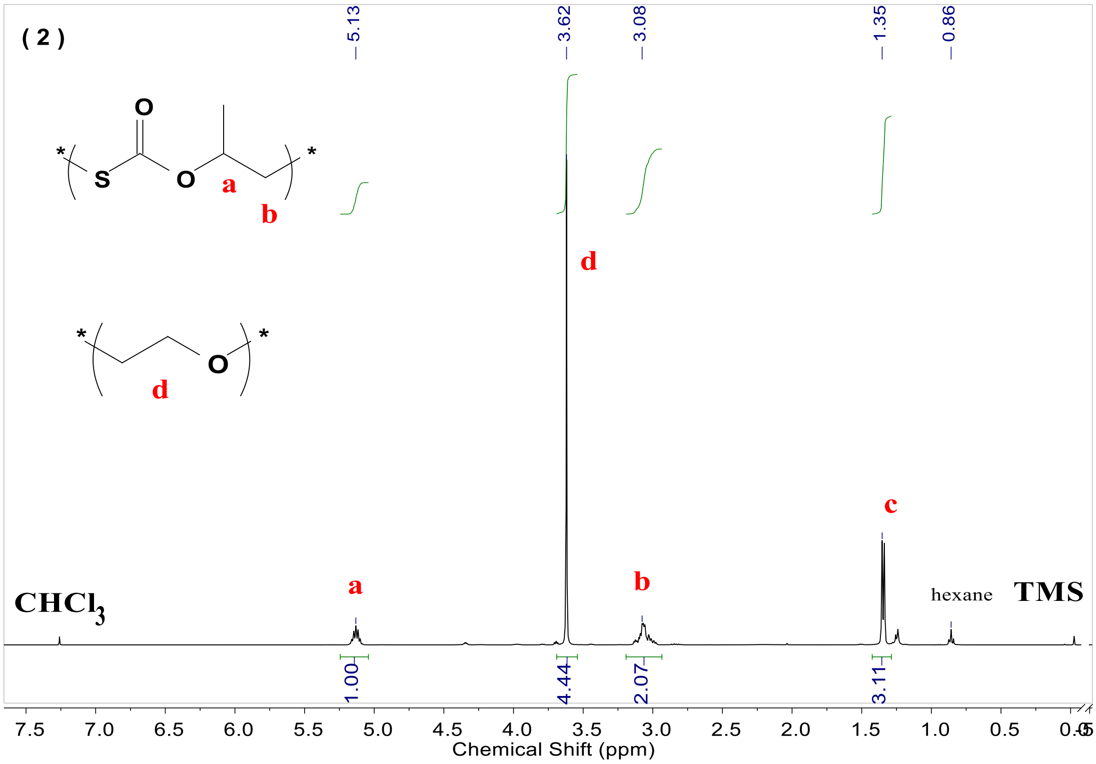

(2)
5.13
3.62
3.08
1.35
0.86
O
S
O
a
b
d
O
d
c
a
b
hexane
CHCl3
TMS
1.00
4.44
2.07
3.11
7.5 7.0 6.5 6.0 5.5 5.0 4.5 4.0 3.5 3.0 2.5 2.0 1.5 1.0 0.5
Chemical Shift (ppm)

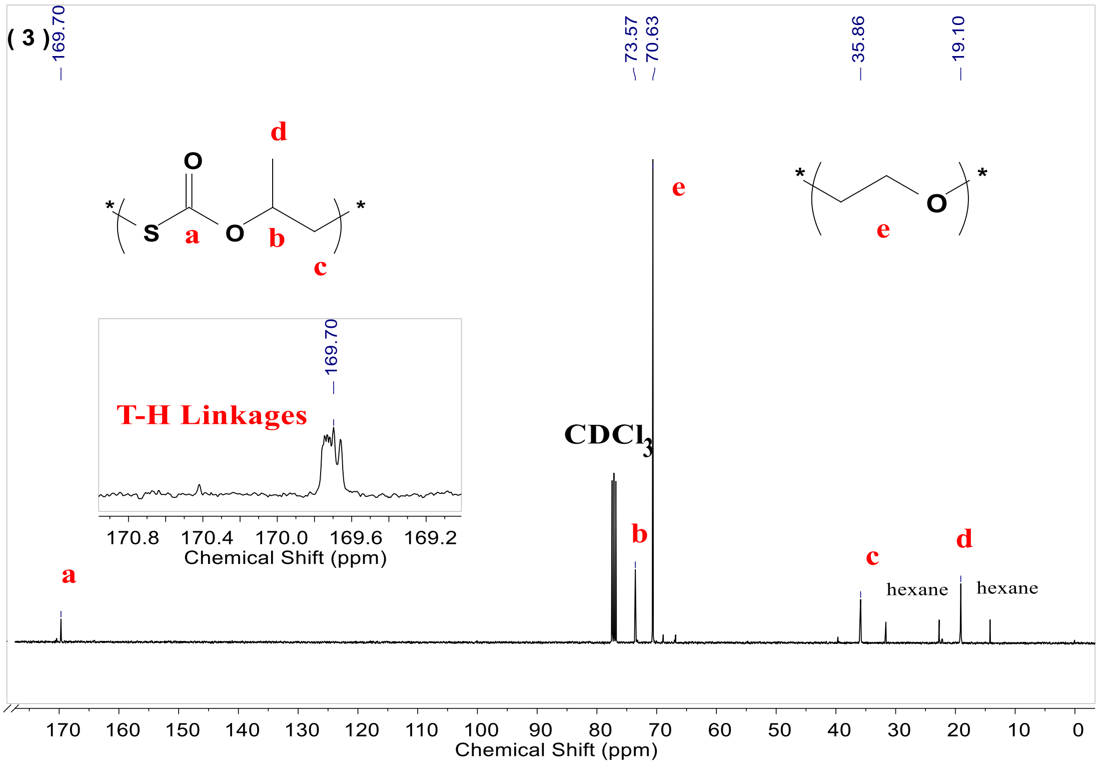

Figure S10. (1)^{1}H NMR spectrum of the crude product of entry 10, Table 1; (2) ^{1}H NMR spectrum of the purified product of entry 10, Table 1; (3) ^{13}C NMR spectrum of the purified product of entry 10, Table 1.

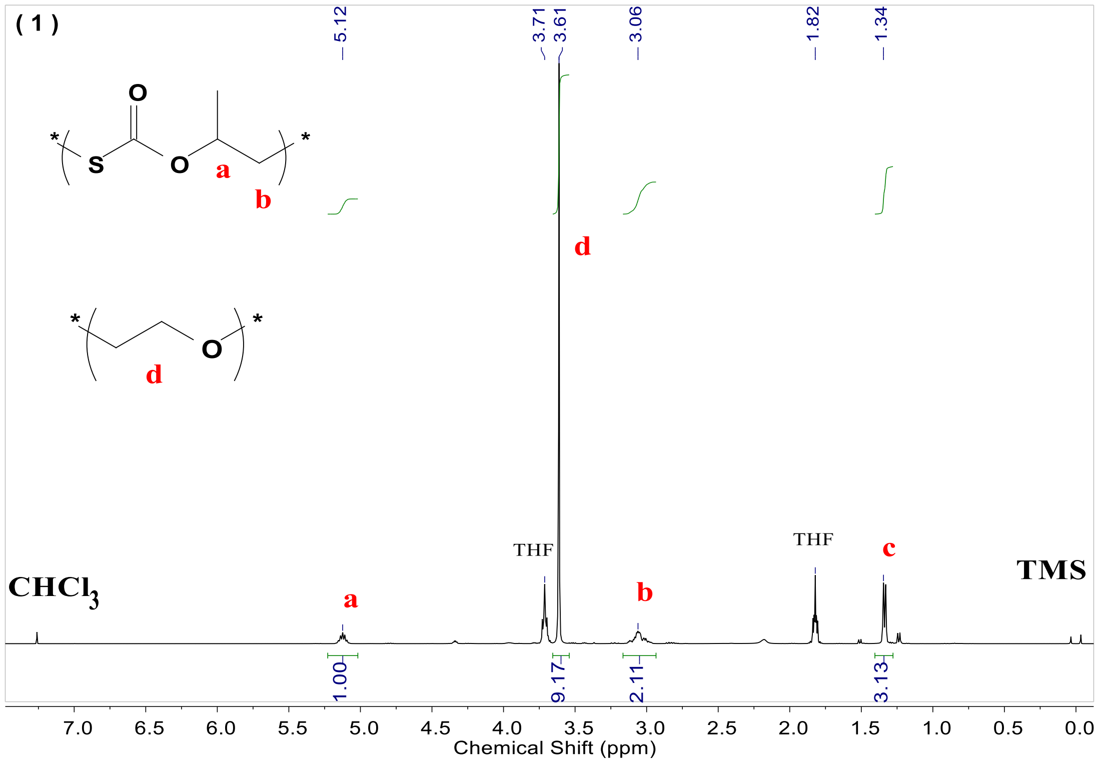

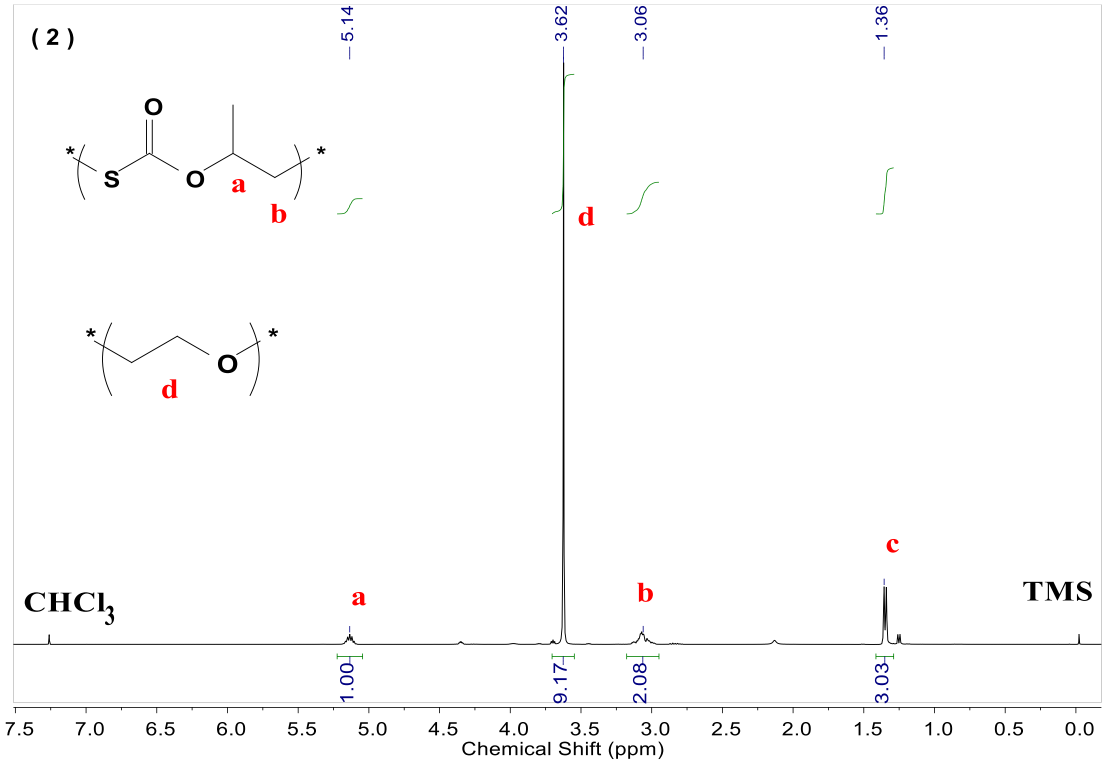

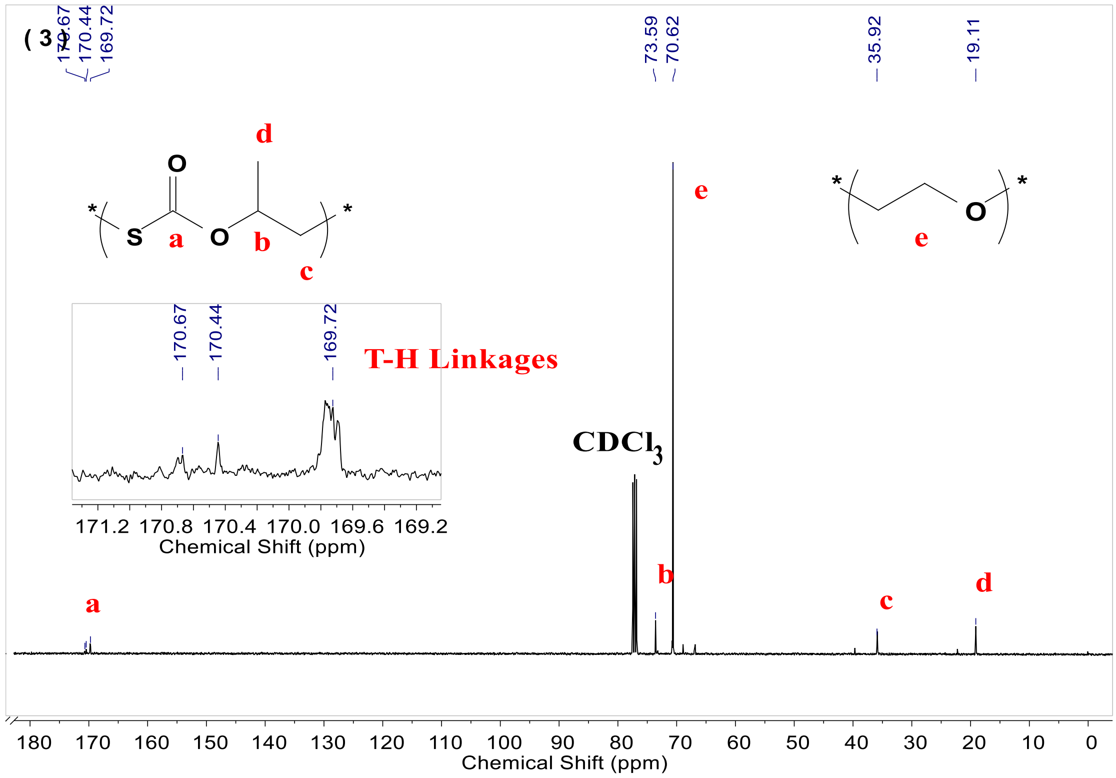

Figure S11. (1)^{1}H NMR spectrum of the crude product of entry 11, Table 1; (2) ^{1}H NMR spectrum of the purified product of entry 11, Table 1; (3) ^{13}C NMR spectrum of the purified product of entry 11, Table 1.

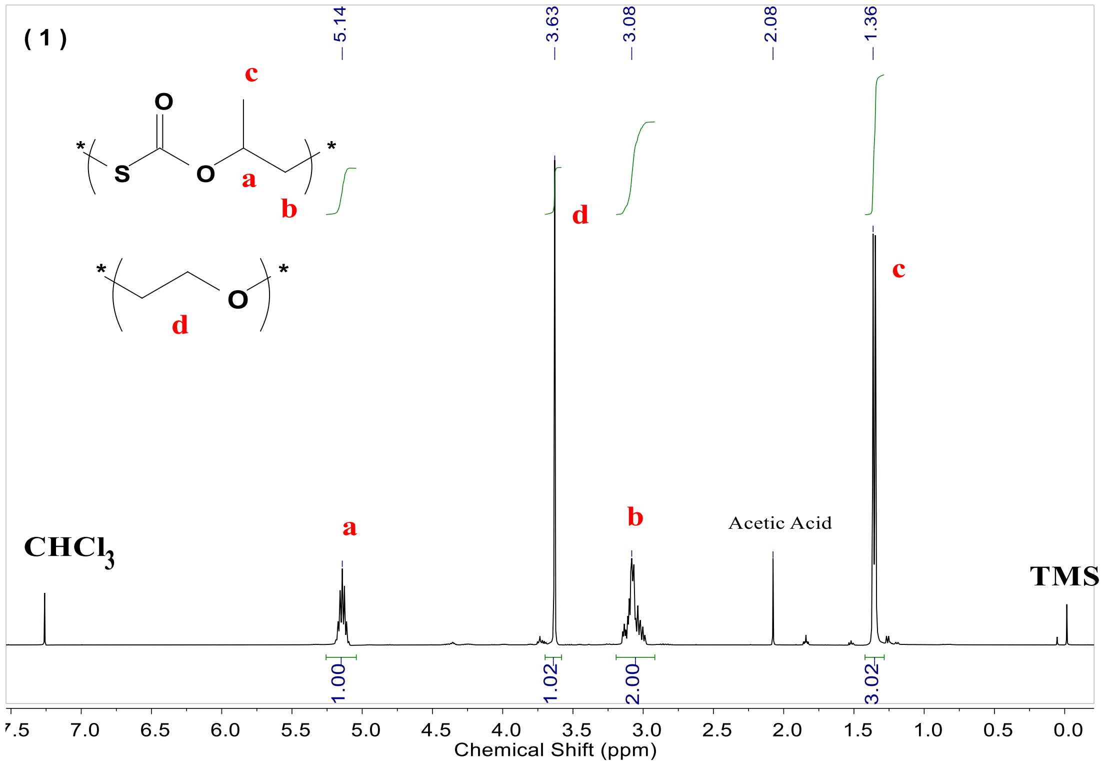
(1)
5.14
3.63
3.08
2.08
1.36
c
O
S
O
a
b
d
O
d
c
a
b
Acetic Acid
CHCl3
TMS
1.00
1.02
2.00
3.02
7.5 7.0 6.5 6.0 5.5 5.0 4.5 4.0 3.5 3.0 2.5 2.0 1.5 1.0 0.5 0.0
Chemical Shift (ppm)

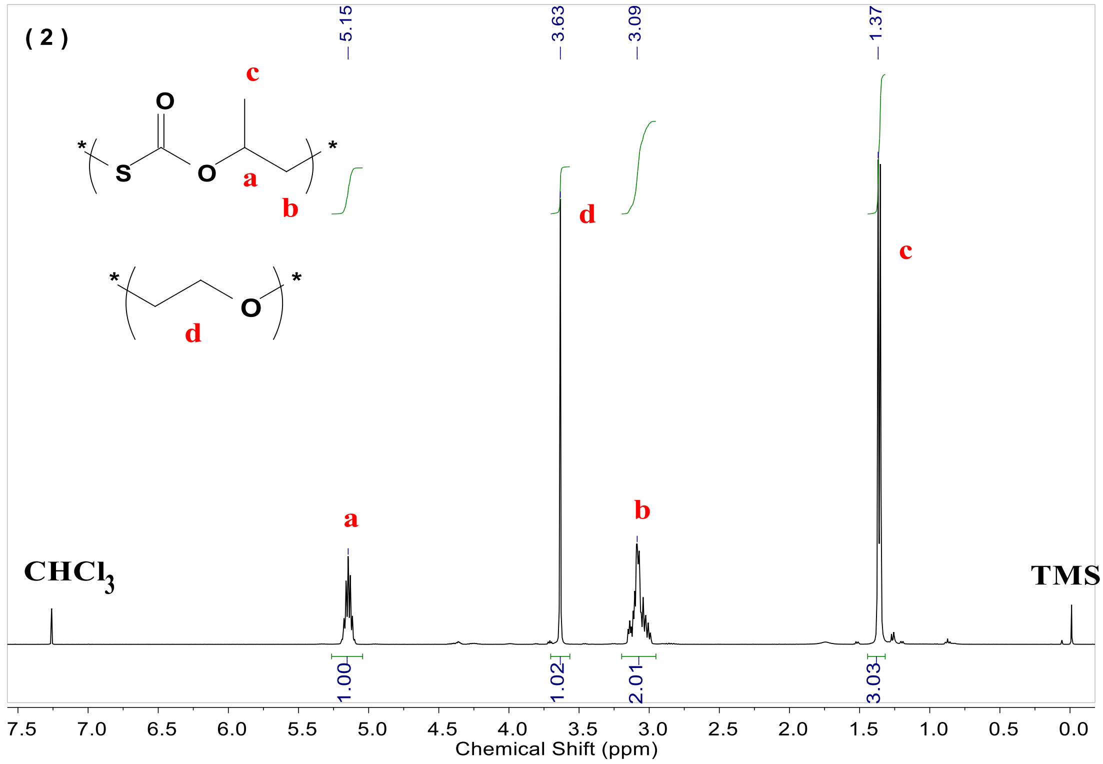
(2)
5.15
3.63
3.09
1.37
c
O
S
O
a
b
d
O
d
c
a
b
CHCl3
TMS
1.00
1.02
2.01
3.03
7.5 7.0 6.5 6.0 5.5 5.0 4.5 4.0 3.5 3.0 2.5 2.0 1.5 1.0 0.5 0.0
Chemical Shift (ppm)

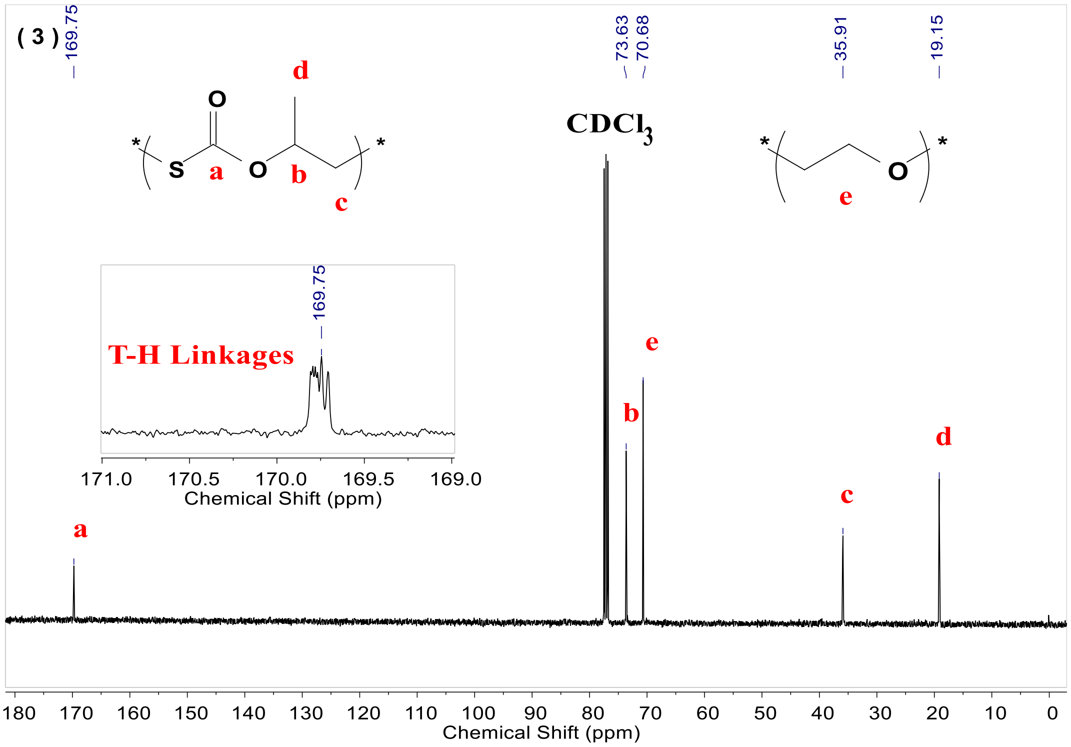

Figure S12. (1)^{1}H NMR spectrum of the crude product of entry 12, Table 1; (2) ^{1}H NMR spectrum of the purified product of entry 12, Table 1; (3) ^{13}C NMR spectrum of the purified product of entry 12, Table 1.

Spectra of results listed in Table 2.

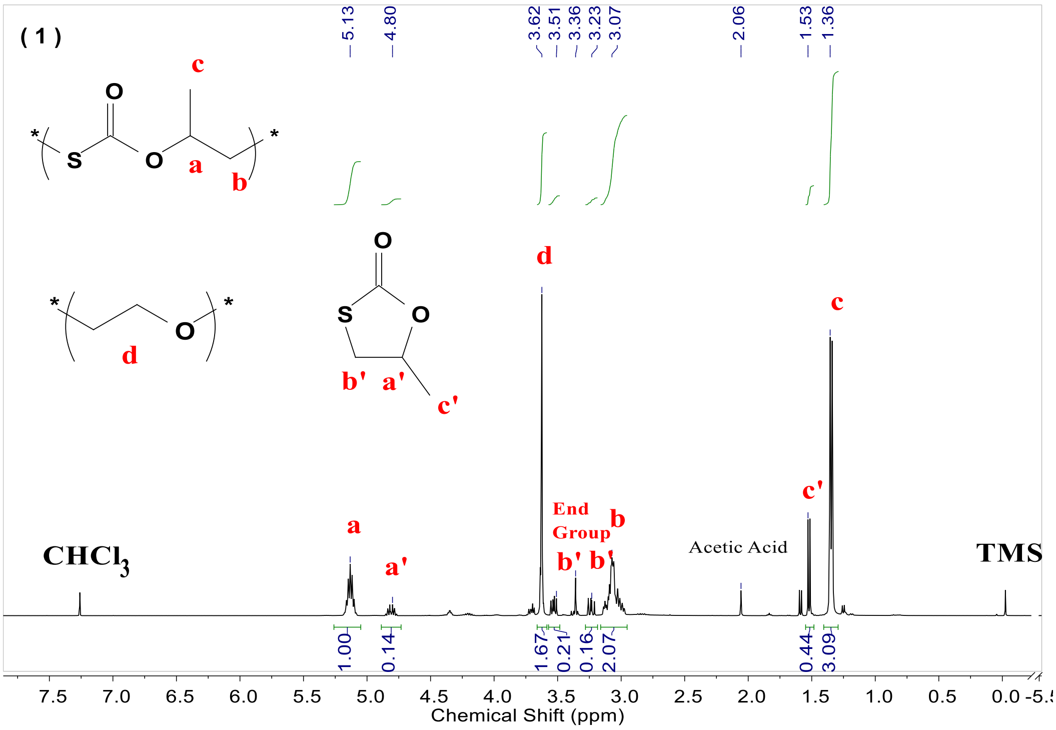

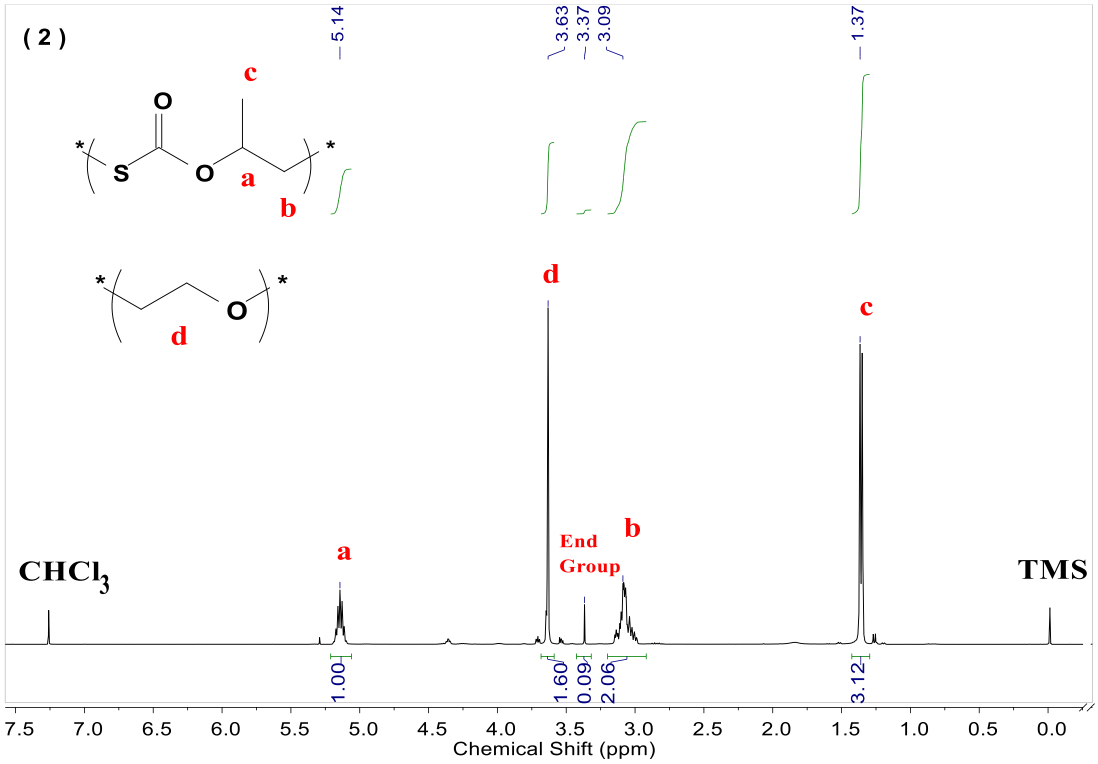

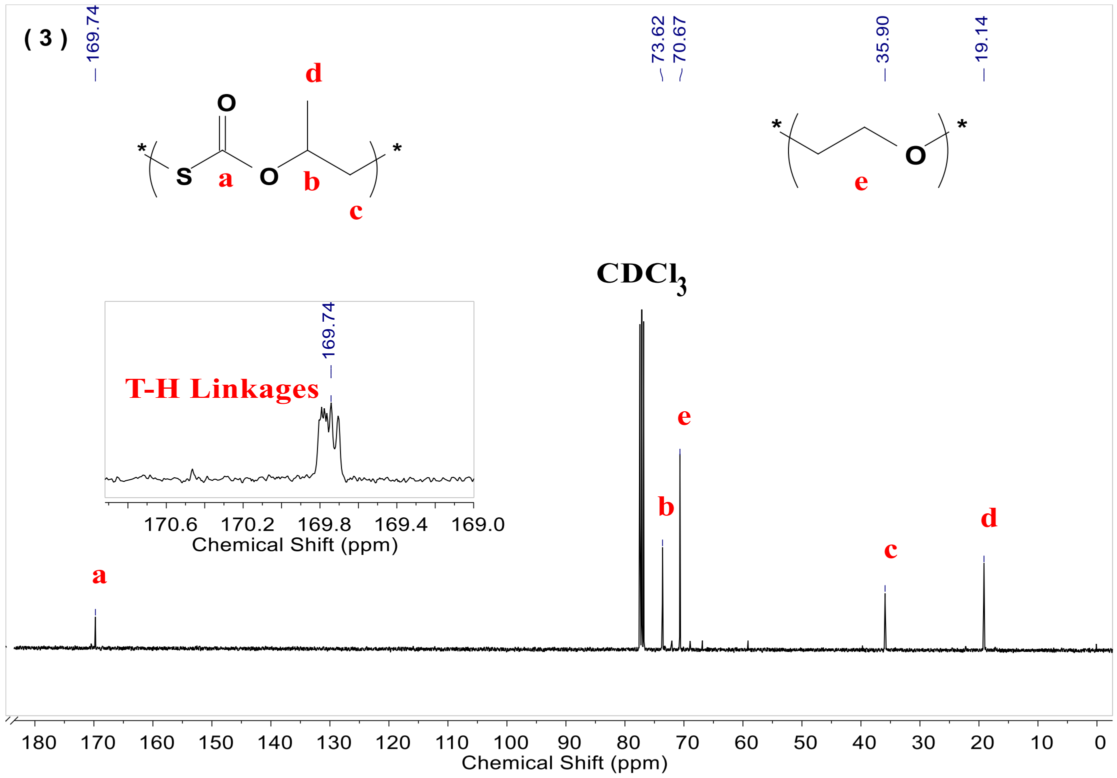

Figure S13. (1)^{1}H NMR spectrum of the crude product of entry 1, Table 2; (2) ^{1}H NMR spectrum of the purified product of entry 1, Table 2; (3) ^{13}C NMR spectrum of the purified product of entry 1, Table 2.

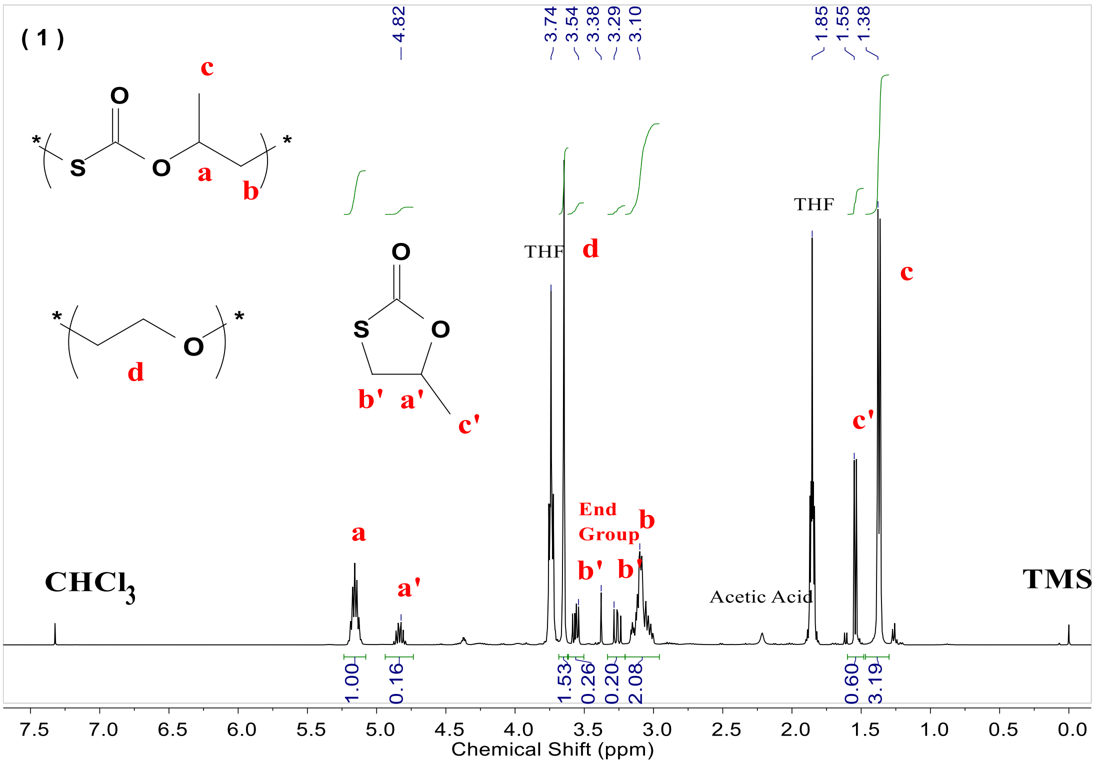
(1)
4.82
3.74
3.54
3.38
3.29
3.10
1.85
1.55
1.38
c
O
S
O
a
b
THF
d
THF
c
S
O
O
d
b'
a'
c'
c'
a
a'
End
Group
b
b'
b'
CHCl3
Acetic Acid
TMS
1.00
0.16
1.53
0.26
0.20
2.08
0.60
3.19
7.5 7.0 6.5 6.0 5.5 5.0 4.5 4.0 3.5 3.0 2.5 2.0 1.5 1.0 0.5 0.0
Chemical Shift (ppm)

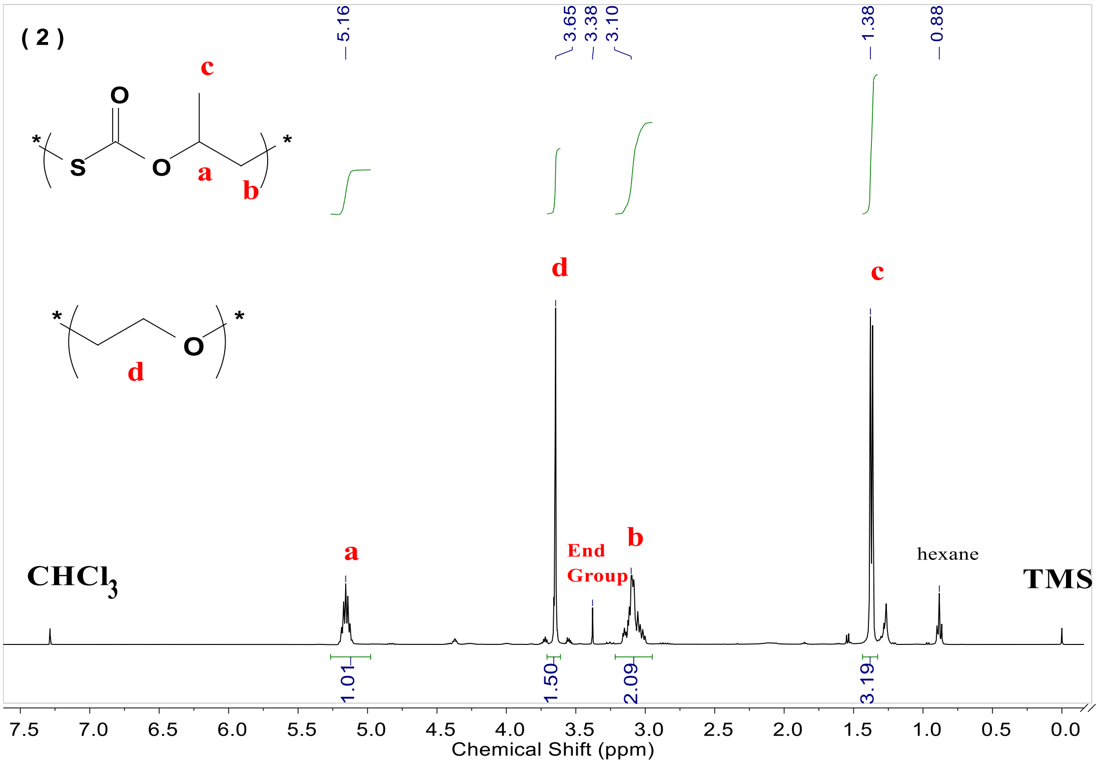
(2)
5.16
3.65
3.38
3.10
1.38
0.88
c
O
S
O
a
b
d
c
d
a
End
Group
b
hexane
CHCl3
TMS
1.01
1.50
2.09
3.19
7.5 7.0 6.5 6.0 5.5 5.0 4.5 4.0 3.5 3.0 2.5 2.0 1.5 1.0 0.5 0.0
Chemical Shift (ppm)

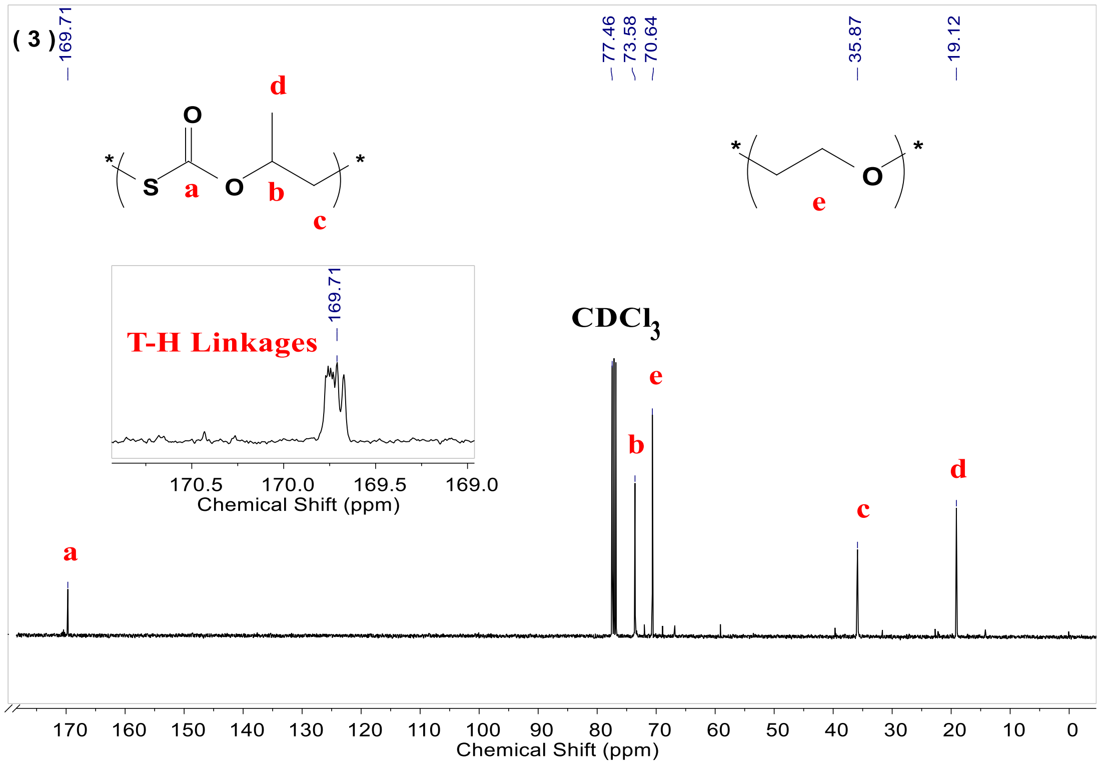

Figure S14. (1)^{1}H NMR spectrum of the crude product of entry 2, Table 2; (2) ^{1}H NMR spectrum of the purified product of entry 2, Table 2; (3) ^{13}C NMR spectrum of the purified product of entry 2, Table 2.

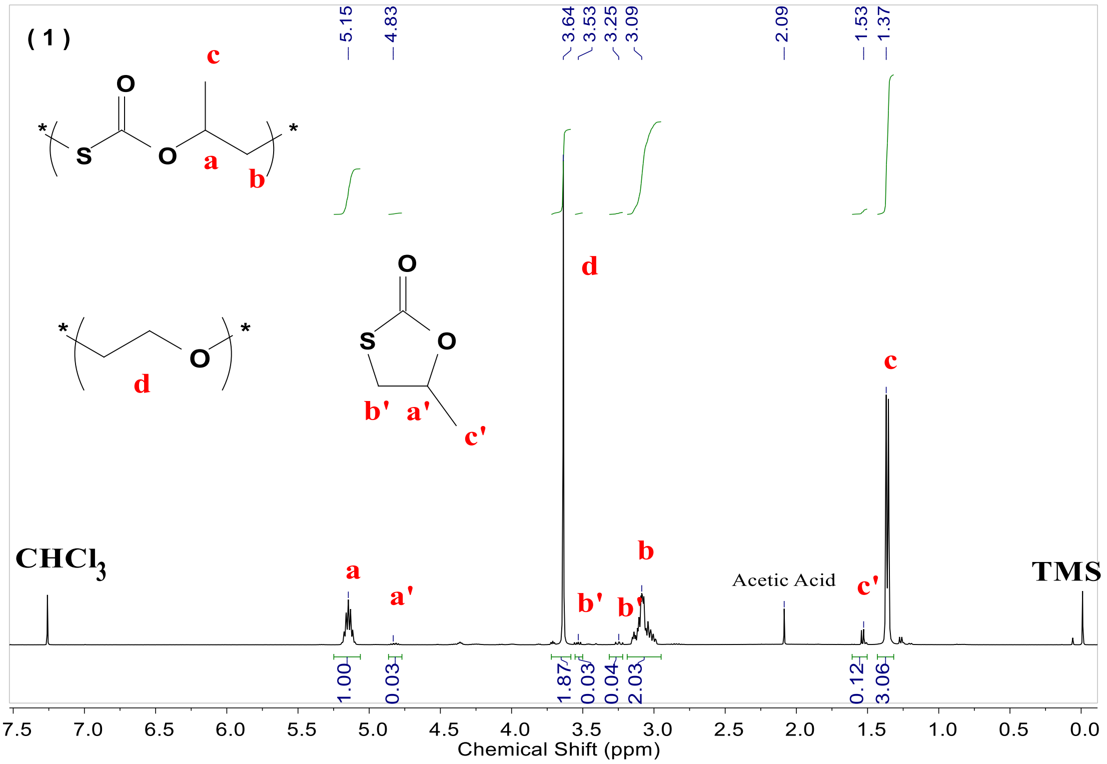

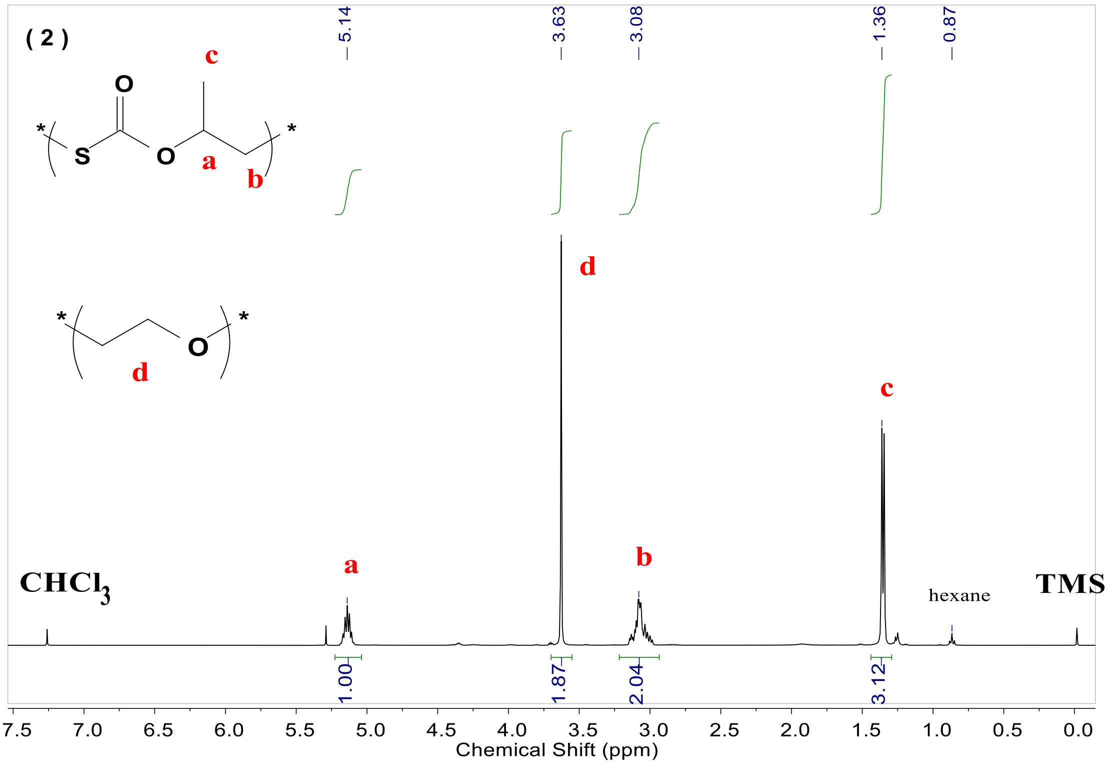

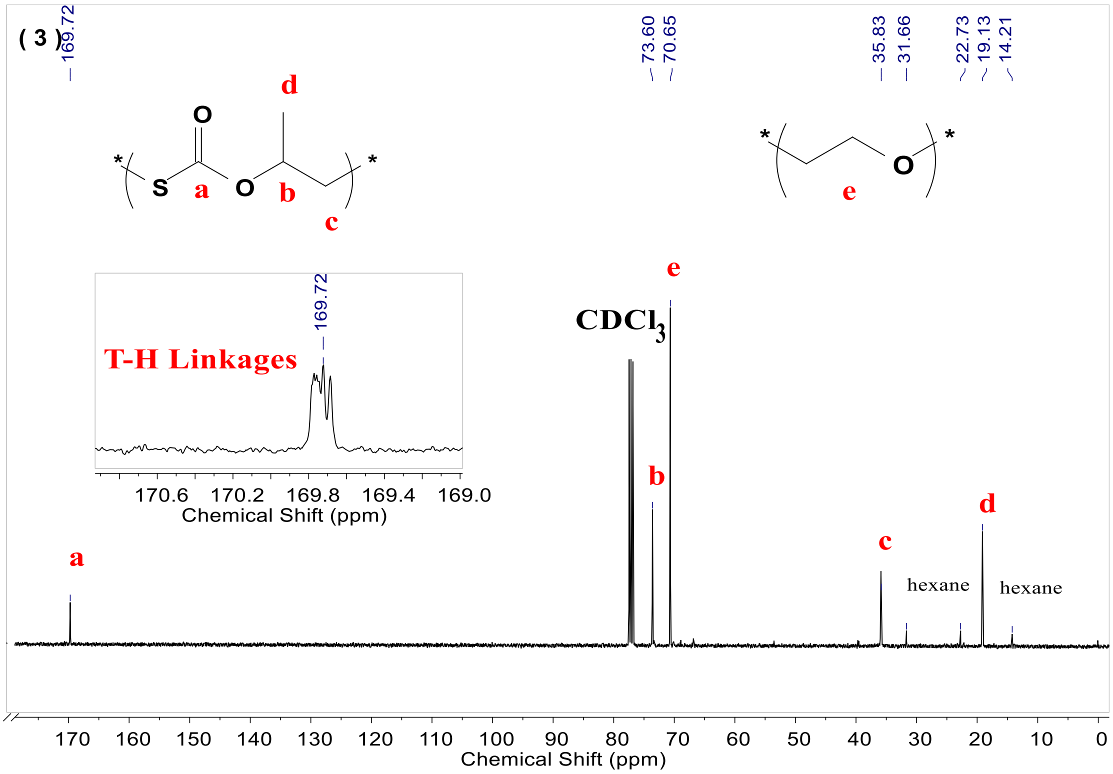

Figure S15. (1)^{1}H NMR spectrum of the crude product of entry 3, Table 2; (2) ^{1}H NMR spectrum of the purified product of entry 3, Table 2; (3) ^{13}C NMR spectrum of the purified product of entry 3, Table 2.

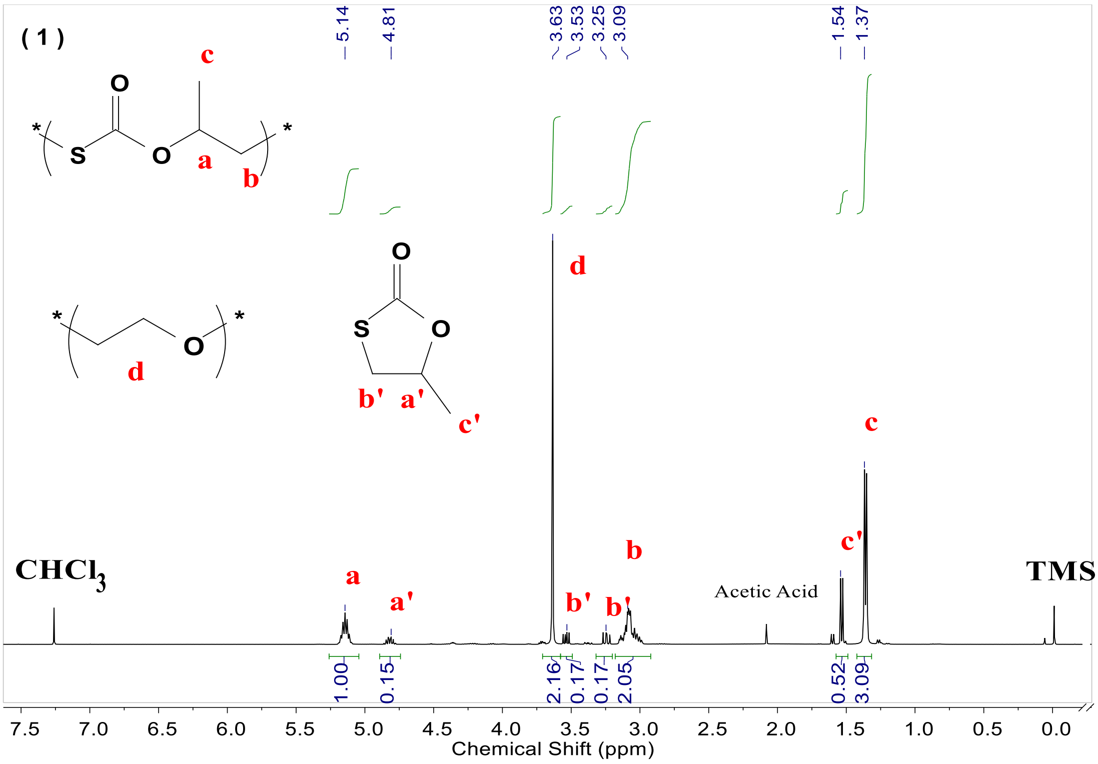
(1)
5.14
4.81
3.63
3.53
3.25
3.09
1.54
1.37
c
O
S
O
a
b
d
O
S
O
b'
a'
c'
CHCl3
Acetic Acid
TMS
1.00
0.15
2.16
0.17
0.17
2.05
0.52
3.09
7.5 7.0 6.5 6.0 5.5 5.0 4.5 4.0 3.5 3.0 2.5 2.0 1.5 1.0 0.5 0.0
Chemical Shift (ppm)

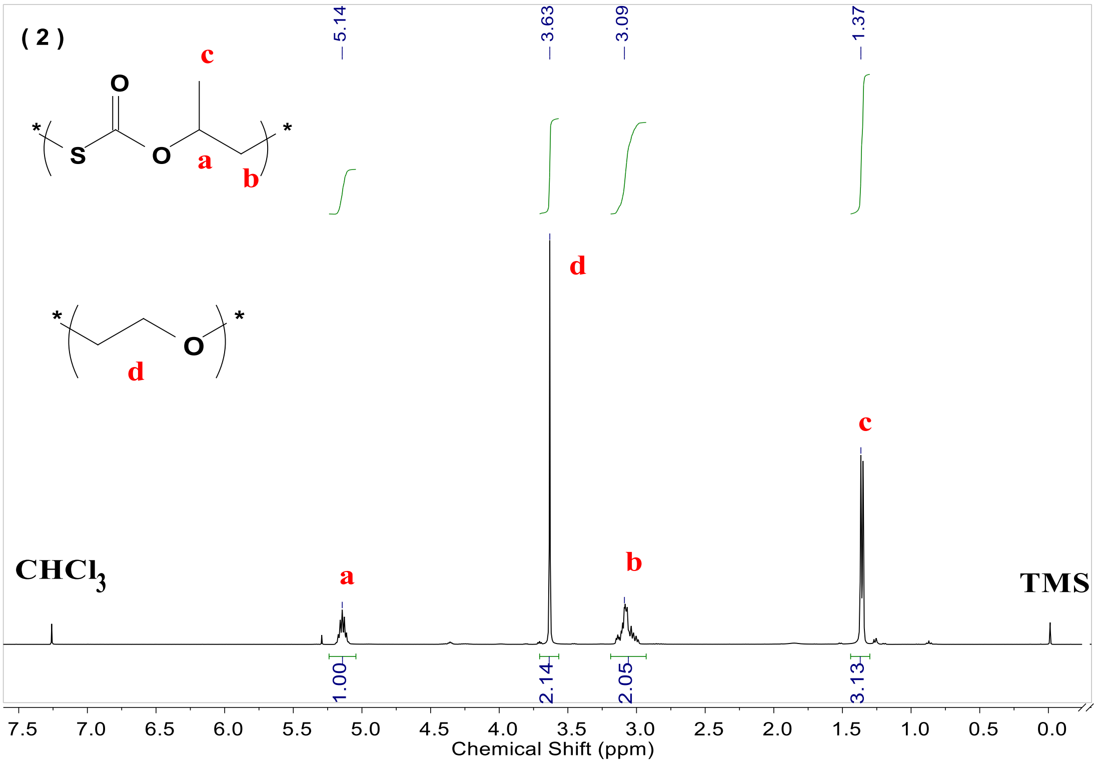
(2)
5.14
3.63
3.09
1.37
c
O
S
O
a
b
d
O
CHCl3
TMS
1.00
2.14
2.05
3.13
7.5 7.0 6.5 6.0 5.5 5.0 4.5 4.0 3.5 3.0 2.5 2.0 1.5 1.0 0.5 0.0
Chemical Shift (ppm)

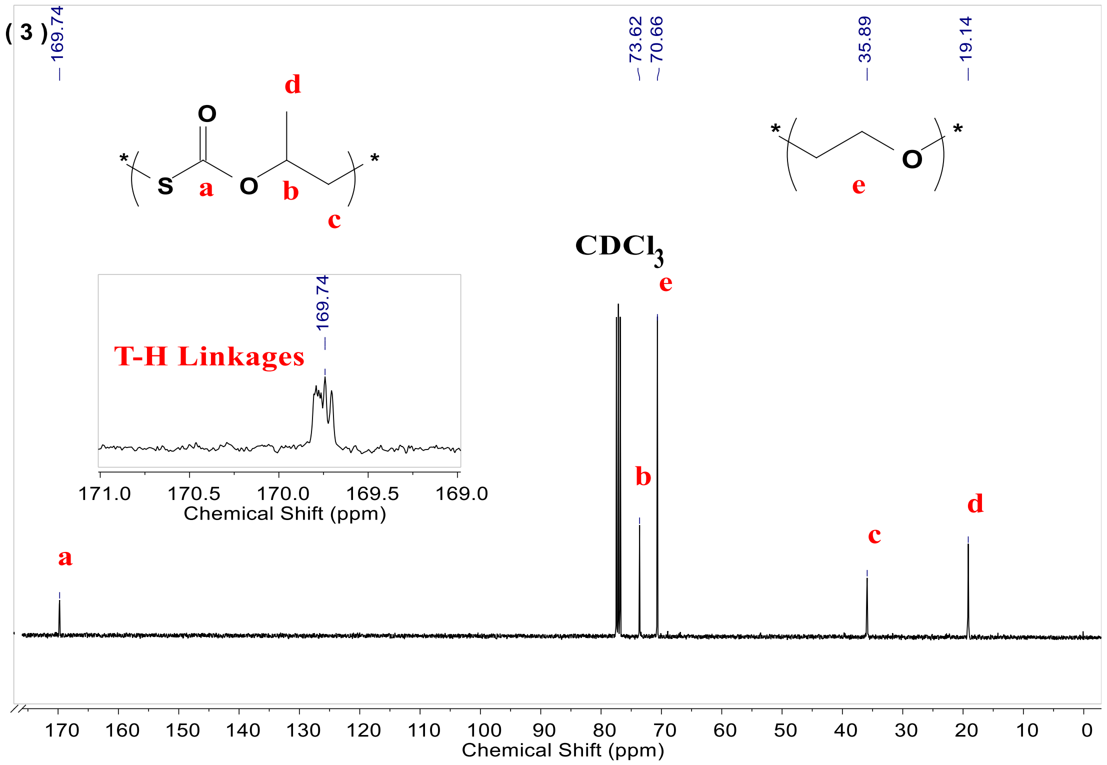

Figure S16. (1)^{1}H NMR spectrum of the crude product of entry 4, Table 2; (2) ^{1}H NMR spectrum of the purified product of entry 4, Table 2; (3) ^{13}C NMR spectrum of the purified product of entry 4, Table 2.

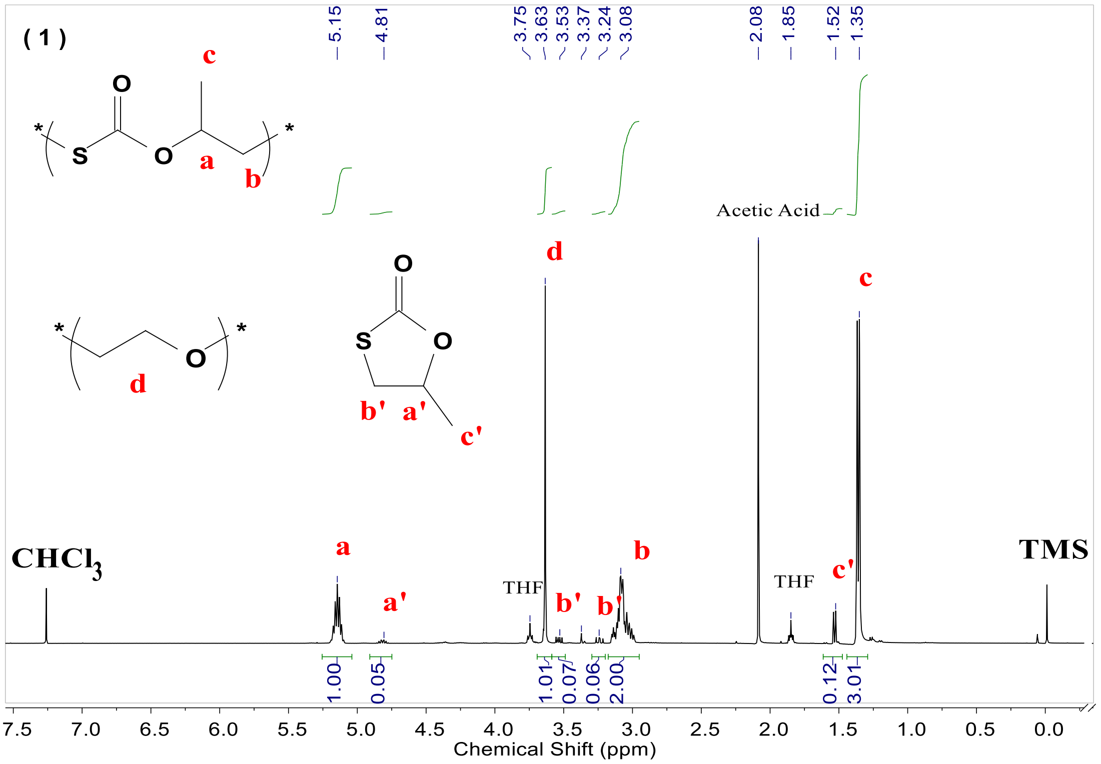

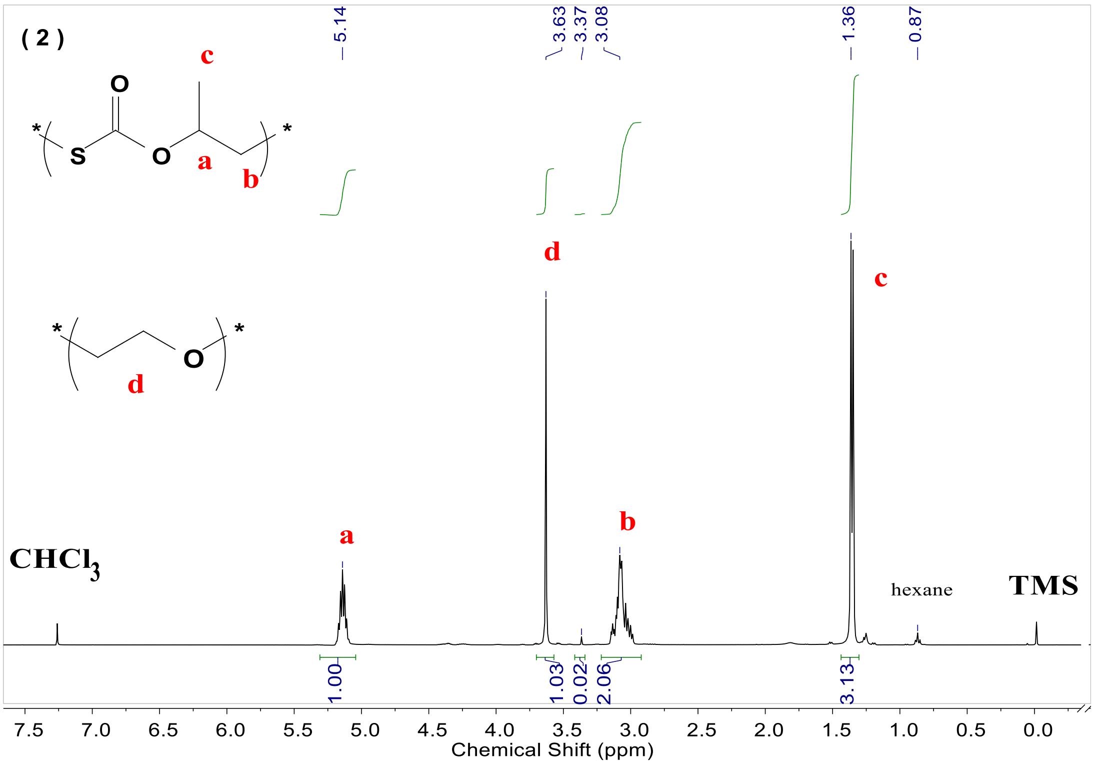

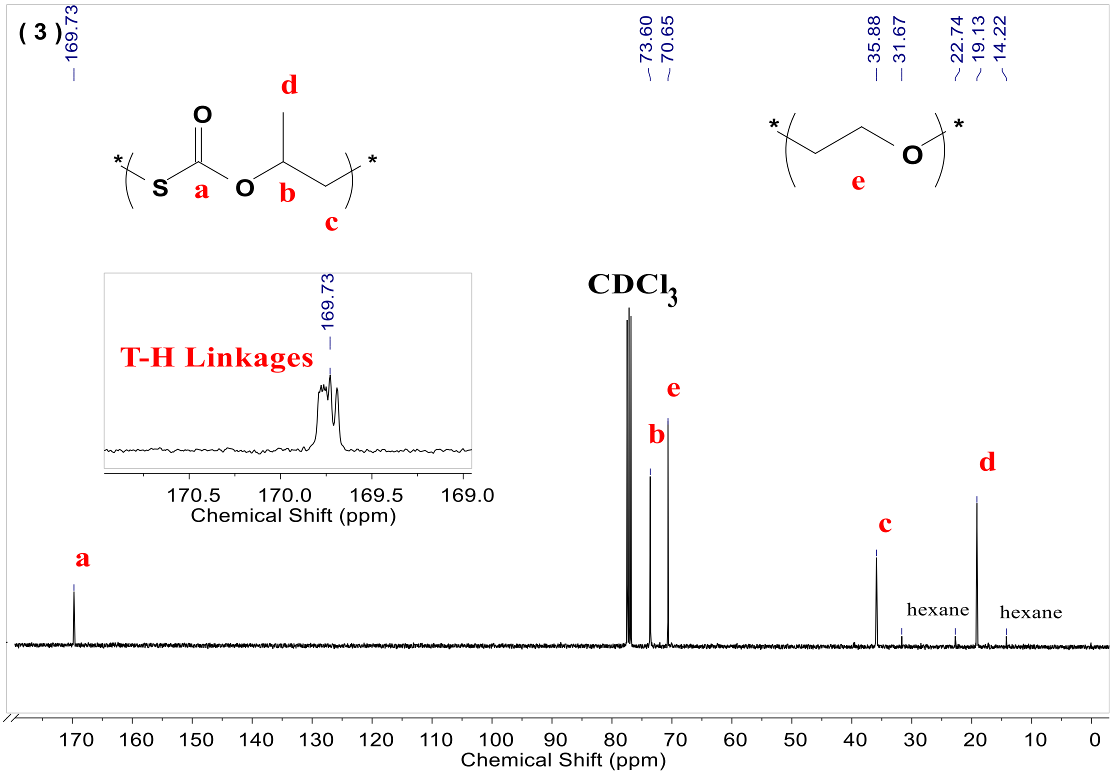

Figure S17. (1)^{1}H NMR spectrum of the crude product of entry 5, Table 2; (2) ^{1}H NMR spectrum of the purified product of entry 5, Table 2; (3) ^{13}C NMR spectrum of the purified product of entry 5, Table 2.

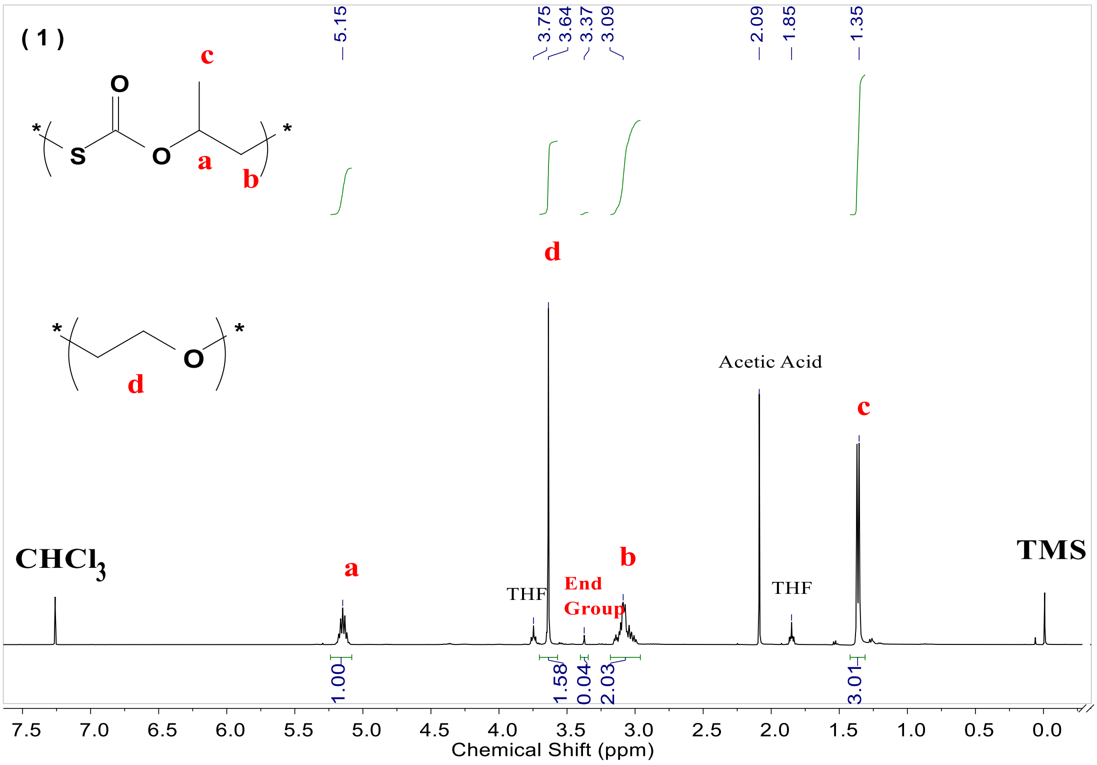
(1)
5.15
3.75
3.64
3.37
3.09
2.09
1.85
1.35
c
O
S
O
a
b
d
O
d
Acetic Acid
c
CHCl3
a
THF
End
Group
b
THF
TMS
1.00
1.58
0.04
2.03
3.01
7.5 7.0 6.5 6.0 5.5 5.0 4.5 4.0 3.5 3.0 2.5 2.0 1.5 1.0 0.5 0.0
Chemical Shift (ppm)

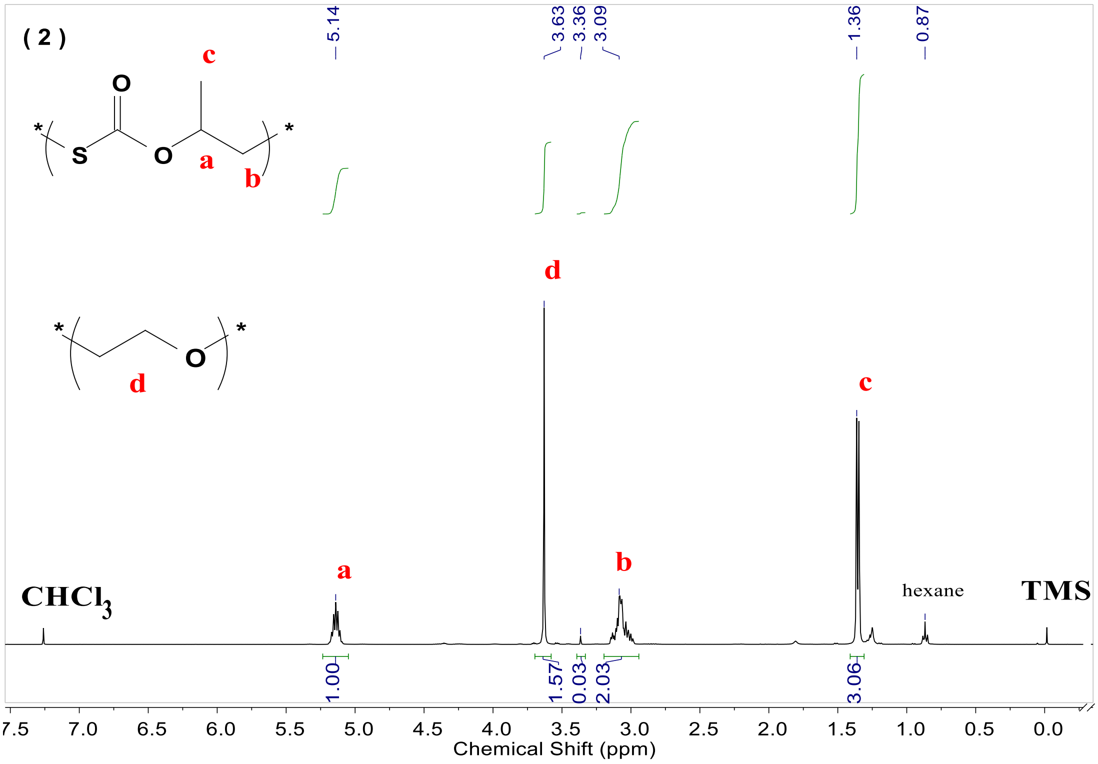
(2)
5.14
3.63
3.36
3.09
1.36
0.87
c
O
S
O
a
b
d
O
d
c
b
a
CHCl3
hexane
TMS
1.00
1.57
0.03
2.03
3.06
7.5 7.0 6.5 6.0 5.5 5.0 4.5 4.0 3.5 3.0 2.5 2.0 1.5 1.0 0.5 0.0
Chemical Shift (ppm)

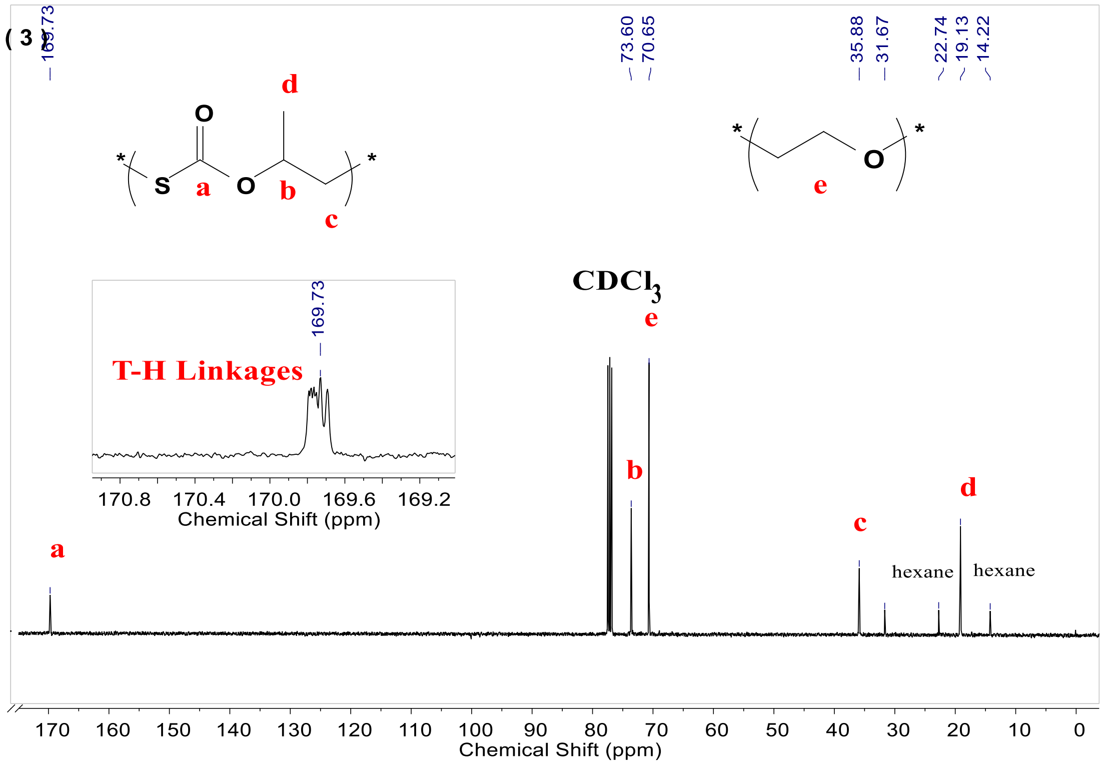

Figure S18. (1)^{1}H NMR spectrum of the crude product of entry 6, Table 2; (2) ^{1}H NMR spectrum of the purified product of entry 6, Table 2; (3) ^{13}C NMR spectrum of the purified product of entry 6, Table 2.

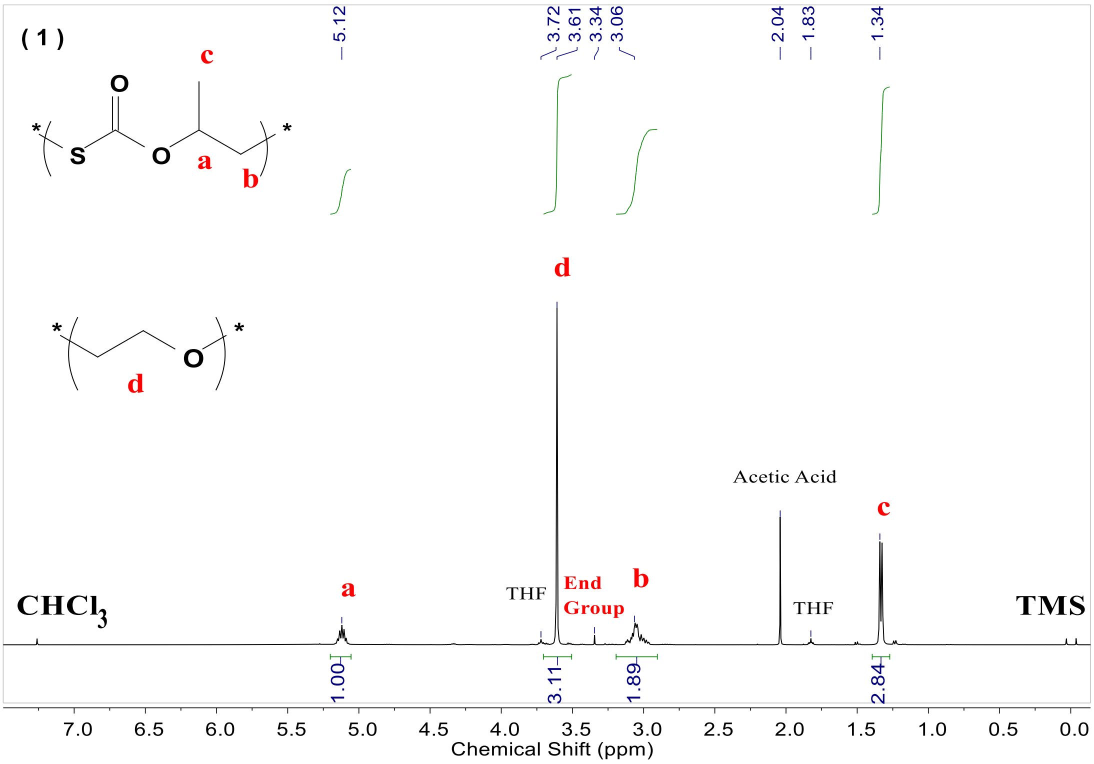

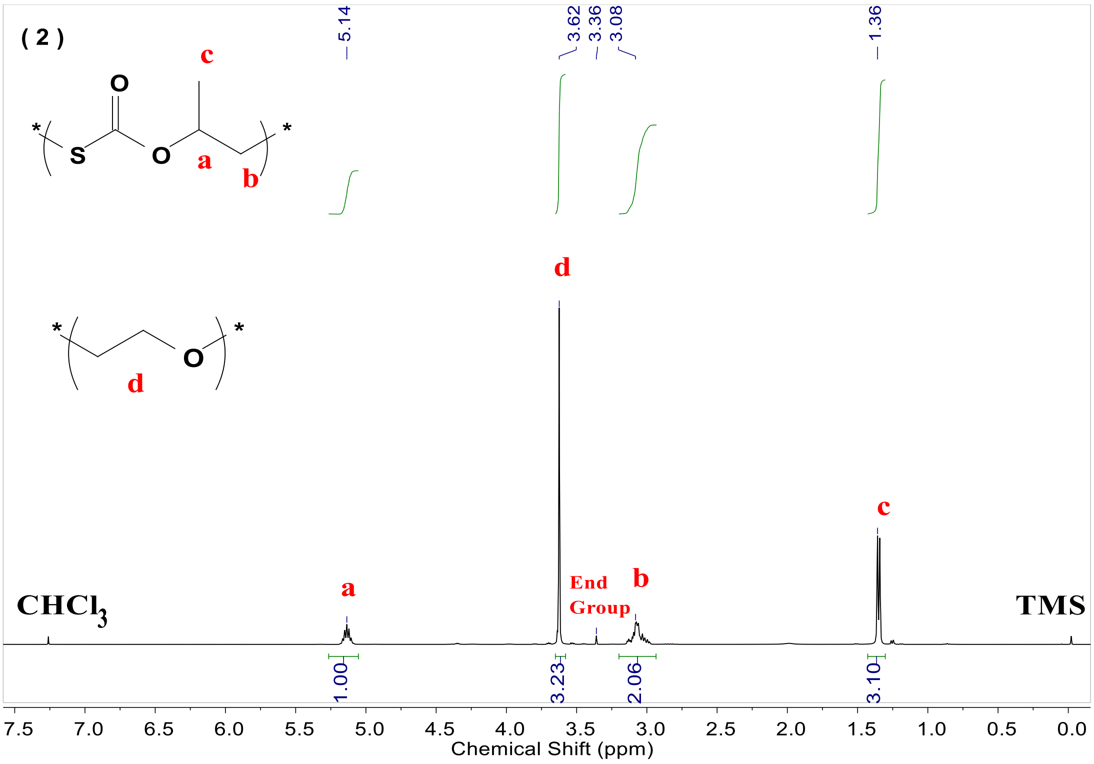

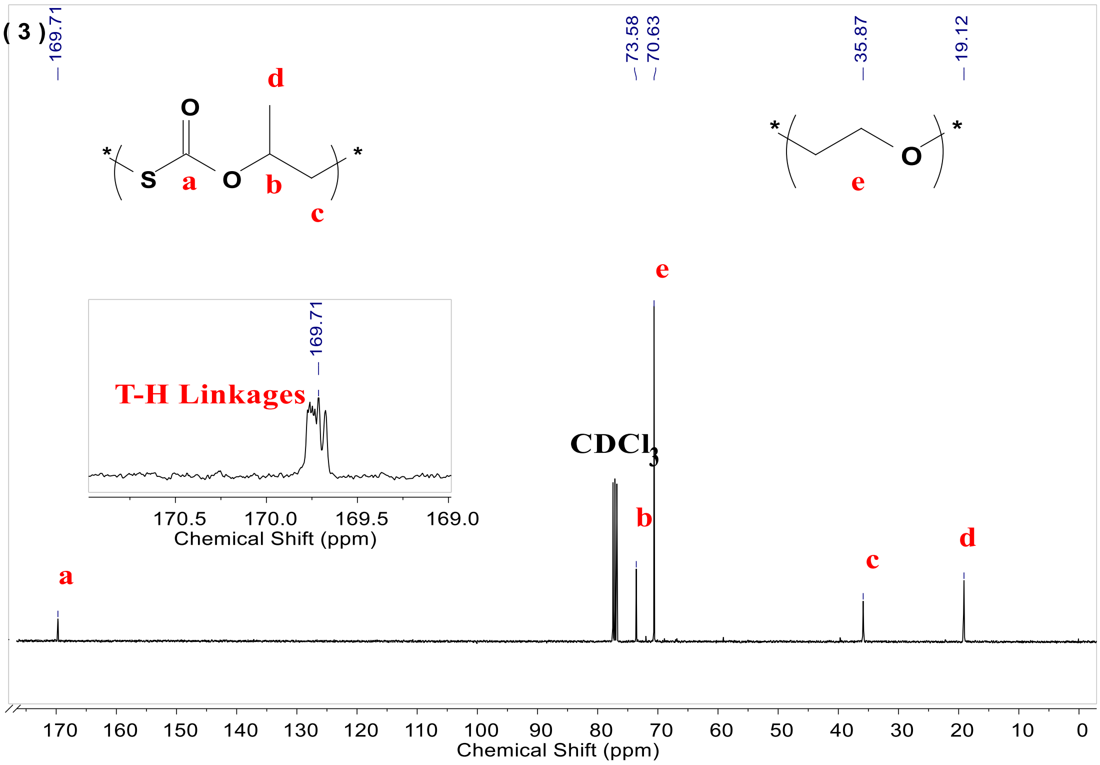

Figure S19. (1)^{1}H NMR spectrum of the crude product of entry 7, Table 2; (2) ^{1}H NMR spectrum of the purified product of entry 7, Table 2; (3) ^{13}C NMR spectrum of the purified product of entry 7, Table 2.

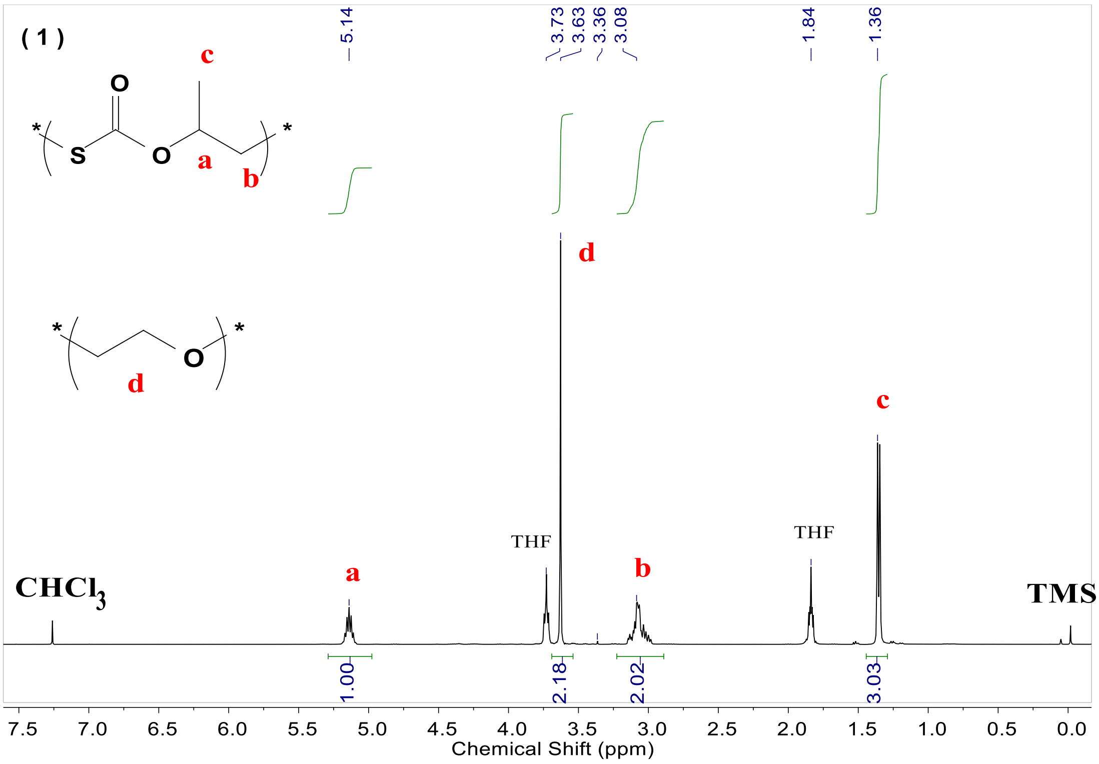
(1)
5.14
3.73
3.63
3.36
3.08
1.84
1.36
a
b
c
d
THF
THF
CHCl3
TMS
1.00
2.18
2.02
3.03
7.5 7.0 6.5 6.0 5.5 5.0 4.5 4.0 3.5 3.0 2.5 2.0 1.5 1.0 0.5 0.0
Chemical Shift (ppm)

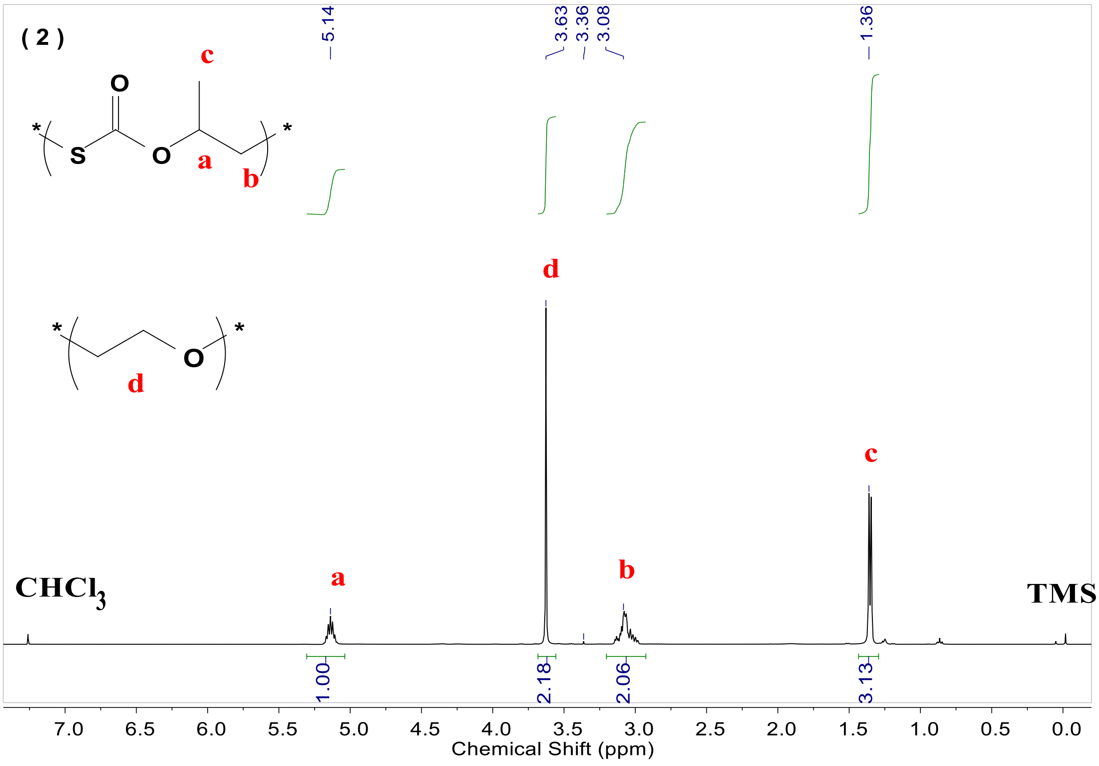
(2)
5.14
3.63
3.36
3.08
1.36
a
b
c
d
CHCl3
TMS
1.00
2.18
2.06
3.13
7.0 6.5 6.0 5.5 5.0 4.5 4.0 3.5 3.0 2.5 2.0 1.5 1.0 0.5 0.0
Chemical Shift (ppm)

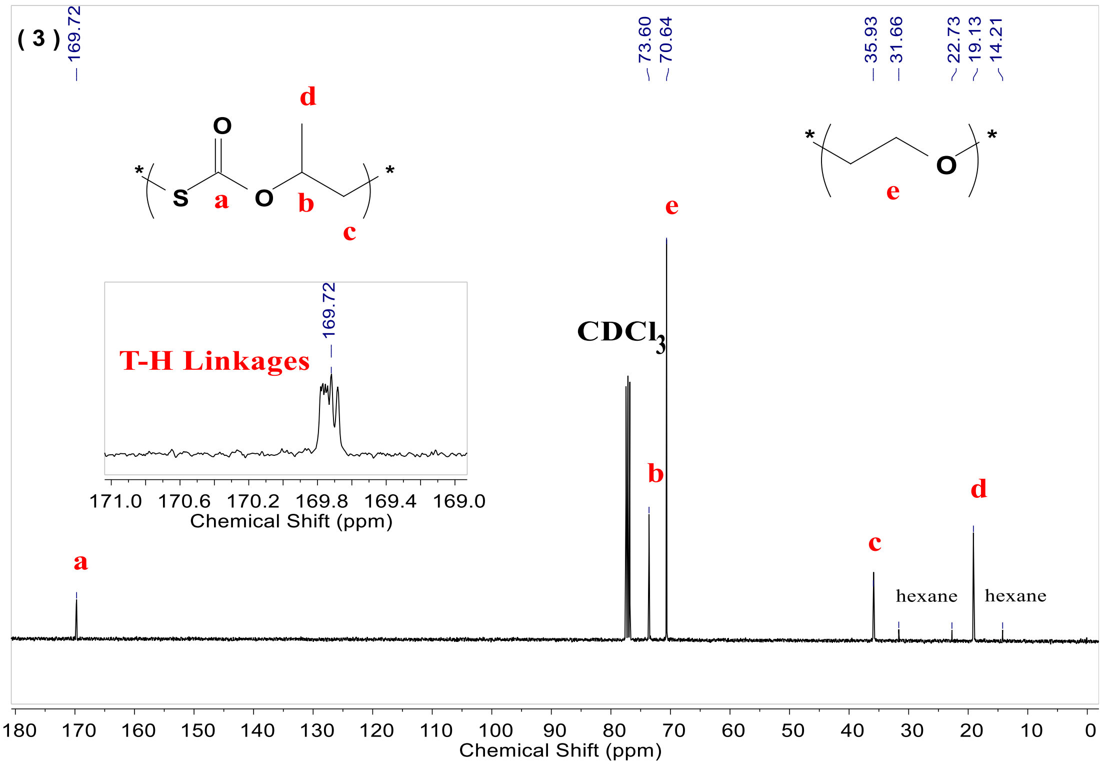

Figure S20. (1)^{1}H NMR spectrum of the crude product of entry 8, Table 2; (2) ^{1}H NMR spectrum of the purified product of entry 8, Table 2; (3) ^{13}C NMR spectrum of the purified product of entry 8, Table 2.

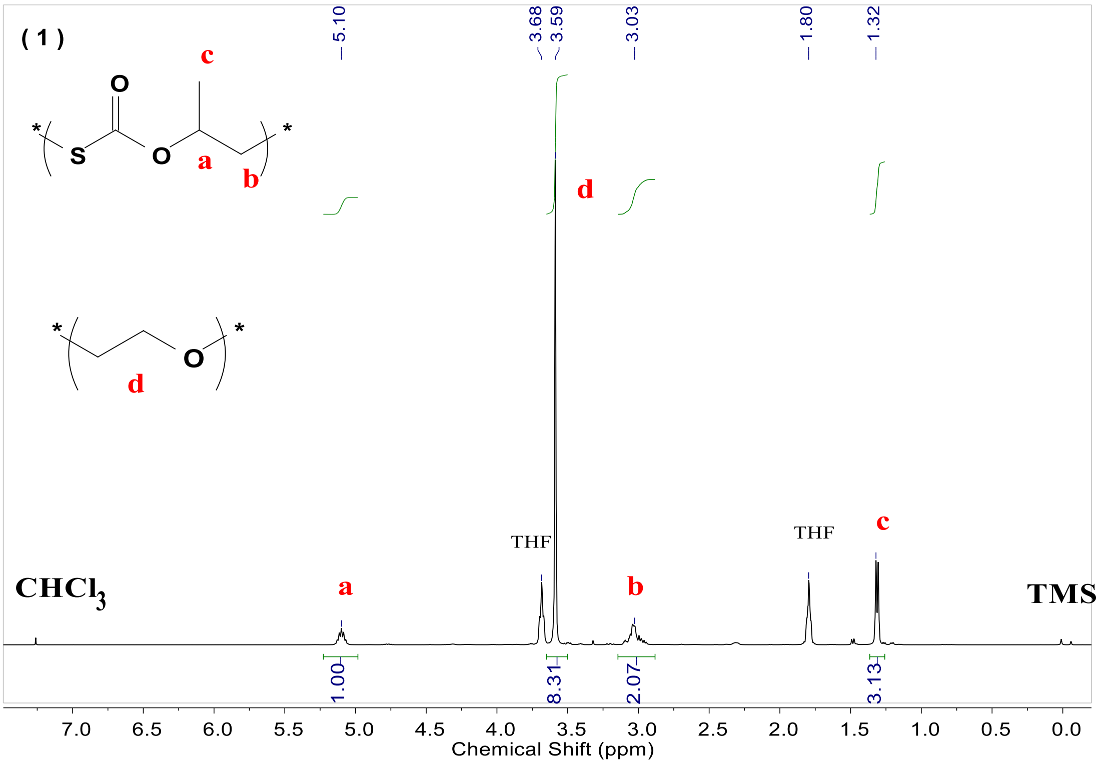

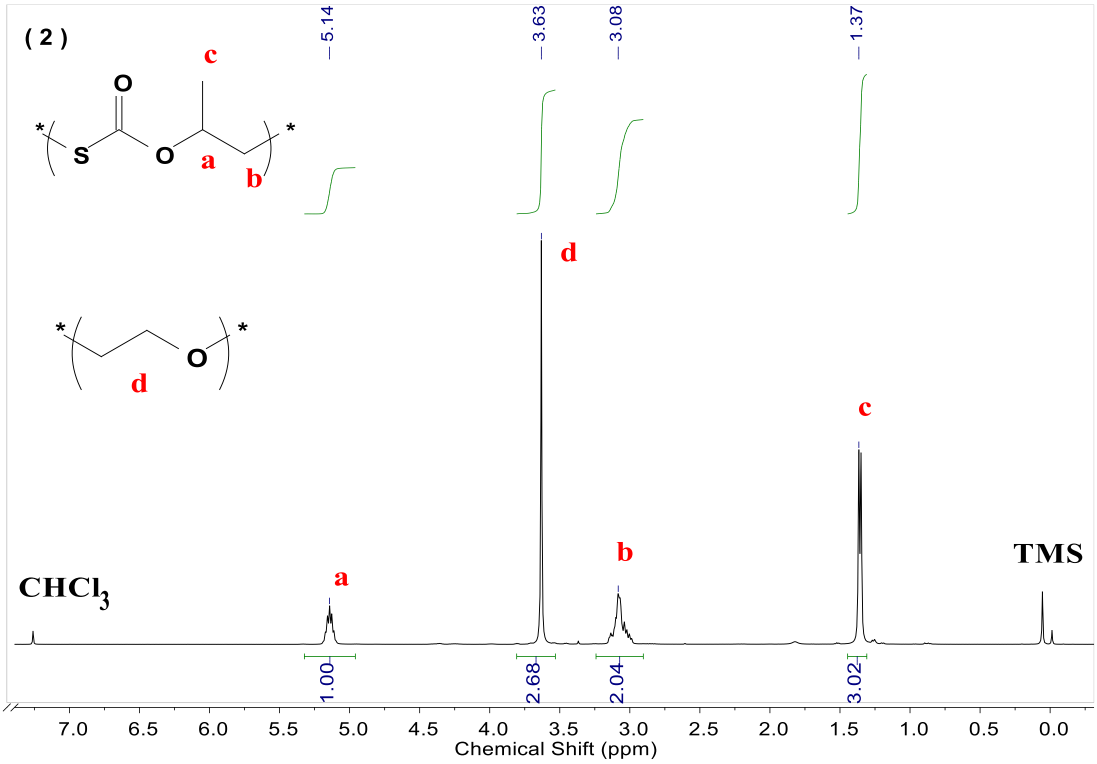

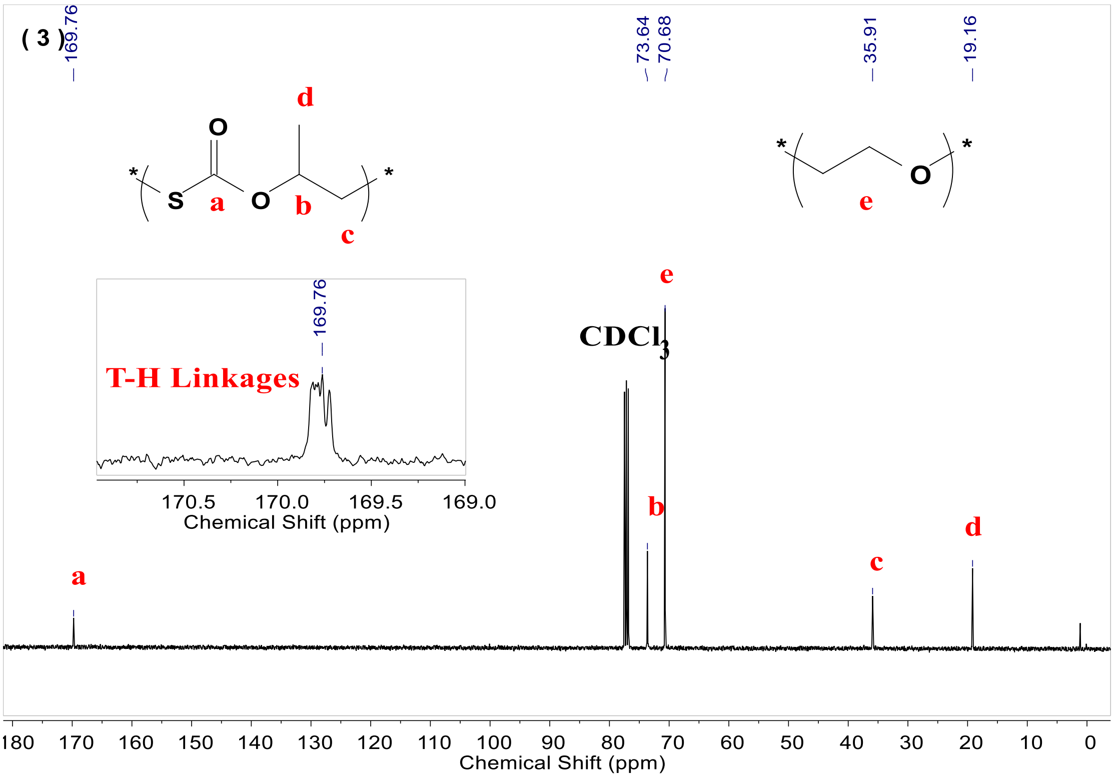

Figure S21. (1)^{1}H NMR spectrum of the crude product of entry 9, Table 2; (2) ^{1}H NMR spectrum of the purified product of entry 9, Table 2; (3) ^{13}C NMR spectrum of the purified product of entry 9, Table 2.

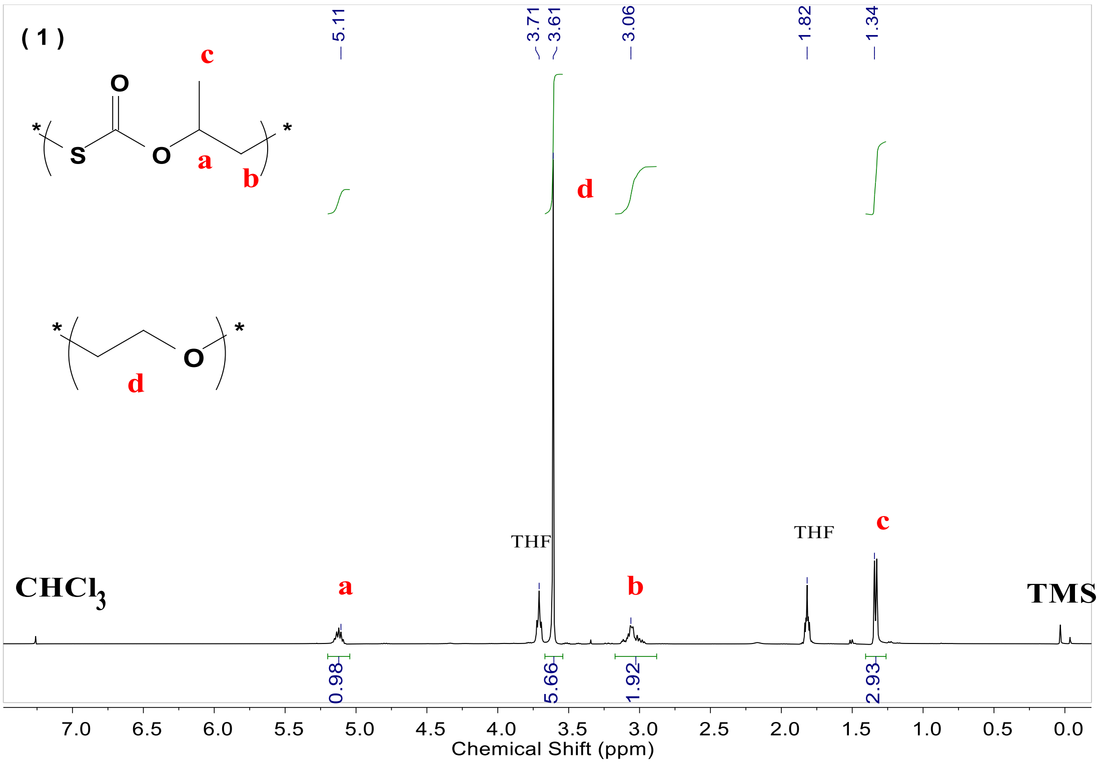

(1)
5.11
3.71
3.61
3.06
1.82
1.34
c
O
S
O
a
b
d
THF
THF
c
CHCl3
a
b
TMS
0.98
5.66
1.92
2.93
7.0 6.5 6.0 5.5 5.0 4.5 4.0 3.5 3.0 2.5 2.0 1.5 1.0 0.5 0.0
Chemical Shift (ppm)

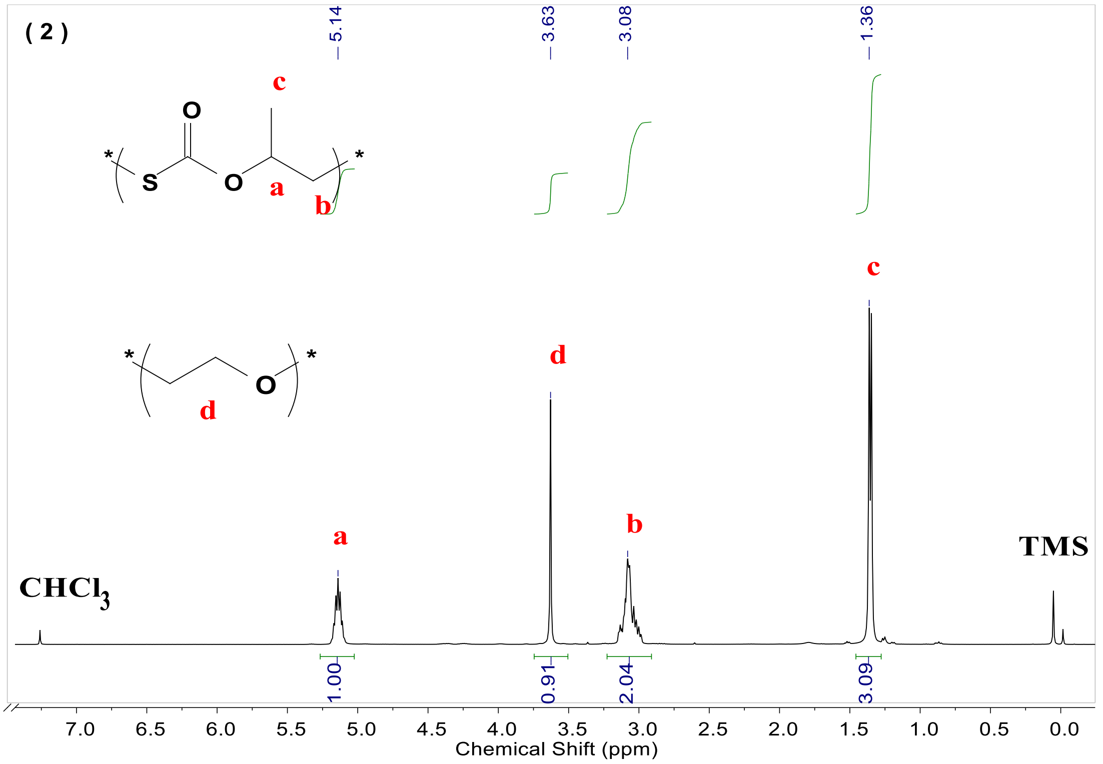

(2)
5.14
3.63
3.08
1.36
c
O
S
O
a
b
d
c
d
a
b
CHCl3
TMS
1.00
0.91
2.04
3.09
7.0 6.5 6.0 5.5 5.0 4.5 4.0 3.5 3.0 2.5 2.0 1.5 1.0 0.5 0.0
Chemical Shift (ppm)

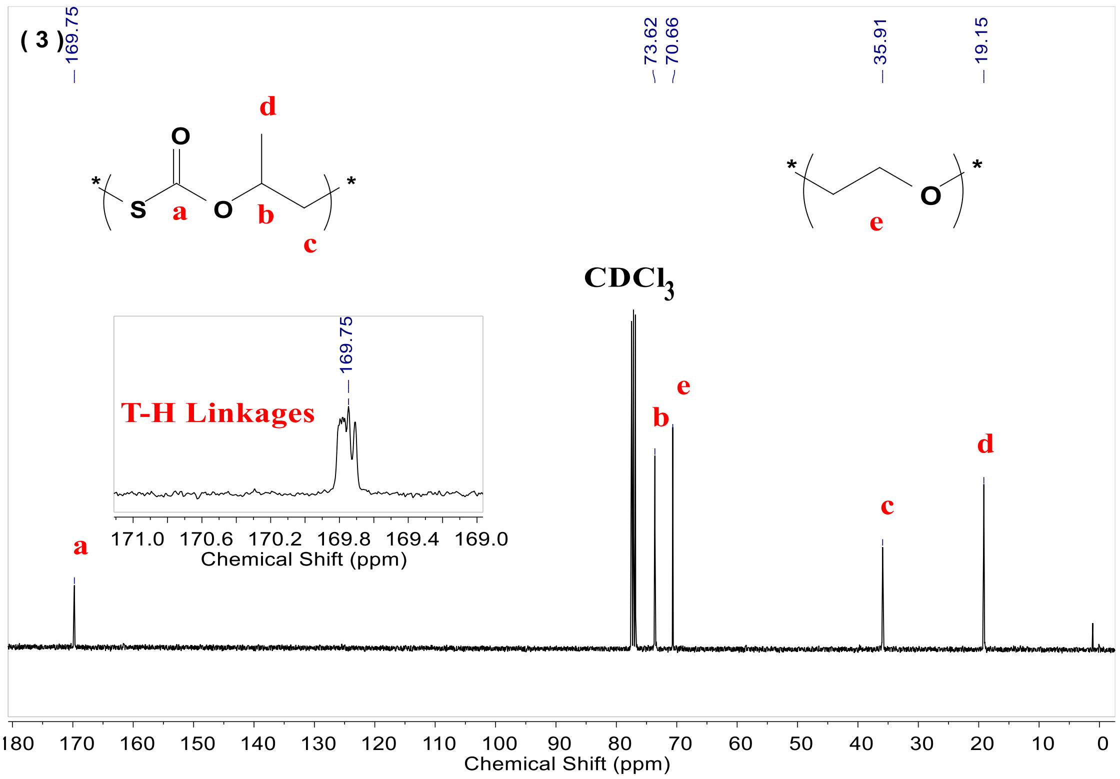

Figure S22. (1)^{1}H NMR spectrum of the crude product of entry 10, Table 2; (2) ^{1}H NMR spectrum of the purified product of entry 10, Table 2; (3) ^{13}C NMR spectrum of the purified product of entry 10, Table 2.

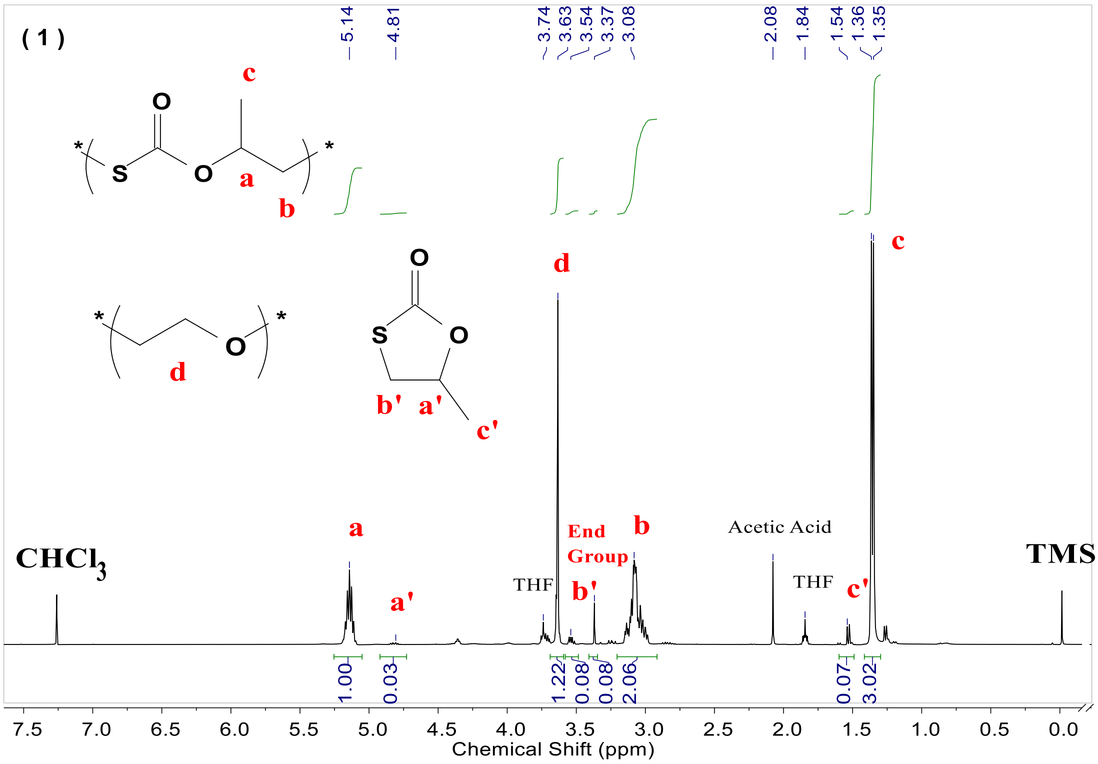

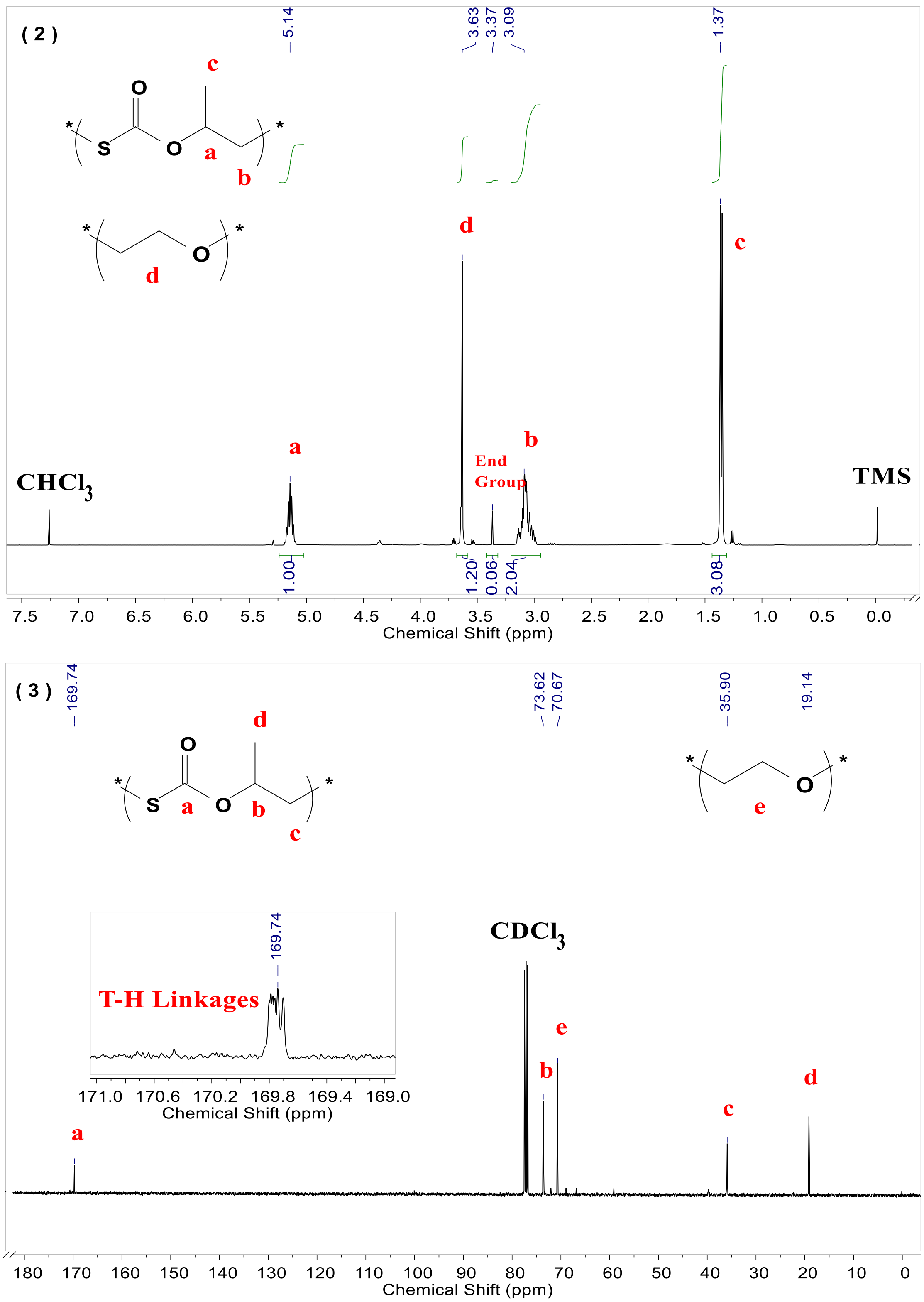

Figure S23. (1)^{1}H NMR spectrum of the crude product of entry 11, Table 2; (2) ^{1}H NMR spectrum of the purified product of entry 11, Table 2; (3) ^{13}C NMR spectrum of the purified product of entry 11, Table 2.

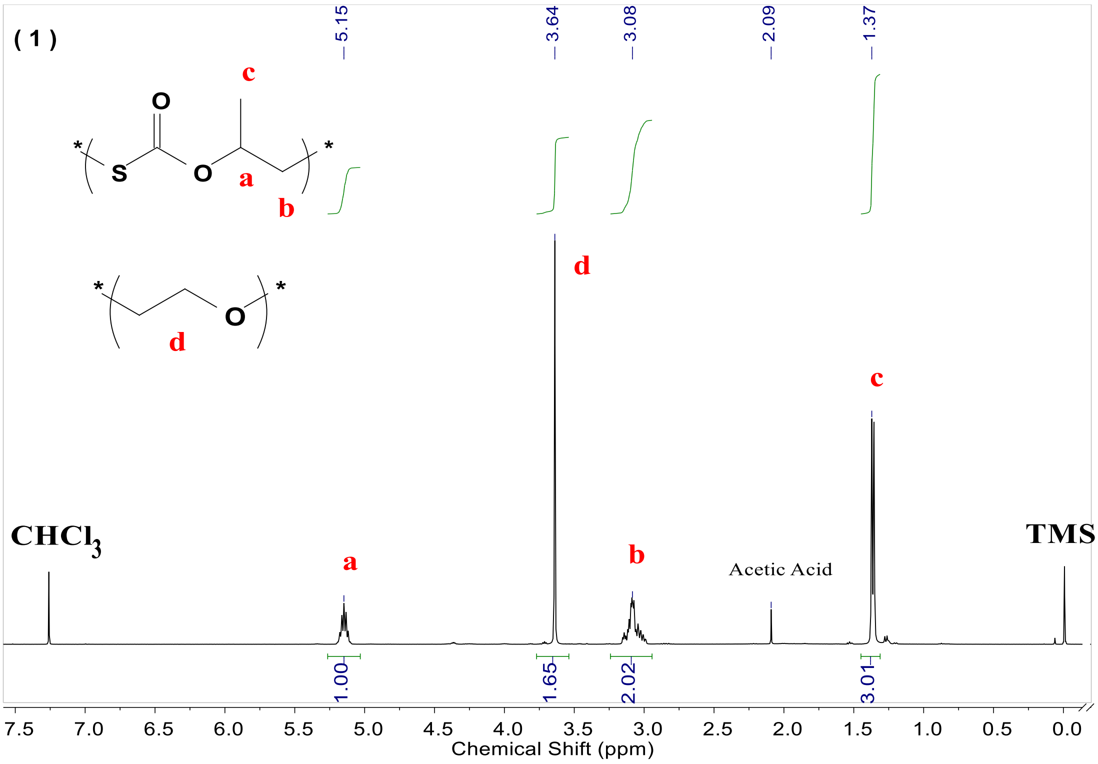

(1)
5.15
3.64
3.08
2.09
1.37
c
O
S
O
a
b
d
O
d
c
CHCl3
a
b
Acetic Acid
TMS
1.00
1.65
2.02
3.01
7.5 7.0 6.5 6.0 5.5 5.0 4.5 4.0 3.5 3.0 2.5 2.0 1.5 1.0 0.5 0.0
Chemical Shift (ppm)

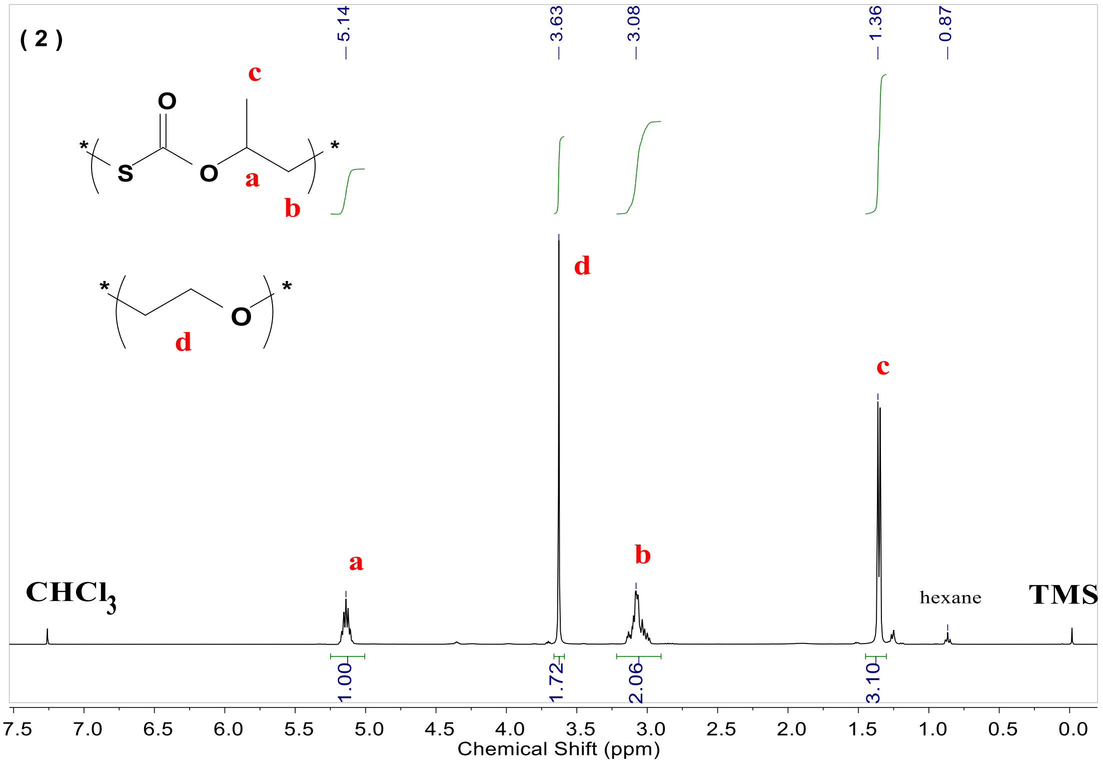

(2)
5.14
3.63
3.08
1.36
0.87
c
O
S
O
a
b
d
O
d
c
CHCl3
a
b
hexane
TMS
1.00
1.72
2.06
3.10
7.5 7.0 6.5 6.0 5.5 5.0 4.5 4.0 3.5 3.0 2.5 2.0 1.5 1.0 0.5 0.0
Chemical Shift (ppm)

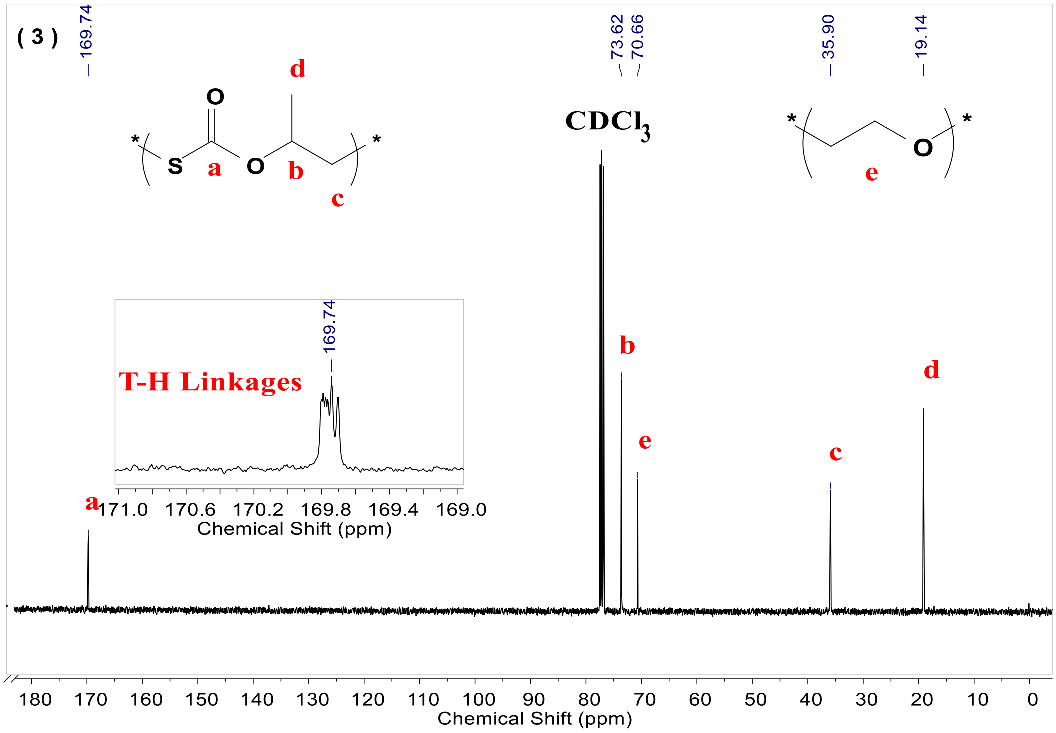

Figure S24. (1)^{1}H NMR spectrum of the crude product of entry 12, Table 2; (2) ^{1}H NMR spectrum of the purified product of entry 12, Table 2; (3) ^{13}C NMR spectrum of the purified product of entry 12, Table 2.

Spectra of results listed in Table 3.

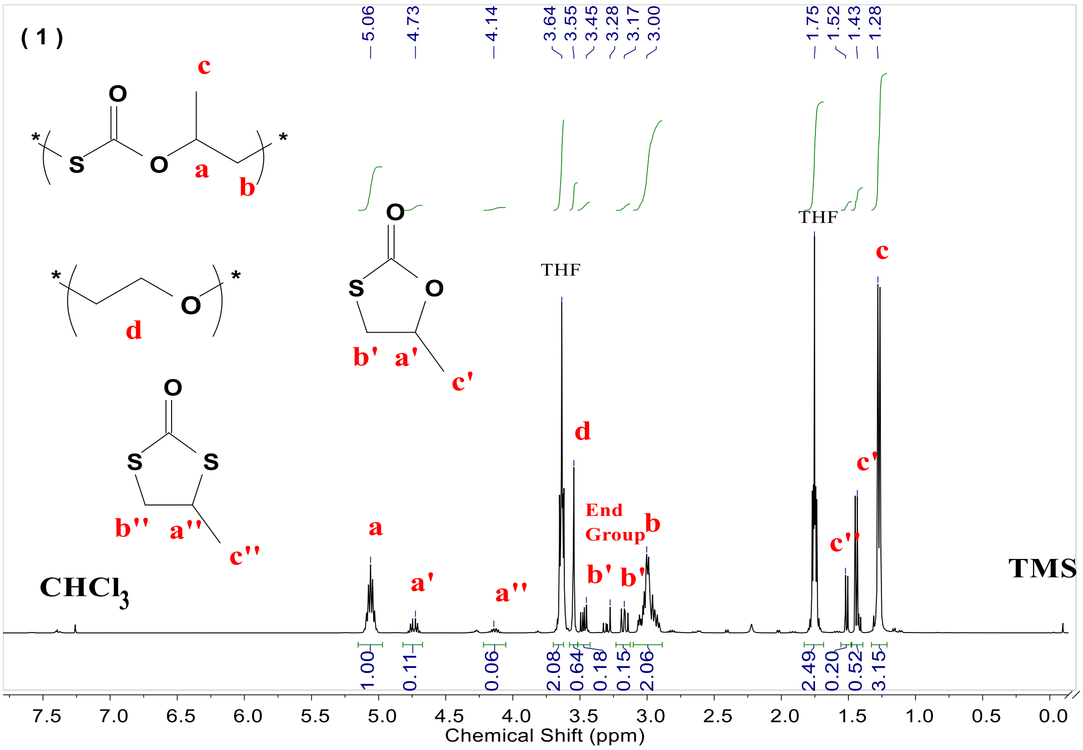

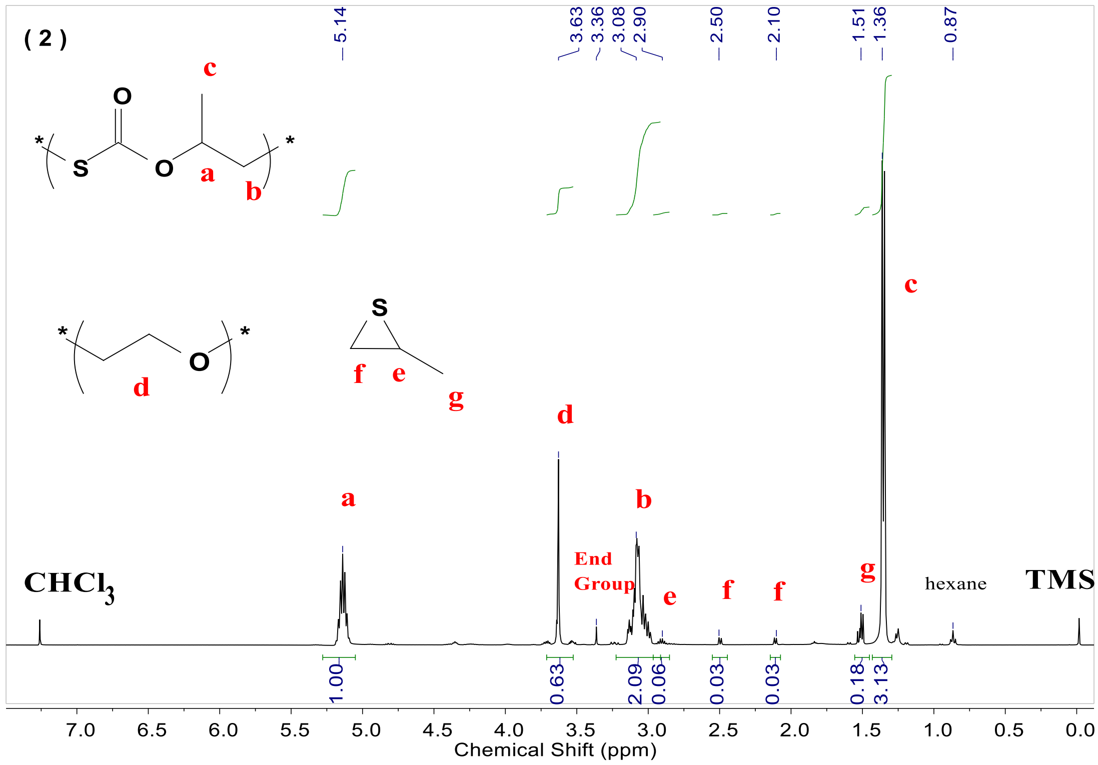

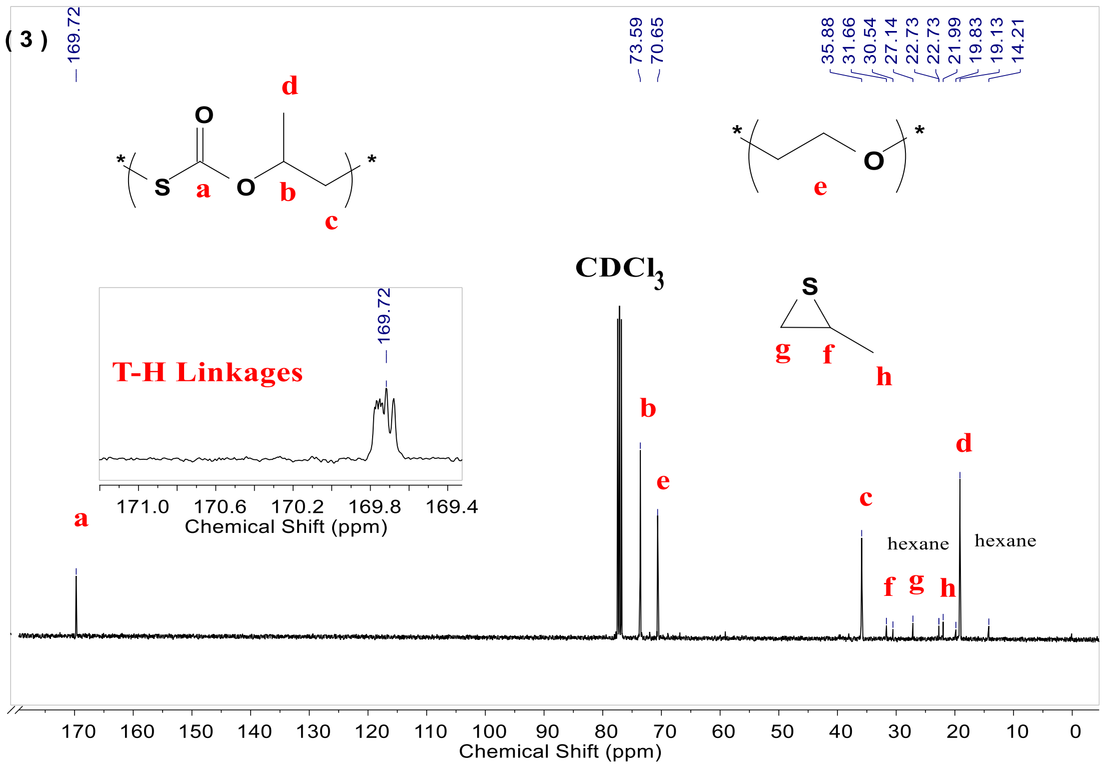

Figure S25. (1)^{1}H NMR spectrum of the crude product of entry 1, Table 3; (2) ^{1}H NMR spectrum of the purified product of entry 1, Table 3; (3) ^{13}C NMR spectrum of the purified product of entry 1, Table 3.

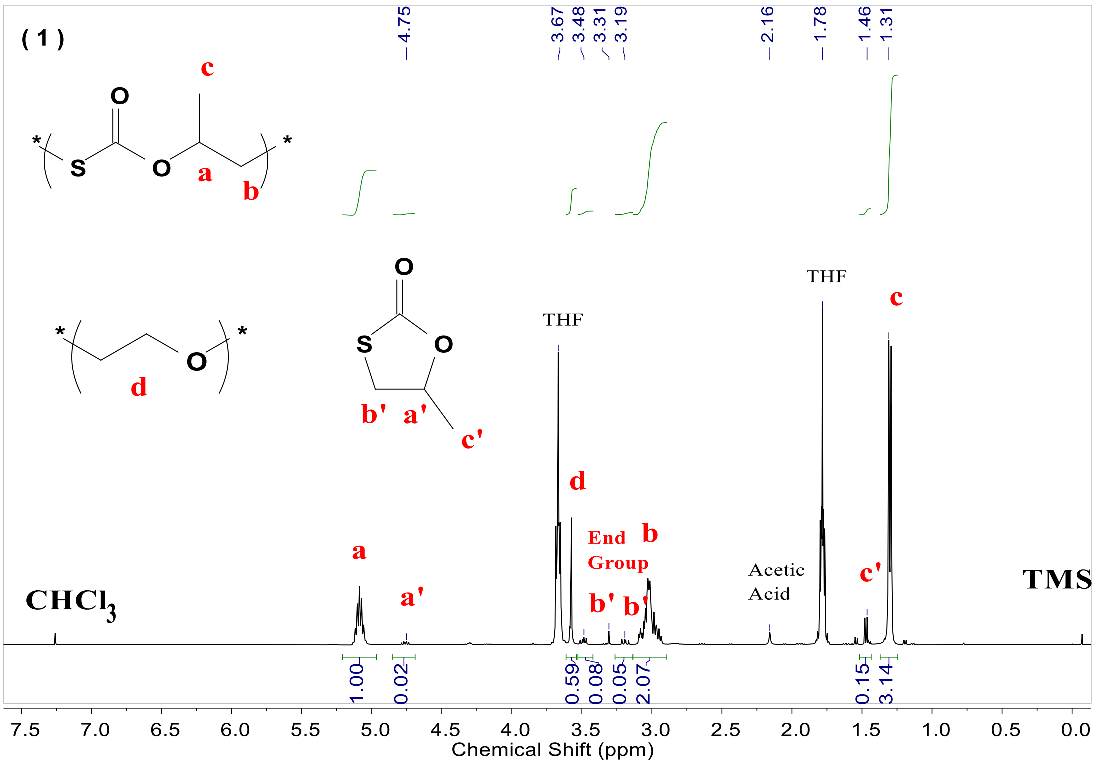

(1)
c
a
b
d
b'
a'
c'
THF
THF
c
d
End Group
b
a
a'
b'
b'
Acetic Acid
c'
CHCl3
TMS
4.75
3.67
3.48
3.31
3.19
2.16
1.78
1.46
1.31
1.00
0.02
0.59
0.08
0.05
2.07
0.15
3.14
Chemical Shift (ppm)

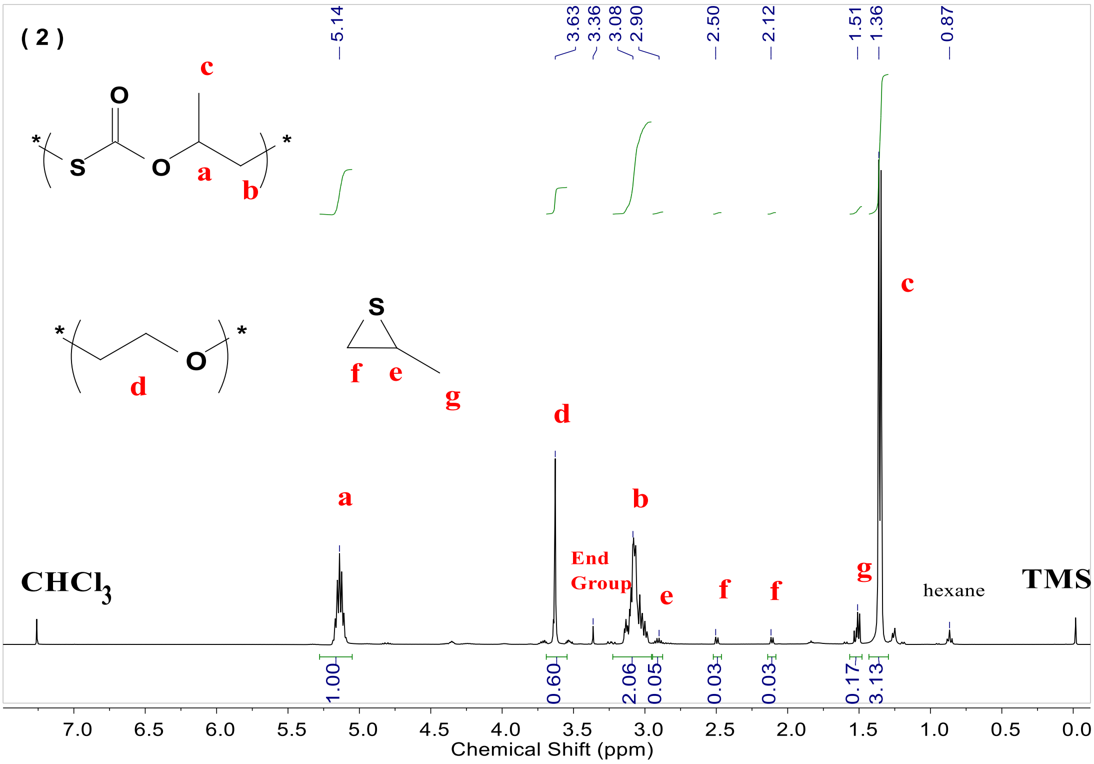

(2)
c
a
b
d
f
e
g
c
d
a
b
End Group
e
f
f
g
hexane
CHCl3
TMS
5.14
3.63
3.36
3.08
2.90
2.50
2.12
1.51
1.36
0.87
1.00
0.60
2.06
0.05
0.03
0.03
0.17
3.13
Chemical Shift (ppm)

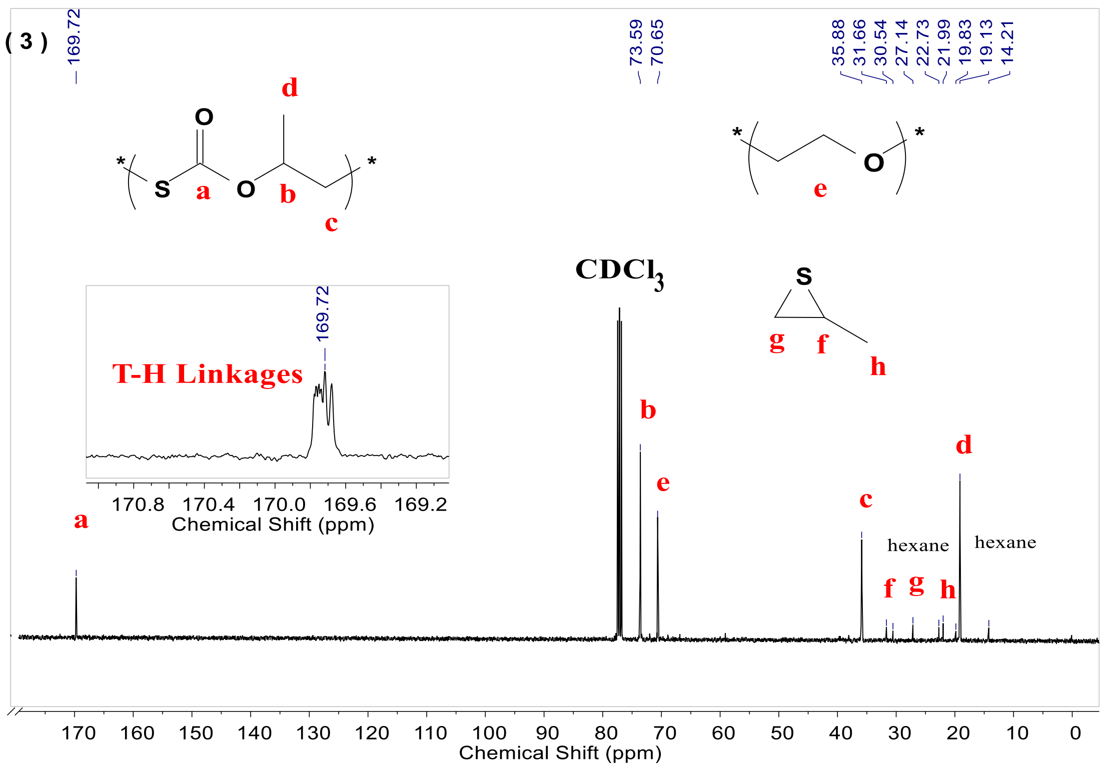

Figure S26. (1)^{1}H NMR spectrum of the crude product of entry 2, Table 3; (2) ^{1}H NMR spectrum of the purified product of entry 2, Table 3; (3) ^{13}C NMR spectrum of the purified product of entry 2, Table 3.

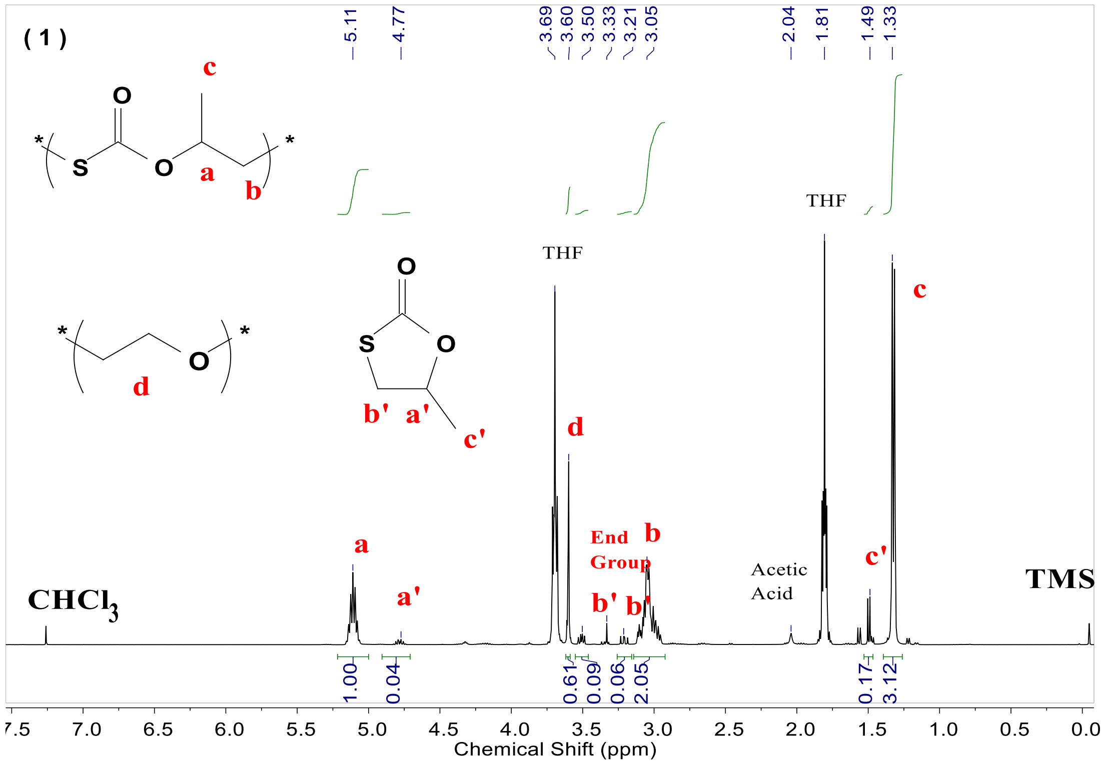

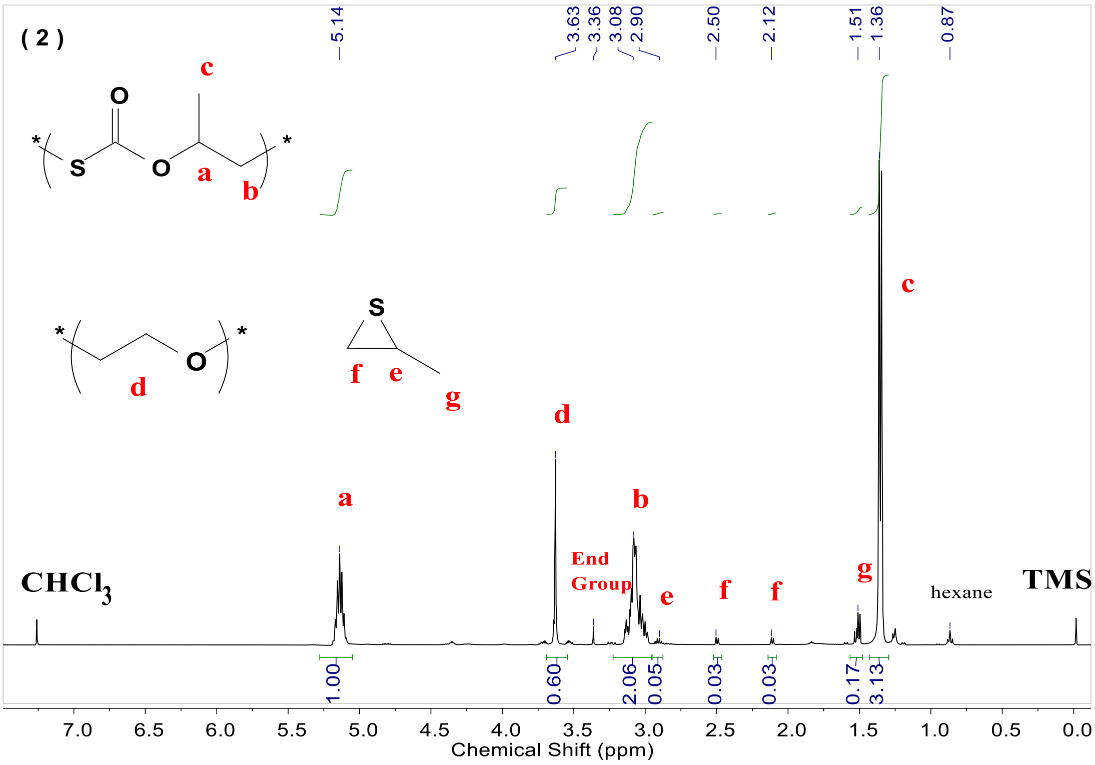

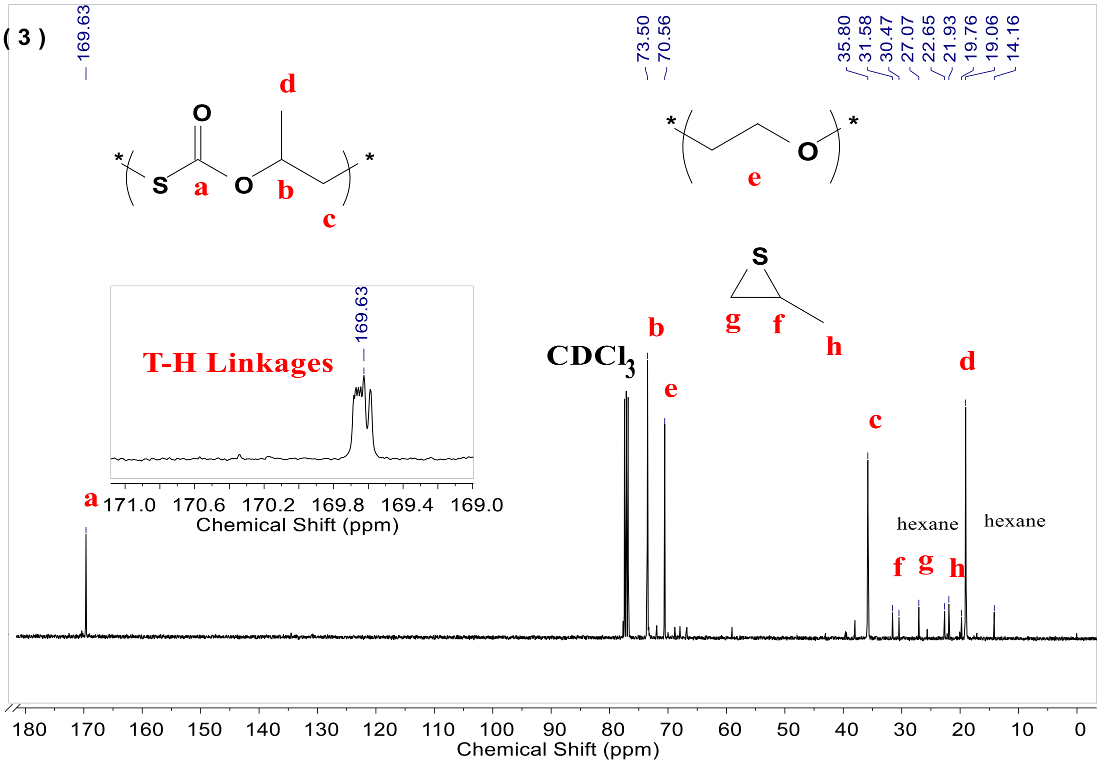

Figure S27. (1)^{1}H NMR spectrum of the crude product of entry 3, Table 3; (2) ^{1}H NMR spectrum of the purified product of entry 3, Table 3; (3) ^{13}C NMR spectrum of the purified product of entry 3, Table 3.

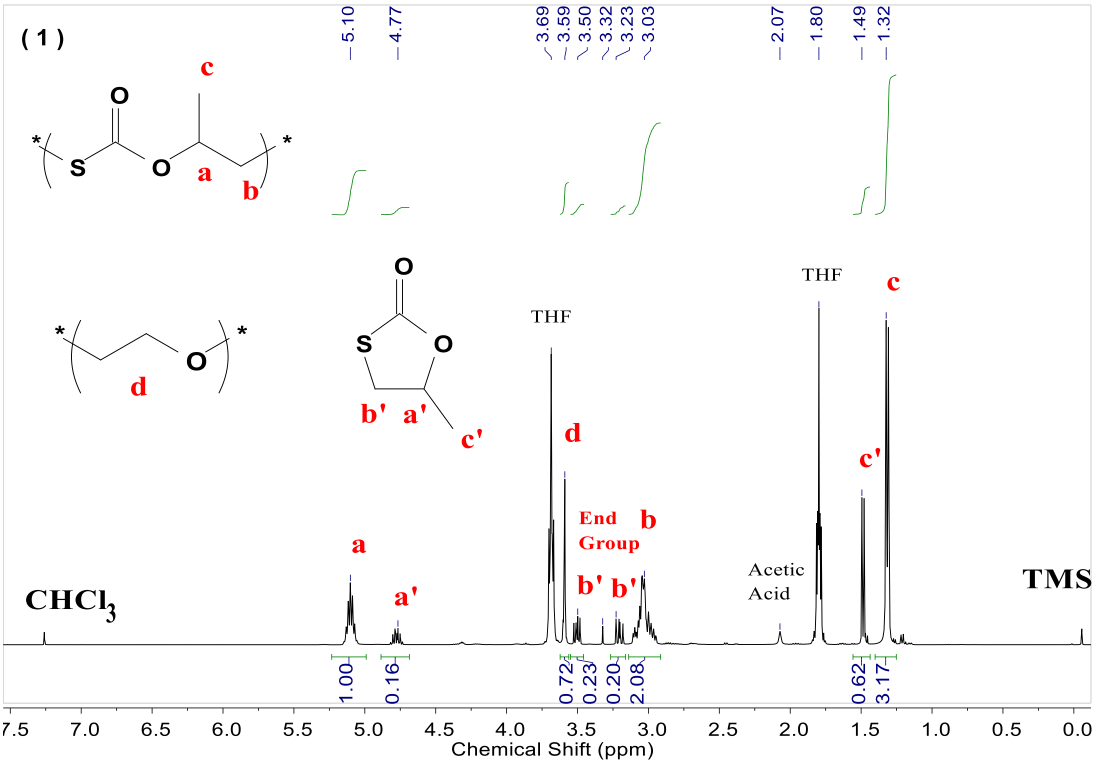
(1)
5.10
4.77
3.69
3.59
3.50
3.32
3.23
3.03
2.07
1.80
1.49
1.32
THF
THF
CHCl3
TMS
a
a'
d
End Group
b
b'
b'
c'
c
Acetic Acid
1.00
0.16
0.72
0.23
0.20
2.08
0.62
3.17
Chemical Shift (ppm)

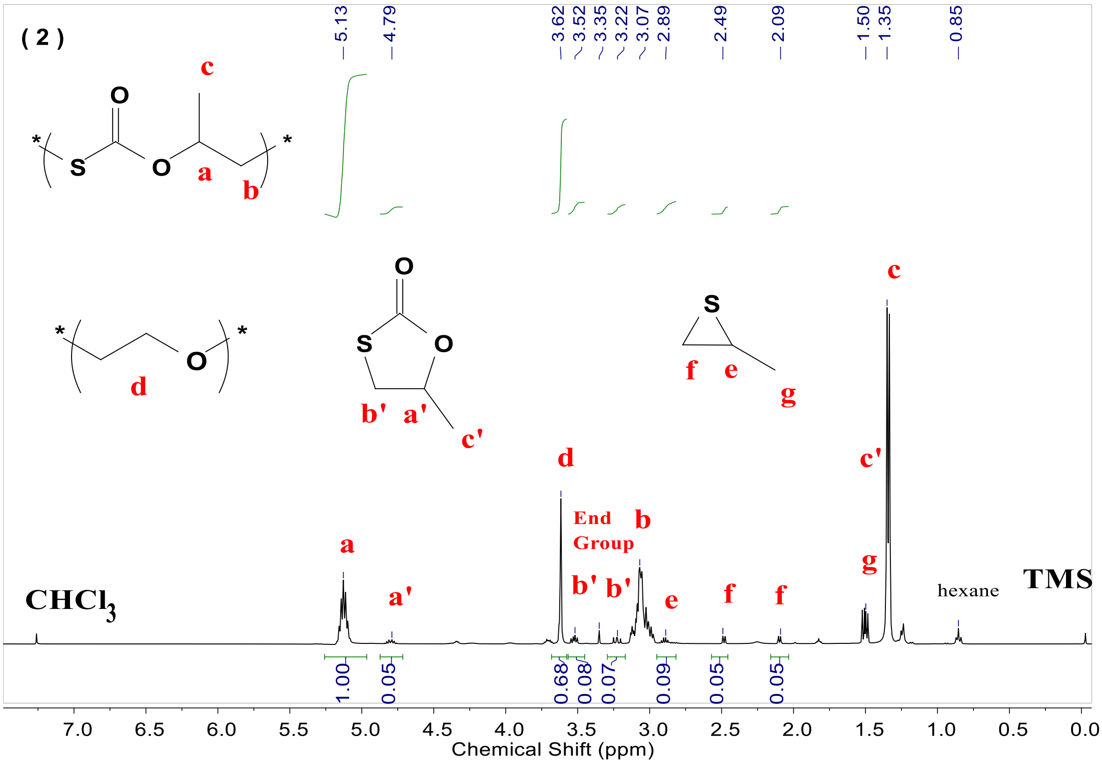
(2)
5.13
4.79
3.62
3.52
3.35
3.22
3.07
2.89
2.49
2.09
1.50
1.35
0.85
CHCl3
TMS
a
a'
d
End Group
b
b'
b'
e
f
f
g
c'
c
hexane
1.00
0.05
0.68
0.08
0.07
0.09
0.05
0.05
Chemical Shift (ppm)

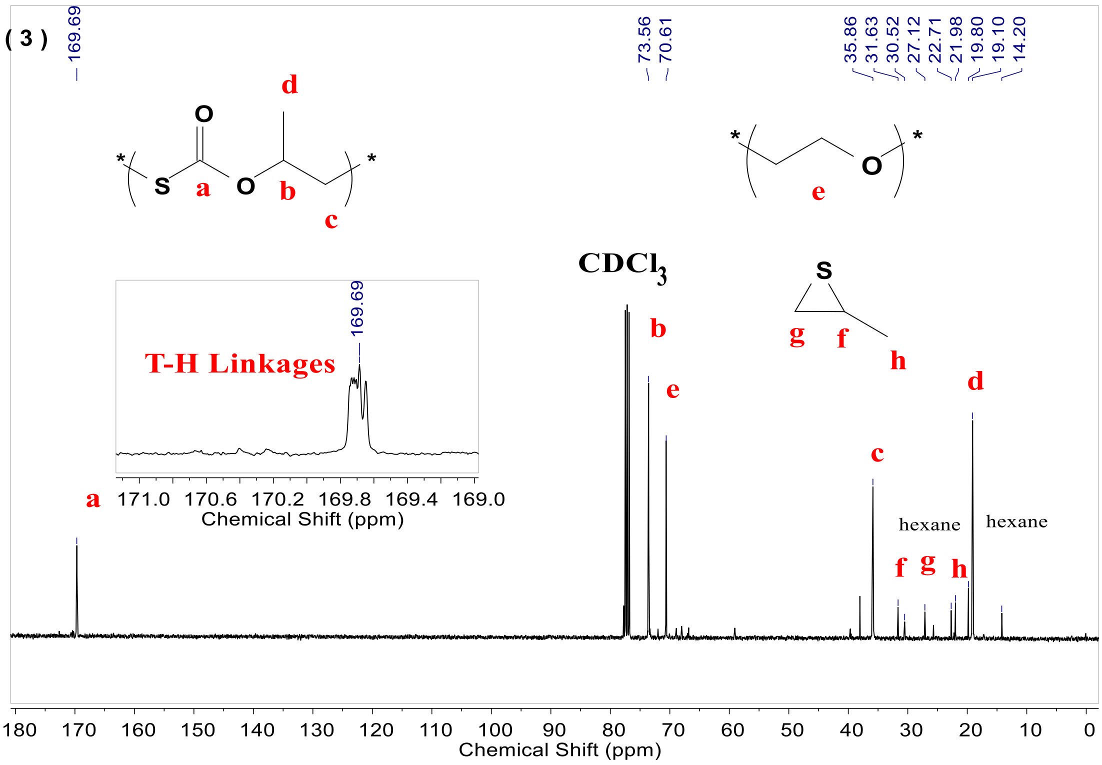

Figure S28. (1)^{1}H NMR spectrum of the crude product of entry 4, Table 3; (2) ^{1}H NMR spectrum of the purified product of entry 4, Table 3; (3) ^{13}C NMR spectrum of the purified product of entry 4, Table 3.

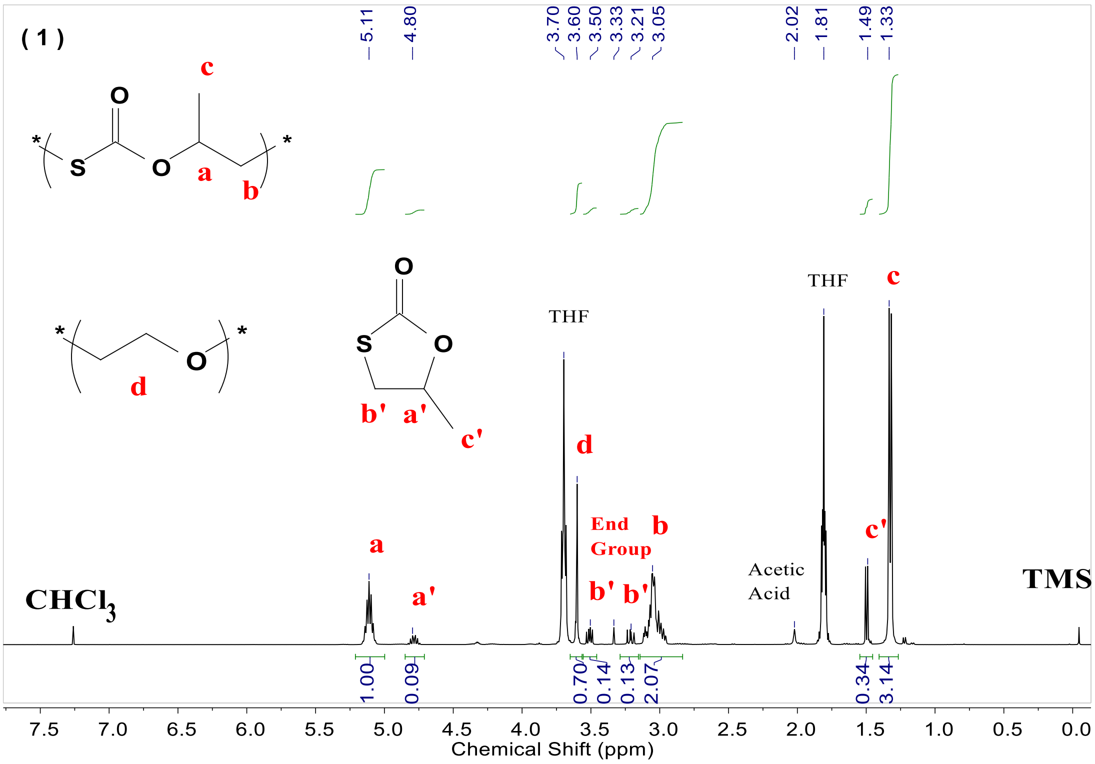

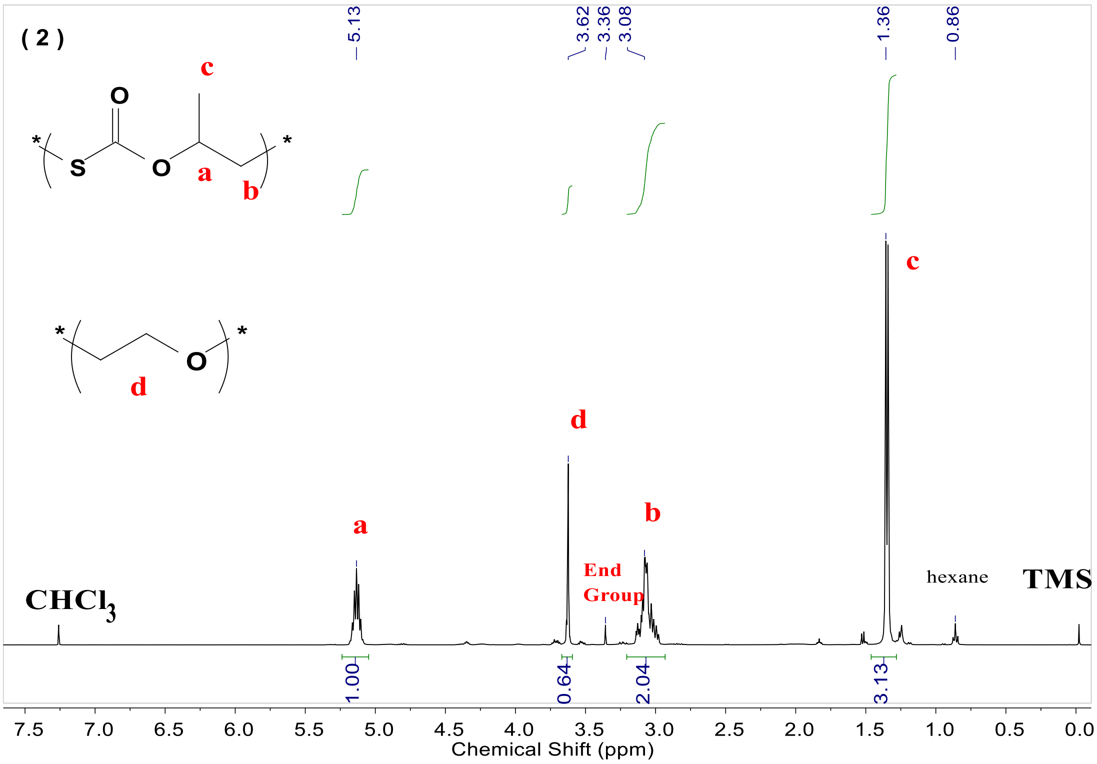

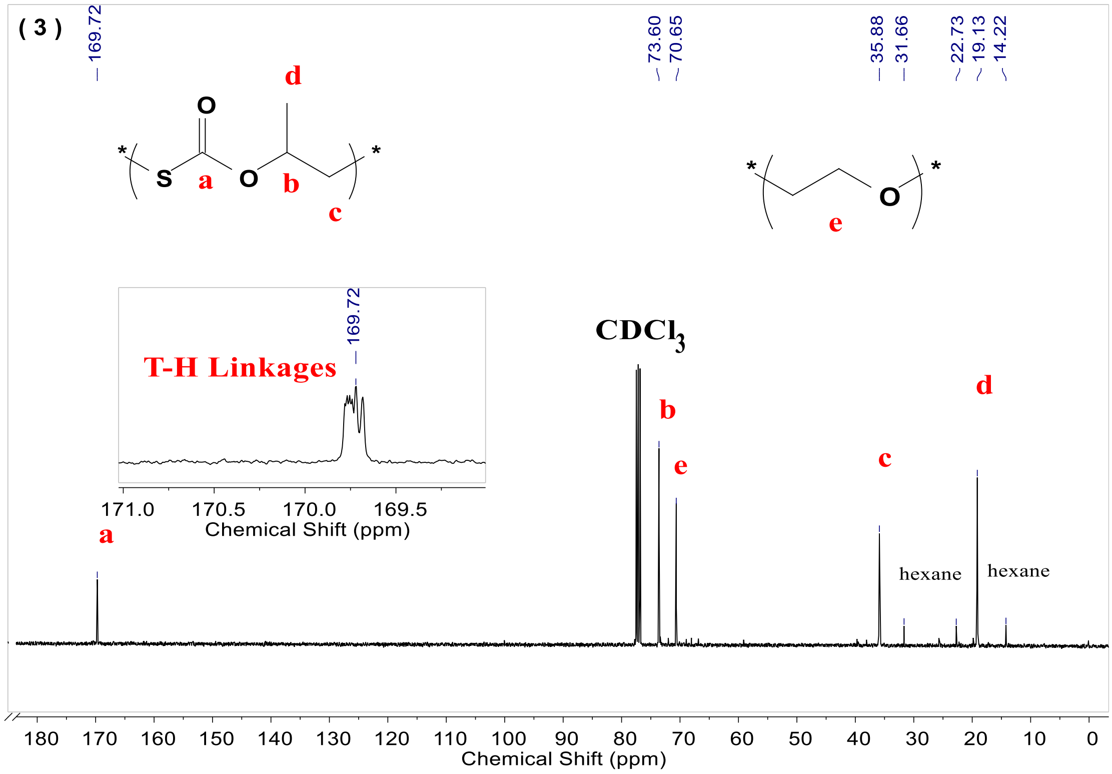

Figure S29. (1)^{1}H NMR spectrum of the crude product of entry 5, Table 3; (2) ^{1}H NMR spectrum of the purified product of entry 5, Table 3; (3) ^{13}C NMR spectrum of the purified product of entry 5, Table 3.

TGA curve of tri-block copolymer

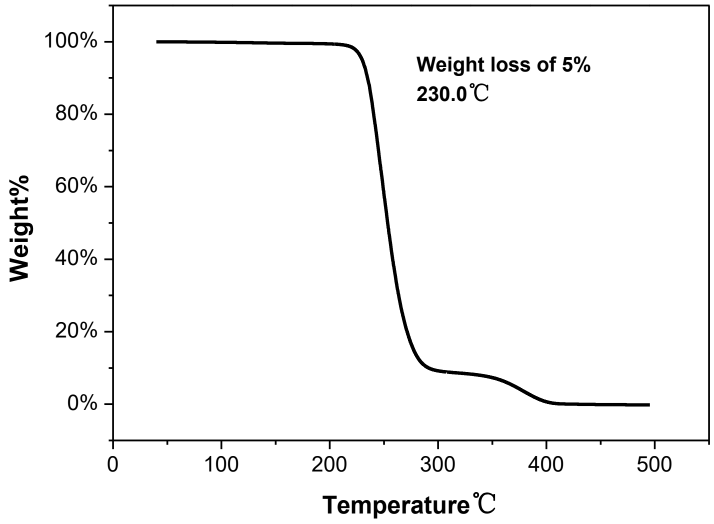

Figure S30. TGA curve of the *ABA* tri-block copolymer, entry 3, Table2
